# Diagnostic accuracy of physical examination findings for midfacial fractures: a systematic review and meta-analysis

**DOI:** 10.1007/s00784-022-04423-y

**Published:** 2022-03-17

**Authors:** Romke Rozema, Michiel H. J. Doff, Konstantina Delli, Frederik K. L. Spijkervet, Baucke van Minnen

**Affiliations:** 1 Department of Oral and Maxillofacial Surgery, University Medical Center Groningen, University of Groningen, Hanzeplein 1, 9700 RB Groningen, The Netherlands; 2 Department of Oral and Maxillofacial Surgery, Nij Smellinghe Hospital, Drachten, The Netherlands

**Keywords:** Emergency service, Hospital, Maxillofacial injuries, Physical examination, X-ray computed, Cone-beam computed tomography, Sensitivity and specificity, Systematic review [Publication Type], Maxillofacial fractures, Physical examination findings, Diagnostic accuracy, Sensitivity and specificity, Computed tomography, Cone beam computed tomography, Systematic review, Maxillofacial fractures, Physical examination findings, Diagnostic accuracy, Sensitivity and specificity, Computed tomography, Cone beam computed tomography, Systematic review

## Abstract

**Objectives:**

To conduct a systematic review and meta-analysis to assess the diagnostic accuracy of physical examination findings and related clinical decision aids for midfacial fractures in comparison to computed tomography and cone beam computed tomography.

**Material and methods:**

A systematic review was performed by searching the MEDLINE, Cochrane, EMBASE, and CINAHL databases. Risk of bias was assessed using the Quality Assessment of Diagnostic Accuracy Studies-2 tool. Pooled sensitivity, specificity, and diagnostic odds ratios with the corresponding 95% confidence intervals were calculated for each physical examination finding and reported clinical decision aids.

**Results:**

After screening 2367 records, 12 studies were included. High risk of patient selection bias was detected in three studies (25%). Additionally, high concerns regarding applicability were found for the patient selection in five studies (41.7%), and for the reference standard in eleven studies (91.7%). Of the total 42 individual physical examination findings, only 31 were suitable for a meta-analysis. High specificity and low sensitivity were found for most findings. The pooled diagnostic odds ratio ranged from 1.07 to 11.38. Clinical decision aids were reported by 8 studies, but none were constructed specifically for midfacial fractures.

**Conclusion:**

Based on the current available evidence, the absence of physical examination findings can successfully identify patients who do not have a midfacial fracture, but the presence of individual findings does not necessarily mean that the patient has a midfacial fracture. Although various clinical decision aids were presented, none focused on exclusively midfacial fractures.

**Clinical relevance:**

The diagnostic accuracy of physical examination findings can be used to diagnose a midfacial fracture so as to reduce unnecessary imaging, health care costs, and exposure to ionizing radiation.

**Supplementary Information:**

The online version contains supplementary material available at 10.1007/s00784-022-04423-y.

## Introduction

Midfacial trauma is a frequent cause for presentation at the emergency department [1–3]. The epidemiology of midfacial fractures varies depending on the population studied and may be the result of cultural, social, and environmental differences [4–6]. Leading causes include activities of daily living, sports, assault, and traffic-related accidents [4, 6]. Knowledge of these epidemiological properties may help the emergency physician to deliver more accurate care to the patients [5]. The assessment of midfacial trauma can be particularly challenging in a coexisting multi-trauma setting [5, 7–9]. Moreover, midfacial fractures present themselves with varying degrees of severity ranging from non-dislocated common nasal fractures to gross communition in Le Fort type fractures in which patients require immediate airway control due to midface instability and oropharyngeal obstruction [10–12]. Upon entering the emergency department, each trauma patient is assessed by the principles of Advanced Trauma Life Support (ATLS) to resuscitate and identify all the potential injuries, including fractures in the midfacial region [11–13].

The anatomy of the midface is known for its complexity [14]. The midfacial skeleton is often conceptualized as a framework of buttresses that are responsible for the width and height of the facial profile and establishes functional support for the dental arch and globe [14–16]. As a consequence, the midface is particularly known for its specific physical examination findings. Zygomaticomaxillary complex fractures, for example, are associated with sensory disturbances due to compression of the infra-orbital nerve [17–19]. Also, orbital floor fractures are known to cause entrapment of the inferior rectus muscle leading to upward gaze limitations and diplopia [20]. In addition, the broad range of potential fracture patterns, including frontal sinus, maxillary sinus, nasal bone, nasoorbitoethmoid complex, Le Fort I, II, III type and maxillary dentoalveolar complex fractures can complicate the physical examination [6, 21]. Understanding these fracture patterns is necessary as they are related to particular physical examination findings which are used to guide the need for radiological imaging.

Computed tomography (CT) and cone beam computed tomography (CBCT) are considered the gold-standard imaging modalities for the diagnosis of midfacial fractures [2, 5, 22–27]. The scanners produce volume datasets with submillimetre-sized voxels in all dimensions [22, 28]. The image data can be used for orthogonal plane reconstruction and three-dimensional volume rendering [29–32]. Both scanning systems area associated with risks related to exposure to ionizing radiation [25, 29, 33–37], which is of concern because of the exponential increase in the use of these systems over the last few decades. The estimated effective radiation dose of scan protocols for midface trauma is considered to be 0.9 to 3.6 mSv [25, 36, 38]. The effective dose of a CBCT is known to be lower, ranging from 0.08 to 0.21 mSv on average, depending on the field of view that is used [34]. However, the effective dose of both a CT and CBCT can vary significantly based on a multitude of factors such as the system type, scan range, size of the patient and scan protocol parameters [25, 34, 36, 39]. Hence, the interest in investigating whether physical examinations can be used to diagnose a fracture so as to reduce unnecessary imaging, health care costs and exposure to ionizing radiation [40, 41].

Although oral and maxillofacial surgeons are specifically trained to assess maxillofacial trauma patients, the initial diagnostic management is mostly performed by emergency physicians and specialized trauma surgeons [1, 5]. An awareness of how physical examination findings can predict midfacial fractures would enable adequate stratification of patients requiring radiological imaging. To date, no systematic review has been published on this topic. The aim of this systematic review and meta-analysis, thus, was to assess the diagnostic accuracy of physical examination findings and related clinical decision aids, in comparison to CT and CBCT, for the diagnosis of midfacial fractures.

## Material and methods

### Protocol

This systematic review was conducted following the recommendations of the Cochrane Handbook for Systematic Reviews of Interventions and reported according to the Preferred Reporting Items for a Systematic Review and Meta-Analysis of Diagnostic Test Accuracy Studies (PRISMA-DTA) [42, 43]. The study protocol was registered in the international prospective register of systematic reviews (PROSPERO, registration number CRD210040).

### Search strategy

An initial literature search was conducted on March 11, 2020, and updated on March 23, 2021, using the electronic databases of MEDLINE, EMBASE, CINAHL, and Cochrane Controlled Trial Register. Relevant search terms regarding midfacial fractures, physical examination findings, and their diagnostic accuracy were used and matched to relevant MeSH (MEDLINE, Cochrane) and EMTREE (EMBASE) terms, and to free text words according to the syntax rules of each database (Supplementary material S1). The search strategy was conducted in collaboration with a medical information specialist. In addition, the references of the included studies were screened.

### Study eligibility

The results of the literature search were imported into an EndNote X9.2 software environment (Clarivate Analytics, Philadelphia, Pennsylvania, USA) and duplicates were removed. The research question was defined using the PICOS format and, subsequently, the inclusion and exclusion criteria were determined (Table 1). The publications were assessed for eligibility in two rounds. In the first round, two reviewers (RR and MD) independently assessed the titles and abstracts according to the inclusion and exclusion criteria. The publications were allocated as “included” or “excluded” and in case of an indecisive verdict, publications were included for full text assessment. Publications selected for full text selection were independently assessed by the same two reviewers for final inclusion using the same selection criteria. After each selection round, discrepancies between the two reviewers were resolved in a consensus meeting. A third reviewer (BvM) was consulted to give a final judgement on any persisting disagreement. The interobserver agreement was calculated as the percentage of agreement, Cohen’s κ coefficient and Gwet’s AC1 statistic [44–46].

**Table 1:** Inclusion and exclusion criteria

Inclusion criteria
Population
1. Patients with a midfacial trauma 2. Mean or median age of patients ≥ 16 years 3. Admission to emergency department or outpatient clinic
Index test
4. Physical examination findings dedicated to the midfacial region and diagnostic accuracy for midfacial fractures (e.g., any changes to the visual appearance, findings related to the nasal and ocular assessment, intra-oral related changes, dental and occlusal abnormalities, functional changes and findings related to palpation)
Type of outcome measures
5. Midfacial fractures (e.g., frontal sinus, maxillary sinus, nasal, nasoorbitoethmoid, zygomaticomaxillary, orbital, maxillary or Le Fort type fractures) diagnosed using: a. Computed Tomography (CT) b. Cone Beam Computed Tomography (CBCT)
Data
6. Availability of sensitivity, specificity, pre-test probability, positive predictive value, negative predictive value, positive likelihood ratio, negative likelihood ratio, diagnostic odds ratio or a ROC/AUC curve or enough data should be available to construct two-by-two contingency tables to compute any of these statistics 7. Study design a. Cohort b. Case control c. Case report (≥ 10 patents) d. Diagnostic Randomized Controlled Trials 8. Full text availability 9. No language or time restrictions
Exclusion criteria
1. Case reports (< 10 patients), expert opinions, conference abstracts, reviews and systematic reviews

### Risk of bias assessment

The risk of bias of all the included studies was independently assessed by the same two reviewers using the Quality Assessment of Diagnostic Accuracy Studies 2 (QUADAS-2) tool [47]. This tool consists of four key domains covering patient selection, index test, reference standard, and flow and timing each including signaling questions focusing on the judgment of bias and concerns regarding applicability. A version applicable to this review is provided in Supplementary material S2. Disagreements were resolved through discussion.

### Data collection

Data were extracted using a pre-defined standardized form including the year of publication, study design, study set-up, single-center, or multi-center study design, trauma center level according to the American College of Surgeons classification [48], the studies patient population, patient demographics, level of consciousness according the Glasgow Coma Scale (GCS), the reference test used, fracture prevalence, the type of fracture outcome, reported physical examination findings (i.e., any finding related to the visual appearance of the patient, outcomes of the nasal and ocular assessment, intra-oral examination, sensory disturbances, and to palpation of the midface) and any proposed clinical decision aids developed from a combination of the reported physical examination findings. Only those physical examination findings that were specifically related to the midfacial region were collected. Two by two tables were constructed. If insufficient data were reported to produce two-by-two tables, backward calculations were performed using the provided sensitivity, specificity, pre-test probability, positive predictive value, negative predictive value, positive likelihood ratio, and negative likelihood ratio with the corresponding 95% confidence intervals [49]. The authors of the included studies were contacted in case of missing data or inconsistencies in the calculations by means of a minimum of two email attempts.

### Statistical analysis

Interobserver agreement was calculated using the Statistical Package for the Social Sciences version 23 (SPSS, IBM Corp., Armonk, New York, USA). A meta-analysis was performed to calculate the pooled sensitivity, specificity and diagnostics odds ratio using R statistics package for Meta-Analysis of Diagnostic Accuracy, for all the physical examination findings that were reported more than once for the same fracture outcome (MADA version 0.5.10, R Foundation for Statistical Computing, Vienna, Austria) [50]. Physical examination findings were only combined if the reported phraseology was plausibly about the same finding (e.g., infra-orbital nerve hypoesthesia and reduced sensation in the maxillary division of the trigeminal nerve). Regarding the diagnostic odds ratio calculations, 0.5 was added to all the cells of the contingency table in case of a zero cell count [51]. Testing for publication bias was performed using Deek’s funnel plots asymmetry test by a regression of the diagnostic log odds ratio against the inverse of the square root of the effective sample size [52, 53]. The statistical significance of the slope coefficient was defined as a *p*-value < 0.05. A meta-regression analysis was undertaken if more than ten studies reported physical examination findings with the same outcome.

## Results

### Study identification and selection

**Fig. 1:**
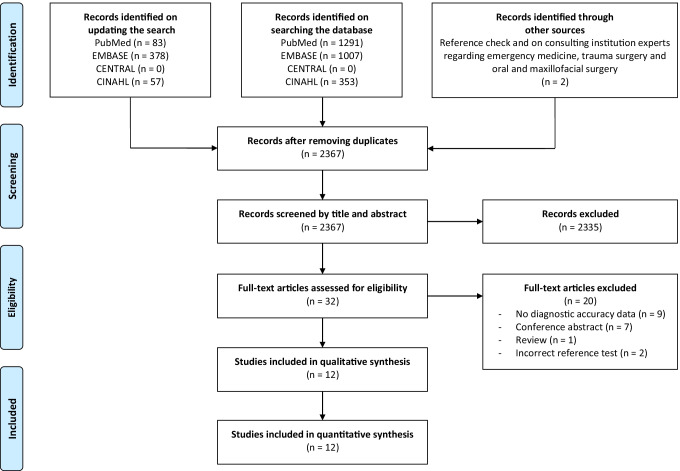
Flowchart of the study identification and selection process

The initial and updated literature search identified a total of 3171 publications (Fig. 1). After removing the duplicates, 2367 publications were screened by title and abstract. The percentage of agreement, kappa, and Gwet’s AC1 statistic were 98%, 0.55, and 0.98, respectively. A remaining total of 32 publications was eligible for full text screening. Twenty articles were excluded because they did not fulfil the inclusion or exclusion criteria (Supplementary material S3). The percentage of agreement, kappa, and Gwet’s AC1 statistic of the full text selections were 97%, 0.93, and 0.94, respectively. After the second round, a total of 12 publications were finally included for both qualitative and quantitative syntheses. It was not necessary to consult the third reviewer for a consensus.

### Methodological quality

**Fig. 2:**
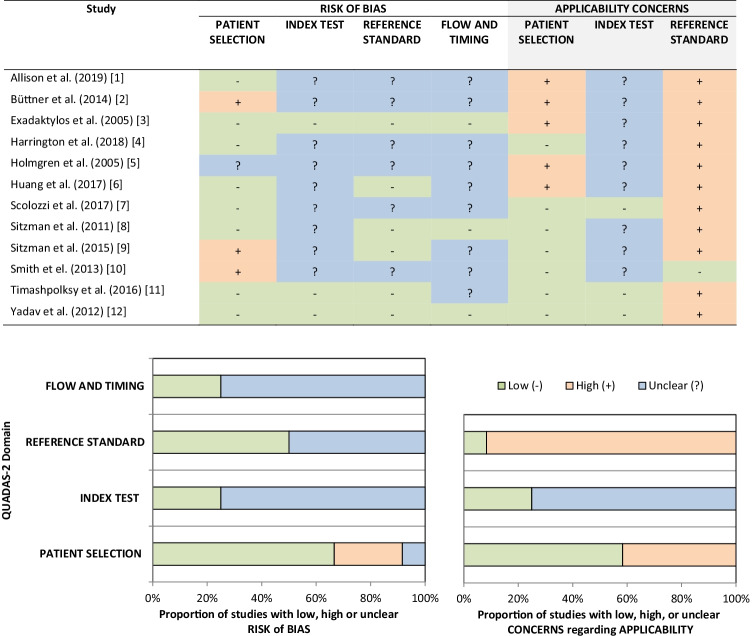
Risk of bias assessment

Figure 2 presents the quality assessment of the included studies according to the QUADAS-2 tool. High risk of bias in patient selection was detected in three studies (25%). Unclear risk of bias was found for the “index test” (75%), “references test” (50%), and “flow and timing” (75%) domains of the majority of the studies. Additionally, high concerns regarding applicability were found for “patient selection” in five studies (41.7%) and “reference standard” in eleven studies (91.7%), whereas the “index test” was unclear for most of the studies (75%).

### Study characteristics

The included publications consisted of eight retrospective studies, three prospective studies, and one case control study (Table 2). All 12 studies included emergency department patients; eleven studies investigated patients from a single center and one study had patients from two centers. Among the single-center studies, eight studies included patients from level I trauma centers, two studies included patients from level II trauma centers and one study included patients from a level III center. The two-center study included patients from both a level I and II trauma center.

**Table 2:** Study characteristics

Author	Year	Study design	Study set-up	Setting	Center level*	Patient population	Patients (n)	Male/female (n/n)	Age median or mean (yr.)	Age range (yr.)	GCS	Reference test	Fracture prev. (n (%))	Fracture outcomes
Holmgren et al. [54]	2005	Retro	Single-center	ED	Level I	Head and orbital trauma patients	777	564/213	32.4 (mean)	-	-	CT	477 (61.4)	Midfacial and mandibular fractures
Exadaktylos et al. [55]	2005	Prosp.	Single-center	ED	Level I	Head and orbital trauma patients	600	440/160	45.3 (mean)	12–86	3–15	CT	118 (19.7)	Orbital fractures
Sitzman et al. [56]	2011	Retro	Single-center	ED	Level I	Maxillofacial trauma patients	525	380/145	28 (median)	1–93	3–15	CT	332 (63.2)	Midfacial and mandibular fractures
Yadav et al. [57]	2012	Prosp.	Two-center	ED	Level I/II	Orbital trauma patients	2262	1544/718	38 (median)	-		CT	360 (15.9)	Orbital fractures
Smith et el. [58]	2013	Case–control	Single-center	ED	Level I	Midfacial trauma patients	166	105/61	47–50 (median)	18-?	9–15	CT	83 (50)	Midfacial fractures
Büttner et al. [59]	2014	Retro	Single-center	ED	Level I	Minor head injury patients with a black eye	1676	1102/574	51 (mean)	16–99	13–15	CT	1144 (68.3)	Midfacial and mandibular fractures
Sitzman et al. [60]	2015	Retro	Single-center	ED	Level I	Maxillofacial trauma patients	179	132/47	31 (median)	0–91	3–15	CT	116 (64.8)	Midfacial and mandibular fractures
Timashpolksy et al. [61]	2016	Prosp.	Single-center	ED	Level I	Maxillofacial trauma patients	57	44/13	40.04 (mean)	-	-	CT	52 (91.2)	Midfacial and mandibular fractures
Scolozzi et al. [62]	2017	Retro	Single-center	ED	Level II	Orbital trauma patients	912	632/280	46.6 (mean)	-	-	CT	701 (76.9)	Orbital fractures
Huang et al. [63]	2017	Retro	Single-center	ED	Level II	Traumatic brain injury patients with facial trauma	1649	918/713	53.1 (mean)	20–101	3–15	CT	200 (13.8)	Midfacial and mandibular fractures
Harrington et al. [64]	2018	Retro	Single-center	ED	Level I	Maxillofacial trauma patients	167	105/62	50.5 (mean)	-	3–15	CT	99 (59.3)	Midfacial and mandibular fractures
Allison et al. [65]	2019	Retro	Single-center	ED	Level III	Head and orbital trauma patients	47	41/6	40.6 (mean)	-	-	CT	35 (74.5)	Orbital fractures

Abbreviations: *GCS* Glasgow Coma Scale; *Prev* prevalence; *Retro* retrospective cohort study; *Prosp* prospective cohort study; *ED* emergency department; *CT* computed tomography

### Patient characteristics

The number of patients in the studies ranged from 47 to 2262, resulting in a total of 9017 patients of whom 6007 were male and 3010 female. The reported mean age was 37.1 years, and the reported median age ranged from 28 to 50. The study population included midfacial trauma patients (*n* = 1) [58], maxillofacial trauma patients (*n* = 4) [56, 60, 61, 64], orbital trauma patients (*n* = 2) [57, 62], head and orbital trauma patients (*n* = 3) [54, 55, 65], minor head injury patients with a black eye (*n* = 1) [59], and traumatic brain injury patients with facial trauma (*n* = 1) [63]. All the studies had used CT as a reference test and thus no studies were included where CBCT was used as a reference test. Any midfacial fracture was used as an outcome by one study [58], whereas any midfacial or mandibular fracture was used as an outcome by seven studies [54, 56, 59–61, 63, 64], and orbital fracture was used as an outcome by four studies [55, 57, 62, 65]. In one study, midfacial and mandibular fracture outcomes were stratified as frontal sinus, zygoma, orbital floor, naso-ethmoidal, nasal, maxilla, and mandibular fractures [61]. The fracture prevalence ranged from 13.8 to 91.2%, resulting in an average of 41.2%.

### Physical examination findings

**Fig. 3:**
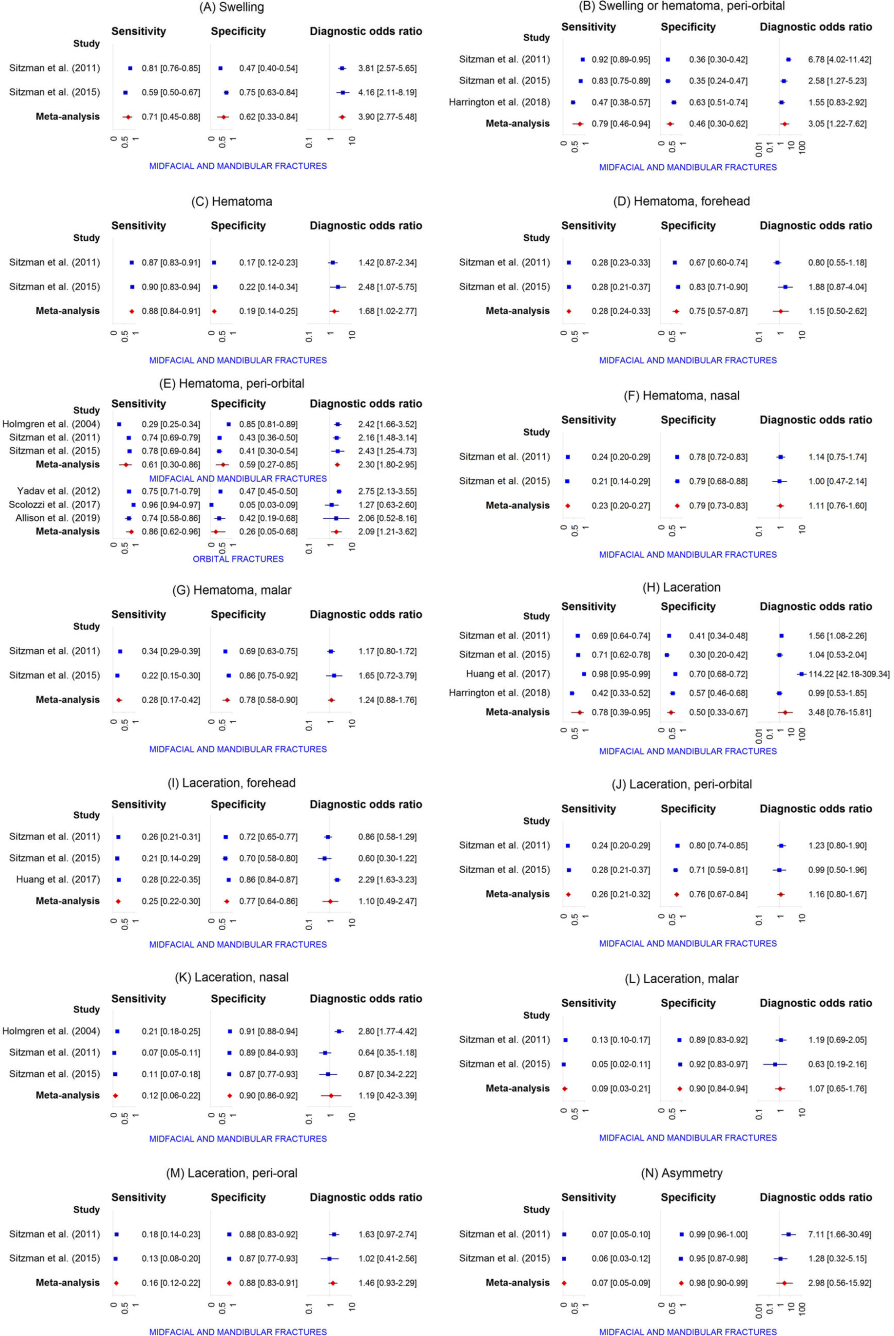

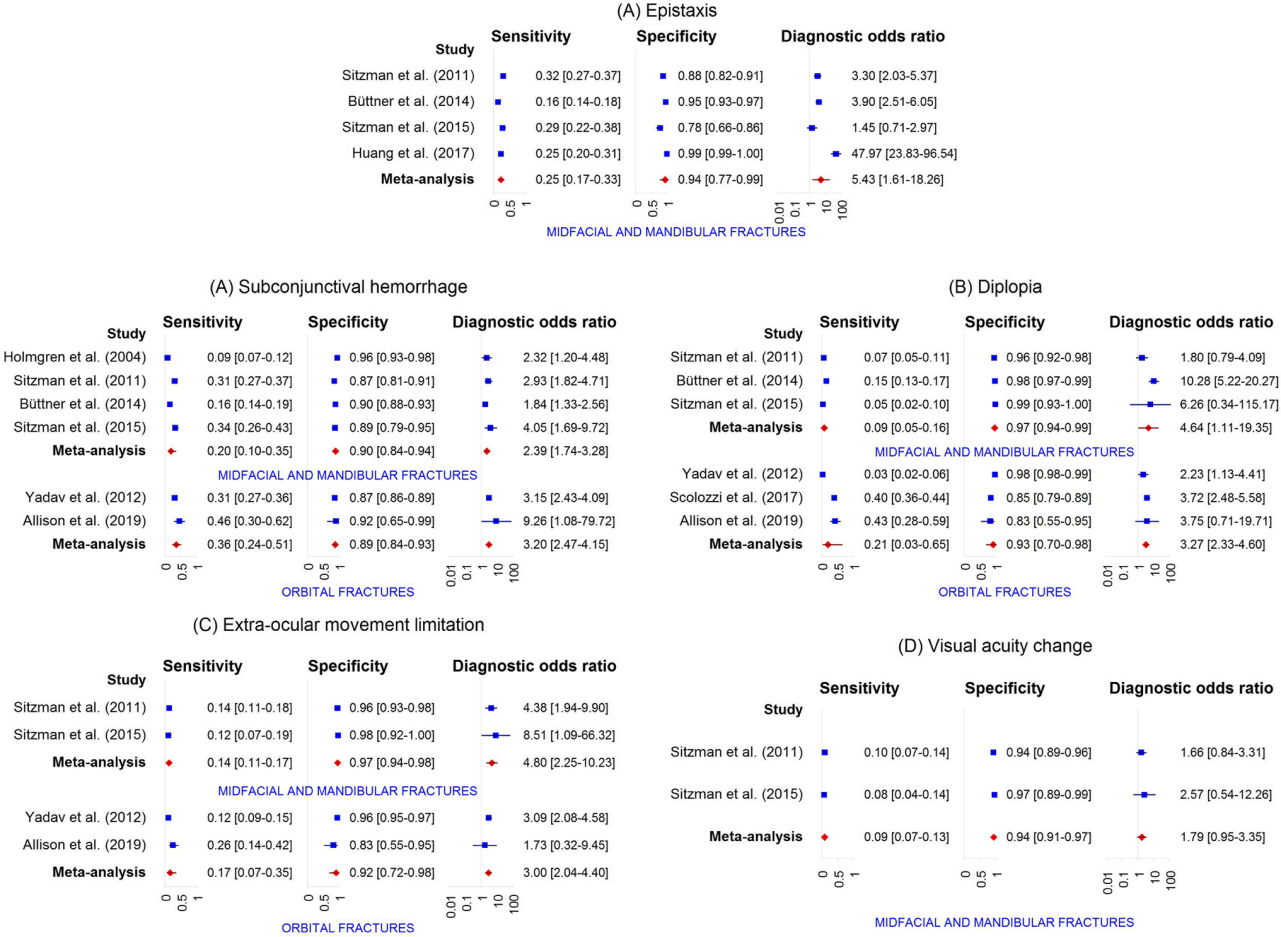

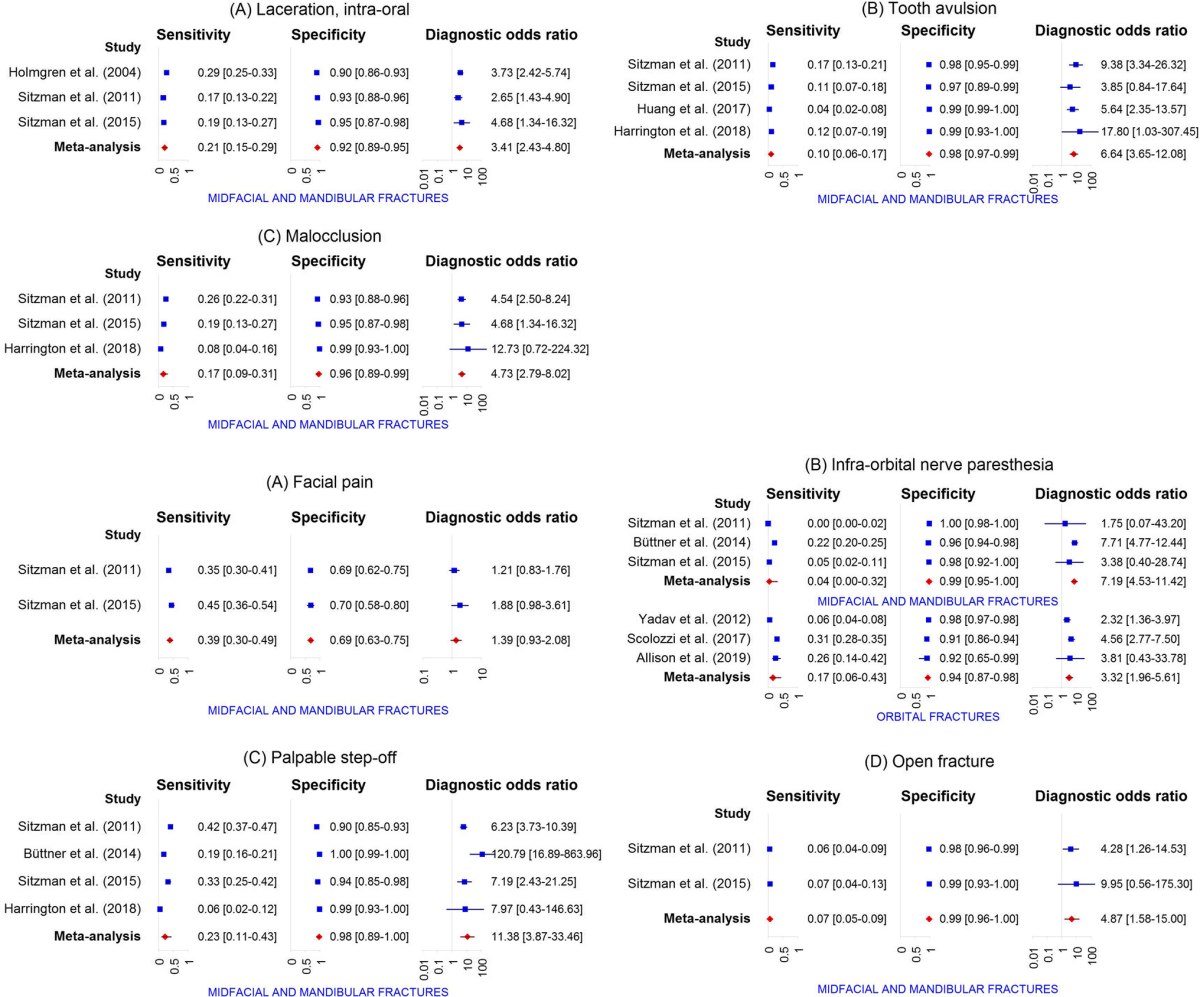
**a** Forest plots showing study-specific and pooled specificity, sensitivity, and diagnostic odds ratio of the physical examination findings related to visual appearance for (a) swelling, (b) peri-orbital swelling or hematoma, (c) hematoma, (d) forehead hematoma, (e) peri-orbital hematoma, (f) nasal hematoma, (g) malar hematoma, (h) laceration, (i) forehead laceration, (j) peri-orbital laceration, (k) nasal laceration, (l) malar laceration, (m) peri-oral laceration, (n) asymmetry in diagnosing midfacial fractures. **b** Forest plots showing study-specific and pooled specificity, sensitivity, and diagnostic odds ratio of the physical examination findings related to nasal assessment for (a) epistaxis in diagnosing midfacial fractures. **c** Forest plots showing study-specific and pooled specificity, sensitivity and diagnostic odds ratio of the physical examination findings related to ocular assessment for (a) subconjuctival hemorrhage, (b) diplopia, (c) extra-ocular movement limitation, and (d) visual acuity change in diagnosing midfacial fractures. **d** Forest plots showing study-specific and pooled specificity, sensitivity and diagnostic odds ratio of the physical examination findings related to intra-oral assessment for (a) intra-oral laceration, (b) tooth avulsion and (c) malocclusion in diagnosing midfacial fractures. **e** Forest plots showing study-specific and pooled specificity, sensitivity and diagnostic odds ratio of the physical examination findings related to functional and palpation assessment for (a) facial pain, (b) infra-orbital nerve paresthesia, (c) palpable step-off, and (d) open fracture in diagnosing midfacial fractures

A total of 42 distinct physical examination findings were identified and categorized into 5 distinct groups: visual appearance, nasal assessment, ocular assessment, intra-oral assessment, and findings related to functional and palpation assessment. The diagnostic accuracy of each individual physical examination finding is presented in Table 3. For 30 findings, the diagnostic accuracy was reported in more than one study. Meta-analysis was feasible for a total of 31 physical examination findings (Fig. 3).

**Table 3:** Diagnostic accuracy of individual physical examination findings

Physical examination finding	Authors and reference	Fracture outcome	Sens. (95% CI)	Spec. (95% CI)	Pre-test prob. (95% CI)	PPV (95% CI)	NPV (95% CI)	LR + (95% CI)	LR- (95% CI)
Visual appearance									
Swelling	Sitzman et al. [56]	Midfacial and mandibular fractures	81.0 (76.5–84.9)	47.2 (40.2–54.2)	63.2 (59.0–67.3)	72.5 (67.8–76.8)	59.1 (51.2–66.5)	1.5 (1.3–1.8)	0.4 (0.3–0.5)
	Sitzman et al. [60]	Midfacial and mandibular fractures	58.6 (49.5–67.2)	74.6 (62.7–83.7)	64.8 (57.6–71.4)	81.0 (71.3–87.9)	49.5 (39.6–59.4)	2.3 (1.5–3.6)	0.6 (0.4–0.7)
Swelling or hematoma	Timashpolsky et al. [61]	Orbital floor fractures	64.0 (44.5–79.8)	81.3 (64.7–91.1)	43.9 (31.8–56.7)	72.7 (51.8–86.8)	74.3 (57.9–85.8)	3.4 (1.6–7.4)	0.4 (0.3–0.8)
	Timashpolsky et al. [61]	Zygoma fractures	16.7 (5.8–39.2)	84.6 (70.3–92.8)	31.6 (21.0–44.5)	33.3 (12.1–64.6)	68.8 (54.7–80.1)	1.1 (0.3–3.9)	1.0 (0.8–1.3)
Swelling, peri-orbital	Yadav et al. [57]	Orbital fractures	78.3 (73.8–82.3)	43.7 (41.5–46.0)	15.9 (14.5–17.5)	20.9 (18.8–23.1)	91.4 (89.4–93.1)	1.4 (1.3–1.5)	0.5 (0.4–0.6)
Swelling or hematoma, peri-orbital	Harrington et al. [64]	Midfacial and mandibular fractures	47.5 (37.9–57.2)	63.2 (51.4–73.7)	59.3 (51.7–66.4)	65.3 (53.8–75.2)	45.3 (35.6–55.3)	1.3 (0.9–1.9)	0.8 (0.6–1.1)
	Sitzman et al. [56]	Midfacial and mandibular fractures	92.5 (88.7–95.1)	35.5 (29.9–41.5)	50.7 (46.4–54.9)	59.6 (54.8–64.2)	82.1 (74.0–88.1)	1.4 (1.3–1.6)	0.2 (0.1–0.3)
	Sitzman et al. [60]	Midfacial and mandibular fractures	82.8 (74.9–88.6)	34.9 (24.3–47.2)	64.8 (57.6–71.4)	70.1 (61.9–77.1)	52.4 (37.7–66.6)	1.3 (1.0–1.6)	0.5 (0.3–0.8)
Hematoma	Sitzman et al. [56]	Midfacial and mandibular fractures	87.3 (83.3–90.5)	17.1 (12.4–23.0)	63.2 (59.0–67.3)	64.4 (59.9–68.7)	44.0 (33.3–55.3)	1.1 (1.0–1.1)	0.7 (0.5–1.1)
	Sitzman et al. [60]	Midfacial and mandibular fractures	89.7 (82.8–94.0)	22.2 (13.7–33.9)	64.8 (57.6–71.4)	68.0 (60.2–74.8)	53.8 (35.5–71.2)	1.2 (1.0–1.3)	0.5 (0.2–0.9)
Hematoma, forehead	Sitzman et al. [56]	Midfacial and mandibular fractures	28.0 (23.5–33.1)	67.4 (60.5–73.6)	63.2 (59.0–67.3)	59.6 (51.8–67.0)	35.2 (30.5–40.2)	0.9 (0.7–1.1)	1.1 (0.9–1.2)
	Sitzman et al. [60]	Midfacial and mandibular fractures	28.4 (21.0–37.2)	82.5 (71.4–90.0)	64.8 (57.6–71.4)	75.0 (60.6–85.4)	38.5 (30.7–46.9)	1.6 (0.9–3.0)	0.9 (0.7–1.0)
Hematoma, peri-orbital	Allison et al. [65]	Orbital fractures	74.3 (57.9–85.8)	41.7 (19.3–68.0)	74.5 (60.5–84.7)	78.8 (62.2–89.3)	35.7 (16.3–61.2)	1.3 (0.8–2.1)	0.6 (0.3–1.5)
	Holmgren et al. [54]	Midfacial and mandibular fractures	29.4 (25.4–33.6)	85.3 (80.9–88.9)	61.4 (57.9–64.7)	76.1 (69.4–81.7)	43.2 (39.2–47.2)	2.0 (1.5–2.7)	0.8 (0.8–0.9)
	Scolozzi et al. [62]	Orbital fractures	95.9 (94.1–97.1)	5.2 (2.9–9.1)	76.9 (74.0–79.5)	77.1 (74.2–79.7)	27.5 (16.1–42.8)	1.0 (1.0–1.0)	0.8 (0.4–1.6)
	Sitzman et al. [56]	Midfacial and mandibular fractures	74.1 (69.1–78.5)	43.0 (36.2–50.1)	63.2 (59.0–67.3)	69.1 (64.1–73.7)	49.1 (41.7–56.6)	1.3 (1.1–1.5)	0.6 (0.5–0.8)
	Sitzman et al. [60]	Midfacial and mandibular fractures	77.6 (69.2–84.2)	41.3 (30.0–53.6)	64.8 (57.6–71.4)	70.9 (62.4–78.1)	50.0 (36.9–63.1)	1.3 (1.1–1.7)	0.5 (0.3–0.9)
	Smith et al. [58]	Midfacial fractures	38.6 (28.8–49.3)	95.2 (88.3–98.1)	50.0 (42.5–57.5)	88.9 (74.7–95.6)	60.8 (52.2–68.7)	8.0 (3.0–21.6)	0.6 (0.5–0.8)
	Yadav et al. [57]	Orbital fractures	75.3 (70.6–79.5)	47.5 (45.2–49.7)	15.9 (14.5–17.5)	21.3 (19.2–23.7)	91.0 (89.1–92.7)	1.4 (1.3–1.5)	0.5 (0.4–0.6)
Hematoma, eyelid	Holmgren et al. [54]	Midfacial and mandibular fractures	2.7 (1.6–4.6)	98.7 (96.6–99.5)	61.4 (57.9–64.7)	76.5 (52.7–90.4)	38.9 (35.5–42.5)	2.0 (0.7–6.2)	1.0 (1.0–1.0)
	Exadaktylos et al. [55]	Orbital fractures	68.6 (59.8–76.3)	94.8 (92.5–96.5)	19.7 (16.7–23.0)	76.4 (67.5–83.5)	92.5 (89.8–94.5)	13.2 (8.9–19.8)	0.3 (0.3–0.4)
Hematoma, nasal	Sitzman et al. [56]	Midfacial and mandibular fractures	24.1 (19.8–29.0)	78.2 (71.9–83.5)	63.2 (59.0–67.3)	65.6 (56.8–73.4)	37.5 (32.9–42.3)	1.1 (0.8–1.5)	1.0 (0.9–1.1)
	Sitzman et al. [60]	Midfacial and mandibular fractures	20.7 (14.3–28.9)	79.4 (67.8–87.5)	64.8 (57.6–71.4)	64.9 (48.8–78.2)	35.2 (27.8–43.4)	1.0 (0.5–1.8)	1.0 (0.9–1.2)
Hematoma, malar	Sitzman et al. [56]	Midfacial and mandibular fractures	34.0 (29.1–39.3)	69.4 (62.6–75.5)	63.2 (59.0–67.3)	65.7 (58.3–72.4)	38.0 (33.1–43.1)	1.1 (0.9–1.4)	1.0 (0.8–1.1)
	Sitzman et al. [60]	Midfacial and mandibular fractures	21.6 (15.0–29.9)	85.7 (75.0–92.3)	64.8 (57.6–71.4)	73.5 (56.9–85.4)	37.2 (29.8–45.3)	1.5 (0.8–3.0)	0.9 (0.8–1.1)
Hematoma, facial or scalp	Holmgren et al. [54]	Midfacial and mandibular fractures	24.9 (21.3–29.0)	47.3 (41.8–53.0)	61.4 (57.9–64.7)	43.0 (37.3–48.8)	28.4 (24.6–32.5)	0.5 (0.4–0.6)	1.6 (1.4–1.8)
Laceration	Harrington et al. [64]	Midfacial and mandibular fractures	42.4 (33.2–52.3)	57.4 (45.5–68.4)	59.3 (51.7–66.4)	59.2 (47.5–69.8)	40.6 (31.3–50.6)	1.0 (0.7–1.4)	1.0 (0.8–1.3)
	Huang et al. [63]	Midfacial and mandibular fractures	98.0 (95.0–99.2)	70.0 (67.6–72.3)	12.1 (10.6–13.8)	31.1 (27.6–34.8)	99.6 (99.0–99.8)	3.3 (3.0–3.5)	0.0 (0.0–0.1)
	Sitzman et al. [56]	Midfacial and mandibular fractures	69.3 (64.1–74.0)	40.9 (34.2–48.0)	63.2 (59.0–67.3)	66.9 (61.7–71.6)	43.6 (36.6–50.9)	1.2 (1.0–1.3)	0.8 (0.6–0.9)
	Sitzman et al. [60]	Midfacial and mandibular fractures	70.7 (61.8–78.2)	30.2 (20.2–42.4)	64.8 (57.6–71.4)	65.1 (56.4–72.8)	35.8 (24.3–49.3)	1.0 (0.8–1.2)	1.0 (0.6–1.6)
Laceration, forehead	Huang et al. [63]	Midfacial and mandibular fractures	28.0 (22.2–34.6)	85.5 (83.6–87.2)	12.1 (10.6–13.8)	21.1 (16.6–26.3)	89.6 (87.9–91.1)	1.9 (1.5–2.5)	0.8 (0.8–0.9)
	Sitzman et al. [56]	Midfacial and mandibular fractures	25.6 (21.2–30.6)	71.5 (64.8–77.4)	63.2 (59.0–67.3)	60.7 (52.4–68.4)	35.8 (31.2–40.8)	0.9 (0.7–1.2)	1.0 (0.9–1.2)
	Sitzman et al. [60]	Midfacial and mandibular fractures	20.7 (14.3–28.9)	69.8 (57.6–79.8)	64.8 (57.6–71.4)	55.8 (41.1–69.6)	32.4 (25.1–40.6)	0.7 (0.4–1.2)	1.1 (0.9–1.4)
	Smith et el. [58]	Midfacial fractures	22.9 (15.2–33.0)	90.4 (82.1–95.0)	50.0 (42.5–57.5)	70.4 (51.5–84.1)	54.0 (45.7–62.0)	2.4 (1.1–5.1)	0.9 (0.7–1.0)
Laceration, peri-orbital	Sitzman et al. [56]	Midfacial and mandibular fractures	23.8 (19.5–28.7)	79.8 (73.6–84.9)	63.2 (59.0–67.3)	66.9 (58.0–74.8)	37.8 (33.3–42.6)	1.2 (0.8–1.7)	1.0 (0.9–1.0)
	Sitzman et al. [60]	Midfacial and mandibular fractures	28.4 (21.0–37.2)	71.4 (59.3–81.1)	64.8 (57.6–71.4)	64.7 (51.0–76.4)	35.2 (27.4–43.8)	1.0 (0.6–1.6)	1.0 (0.8–1.2)
	Yadav et al. [57]	Orbital fractures	32.8 (28.1–37.8)	77.4 (75.5–79.3)	15.9 (14.5–17.5)	21.6 (18.3–25.2)	85.9 (84.2–87.5)	1.5 (1.2–1.7)	0.9 (0.8–0.9)
Laceration, eyebrow	Holmgren et al. [54]	Midfacial and mandibular fractures	9.9 (7.5–12.9)	90.0 (86.1–92.9)	61.4 (57.9–64.7)	61.0 (49.9–71.2)	38.6 (35.0–42.2)	1.0 (0.6–1.5)	1.0 (1.0–1.1)
Laceration, eyelid	Holmgren et al. [54]	Midfacial and mandibular fractures	13.2 (10.5–16.5)	87.0 (82.7–90.3)	61.4 (57.9–64.7)	61.8 (52.1–70.6)	38.7 (35.1–42.4)	1.0 (0.7–1.5)	1.0 (0.9–1.1)
Laceration, conjunctival	Holmgren et al. [54]	Midfacial and mandibular fractures	0.6 (0.2–1.8)	99.0 (97.1–99.7)	61.4 (57.9–64.7)	50.0 (18.8–81.2)	38.5 (35.2–42.0)	0.6 (0.1–3.1)	1.0 (1.0–1.0)
Laceration, nasal	Sitzman et al. [56]	Midfacial and mandibular fractures	7.2 (4.9–10.5)	89.1 (83.9–92.8)	63.2 (59.0–67.3)	53.3 (39.1–67.1)	35.8 (31.7–40.2)	0.7 (0.4–1.2)	1.0 (1.0–1.1)
	Sitzman et al. [60]	Midfacial and mandibular fractures	11.2 (6.7–18.2)	87.3 (76.9–93.4)	64.8 (57.6–71.4)	61.9 (40.9–79.2)	34.8 (27.8–42.5)	0.9 (0.4–2.0)	1.0 (0.9–1.1)
	Holmgren et al. [54]	Midfacial and mandibular fractures	21.0 (17.6–24.8)	91.3 (87.6–94.0)	61.4 (57.9–64.7)	79.4 (71.5–85.5)	42.1 (38.4–45.9)	2.4 (1.6–3.6)	0.9 (0.8–0.9)
Laceration, malar	Sitzman et al. [56]	Midfacial and mandibular fractures	13.3 (10.0–17.3)	88.6 (83.3–92.4)	63.2 (59.0–67.3)	66.7 (54.7–76.8)	37.3 (33.0–41.8)	1.2 (0.7–1.9)	1.0 (0.9–1.0)
	Sitzman et al. [60]	Midfacial and mandibular fractures	5.2 (2.4–10.8)	92.1 (82.7–96.6)	64.8 (57.6–71.4)	54.5 (28.0–78.7)	34.5 (27.8–42.0)	0.7 (0.2–2.1)	1.0 (0.9–1.1)
Laceration, peri-oral	Sitzman et al. [56]	Midfacial and mandibular fractures	18.1 (14.3–22.6)	88.1 (82.8–91.9)	63.2 (59.0–67.3)	72.3 (61.8–80.8)	38.5 (34.0–43.1)	1.5 (1.0–2.4)	0.9 (0.9–1.0)
	Sitzman et al. [60]	Midfacial and mandibular fractures	12.9 (8.0–20.2)	87.3 (76.9–93.4)	64.8 (57.6–71.4)	65.2 (44.9–81.2)	35.3 (28.2–43.0)	1.0 (0.5–2.3)	1.0 (0.9–1.1)
Laceration, lip	Holmgren et al. [54]	Midfacial and mandibular fractures	26.2 (22.5–30.3)	85.3 (80.9–88.9)	61.4 (57.9–64.7)	74.0 (66.9–80.0)	42.1 (38.2–46.1)	1.8 (1.3–2.4)	0.9 (0.8–0.9)
Asymmetry	Sitzman et al. [56]	Midfacial and mandibular fractures	6.9 (4.7–10.2)	99.0 (96.3–99.7)	63.2 (59.0–67.3)	92.0 (75.0–97.8)	38.2 (34.0–42.5)	6.7 (1.6–28.0)	0.9 (0.9–1.0)
	Sitzman et al. [60]	Midfacial and mandibular fractures	6.0 (3.0–11.9)	95.2 (86.9–98.4)	64.8 (57.6–71.4)	70.0 (39.7–89.2)	35.5 (28.7–43.0)	1.3 (0.3–4.7)	1.0 (0.9–1.1)
Globe position change	Allison et al. [65]	Orbital fractures	17.1 (8.1–32.7)	100.0 (75.7–100.0)	74.5 (60.5–84.7)	100.0 (61.0–100.0)	29.3 (17.6–44.5)	∞	0.8 (0.7–1.0)
Malar eminence flattening	Timashpolsky et al. [61]	Zygoma fractures	72.2 (49.1–87.5)	94.9 (83.1–98.6)	31.6 (21.0–44.5)	86.7 (62.1–96.3)	88.1 (75.0–94.8)	14.1 (3.5–56.0)	0.3 (0.1–0.6)
Nasal assessment									
Epistaxis	Büttner et al. [59]	Midfacial and mandibular fractures	15.6 (13.6–17.8)	95.5 (93.4–96.9)	68.3 (66.0–70.4)	88.1 (82.9–91.9)	34.5 (32.1–36.9)	3.4 (2.3–5.2)	0.9 (0.9–0.9)
	Huang et al. [63]	Midfacial and mandibular fractures	25.0 (19.5–31.4)	99.3 (98.7–99.6)	12.1 (10.6–13.8)	83.3 (72.0–90.7)	90.6 (89.0–91.9)	36.2 (18.7–70.3)	0.8 (0.7–0.8)
	Sitzman et al. [56]	Midfacial and mandibular fractures	31.9 (27.1–37.1)	87.6 (82.2–91.5)	63.2 (59.0–67.3)	81.5 (74.0–87.3)	42.8 (38.0–47.7)	2.6 (1.7–3.9)	0.8 (0.7–0.9)
	Sitzman et al. [60]	Midfacial and mandibular fractures	29.3 (21.8–38.2)	77.8 (66.1–86.3)	64.8 (57.6–71.4)	70.8 (56.8–81.8)	37.4 (29.6–45.9)	1.3 (0.8–2.3)	0.9 (0.8–1.1)
	Smith et el. [58]	Midfacial fractures	22.9 (15.2–33.0)	96.4 (89.9–98.8)	50.0 (42.5–57.5)	86.4 (66.7–95.3)	55.6 (47.4–63.4)	6.3 (1.9–20.6)	0.8 (0.7–0.9)
	Yadav et al. [57]	Orbital fractures	22.5 (18.5–27.1)	86.5 (84.9–88.0)	15.9 (14.5–17.5)	24.0 (19.7–28.8)	85.5 (83.9–87.0)	1.7 (1.3–2.1)	0.9 (0.8–0.9)
Ocular assessment									
Subconjunctival hemorrhage	Allison et al. [65]	Orbital fractures	45.7 (30.5–61.8)	91.7 (64.6–98.5)	74.5 (60.5–84.7)	94.1 (73.0–99.0)	36.7 (21.9–54.5)	5.5 (0.8–37.1)	0.6 (0.4–0.8)
	Büttner et al. [59]	Midfacial and mandibular fractures	16.3 (14.3–18.6)	90.4 (87.6–92.6)	68.3 (66.0–70.4)	78.6 (72.9–83.3)	33.4 (31.1–35.9)	1.7 (1.3–2.3)	0.9 (0.9–1.0)
	Holmgren et al. [54]	Midfacial and mandibular fractures	8.8 (6.6–11.7)	96.0 (93.1–97.7)	61.4 (57.9–64.7)	77.8 (65.1–86.8)	39.8 (36.3–43.4)	2.2 (1.2–4.1)	0.9 (0.9–1.0)
	Sitzman et al. [56]	Midfacial and mandibular fractures	31.3 (26.6–36.5)	86.5 (81.0–90.6)	63.2 (59.0–67.3)	80.0 (72.3–86.0)	42.3 (37.5–47.2)	2.3 (1.6–3.4)	0.8 (0.7–0.9)
	Sitzman et al. [60]	Midfacial and mandibular fractures	33.6 (25.7–42.6)	88.9 (78.8–94.5)	64.8 (57.6–71.4)	84.8 (71.8–92.4)	42.1 (34.1–50.6)	3.0 (1.4–6.4)	0.7 (0.6–0.9)
	Timashpolsky et al. [61]	Orbital floor fractures	76.0 (56.6–88.5)	90.6 (75.8–96.8)	43.9 (31.8–56.7)	86.4 (66.7–95.3)	82.9 (67.3–91.9)	8.1 (2.7–24.3)	0.3 (0.1–0.5)
	Timashpolsky et al. [61]	Zygoma fractures	55.6 (33.7–75.4)	69.2 (53.6–81.4)	31.6 (21.0–44.5)	45.5 (26.9–65.3)	77.1 (61.0–87.9)	1.8 (1.0–3.4)	0.6 (0.4–1.1)
	Yadav et al. [57]	Orbital fractures	31.4 (26.8–36.4)	87.3 (85.8–88.7)	15.9 (14.5–17.5)	31.9 (27.3–36.9)	87.1 (85.5–88.5)	2.5 (2.0–3.0)	0.8 (0.7–0.8)
Hyphema	Yadav et al. [57]	Orbital fractures	4.7 (3.0–7.4)	97.6 (96.8–98.2)	15.9 (14.5–17.5)	27.0 (17.6–39.0)	84.4 (82.8–85.9)	2.0 (1.1–3.4)	1.0 (1.0–1.0)
Diplopia	Allison et al. [65]	Orbital fractures	42.9 (28.0–59.1)	83.3 (55.2–95.3)	74.5 (60.5–84.7)	88.2 (65.7–96.7)	33.3 (19.2–51.2)	2.6 (0.7–9.6)	0.7 (0.5–1.0)
	Büttner et al. [59]	Midfacial and mandibular fractures	15.0 (13.1–17.2)	98.3 (96.8–99.1)	68.3 (66.0–70.4)	95.0 (90.8–97.4)	35.0 (32.6–37.4)	8.9 (4.6–17.2)	0.9 (0.8–0.9)
	Scolozzi et al. [62]	Orbital fractures	39.9 (36.4–43.6)	84.8 (79.4–89.0)	76.9 (74.0–79.5)	89.7 (85.9–92.6)	29.8 (26.3–33.6)	2.6 (1.9–3.7)	0.7 (0.7–0.8)
	Sitzman et al. [56]	Midfacial and mandibular fractures	7.2 (4.9–10.5)	95.9 (92.0–97.9)	63.2 (59.0–67.3)	75.0 (57.9–86.7)	37.5 (33.4–41.9)	1.7 (0.8–3.8)	1.0 (0.9–1.0)
	Sitzman et al. [60]	Midfacial and mandibular fractures	4.3 (1.9–9.7)	100.0 (94.3–100.0)	64.8 (57.6–71.4)	100.0 (56.6–100.0)	36.2 (29.4–43.6)	∞	1.0 (0.9–1.0)
	Yadav et al. [57]	Orbital fractures	3.3 (1.9–5.7)	98.5 (97.8–98.9)	15.9 (14.5–17.5)	29.3 (17.6–44.5)	84.3 (82.8–85.8)	2.2 (1.1–4.2)	1.0 (1.0–1.0)
Extra-ocular movement limitation	Allison et al. [65]	Orbital fractures	25.7 (14.2–42.1)	83.3 (55.2–95.3)	74.5 (60.5–84.7)	81.8 (52.3–94.9)	27.8 (15.8–44.0)	1.5 (0.4–6.2)	0.9 (0.6–1.2)
	Sitzman et al. [56]	Midfacial and mandibular fractures	14.2 (10.8–18.3)	96.4 (92.7–98.2)	63.2 (59.0–67.3)	87.0 (75.6–93.6)	39.5 (35.2–44.0)	3.9 (1.8–8.5)	0.9 (0.8–0.9)
	Sitzman et al. [60]	Midfacial and mandibular fractures	12.1 (7.3–19.2)	98.4 (91.5–99.7)	64.8 (57.6–71.4)	93.3 (70.2–98.8)	37.8 (30.7–45.4)	7.6 (1.0–56.5)	0.9 (0.8–1.0)
	Yadav et al. [57]	Orbital fractures	11.7 (8.7–15.4)	95.9 (94.9–96.7)	15.9 (14.5–17.5)	35.0 (27.1–43.9)	85.2 (83.6–86.6)	2.8 (2.0–4.1)	0.9 (0.9–1.0)
Extra-ocular movement pain	Yadav et al. [57]	Orbital fractures	21.4 (17.5–25.9)	92.1 (90.8–93.2)	15.9 (14.5–17.5)	33.9 (28.1–40.3)	86.1 (84.5–87.5)	2.7 (2.1–3.5)	0.9 (0.8–0.9)
Visual acuity change	Allison et al. [65]	Orbital fractures	20.0 (10.0–35.9)	100.0 (75.7–100.0)	74.5 (60.5–84.7)	100.0 (64.6–100.0)	30.0 (18.1–45.4)	∞	0.8 (0.7–0.9)
	Sitzman et al. [56]	Midfacial and mandibular fractures	9.9 (7.2–13.6)	93.8 (89.4–96.4)	63.2 (59.0–67.3)	73.3 (59.0–84.0)	37.7 (33.5–42.1)	1.6 (0.8–3.0)	1.0 (0.9–1.0)
	Sitzman et al. [60]	Midfacial and mandibular fractures	7.8 (4.1–14.1)	96.8 (89.1–99.1)	64.8 (57.6–71.4)	81.8 (52.3–94.9)	36.3 (29.4–43.8)	2.4 (0.5–11.0)	1.0 (0.9–1.0)
Intra-oral assessment									
Laceration, intra-oral	Holmgren et al. [54]	Midfacial and mandibular fractures	28.5 (24.6–32.7)	90.3 (86.5–93.2)	61.4 (57.9–64.7)	82.4 (75.9–87.5)	44.3 (40.4–48.2)	2.9 (2.0–4.3)	0.8 (0.7–0.8)
	Sitzman et al. [56]	Midfacial and mandibular fractures	17.2 (13.5–21.6)	92.7 (88.2–95.6)	63.2 (59.0–67.3)	80.3 (69.6–87.9)	39.4 (35.0–44.0)	2.4 (1.4–4.1)	0.9 (0.8–1.0)
	Sitzman et al. [60]	Midfacial and mandibular fractures	19.0 (12.9–27.0)	95.2 (86.9–98.4)	64.8 (57.6–71.4)	88.0 (70.0–95.8)	39.0 (31.6–46.8)	4.0 (1.2–12.8)	0.9 (0.8–0.9)
Tooth avulsion	Harrington et al. [64]	Midfacial and mandibular fractures	11.1 (6.3–18.8)	100.0 (94.7–100.0)	59.3 (51.7–66.4)	100.0 (74.1–100.0)	43.6 (36.1–51.4)	∞	0.9 (0.8–1.0)
	Huang et al. [63]	Midfacial and mandibular fractures	4.5 (2.4–8.3)	99.2 (98.6–99.5)	12.1 (10.6–13.8)	42.9 (24.5–63.5)	88.3 (86.6–89.7)	5.4 (2.3–12.7)	1.0 (0.9–1.0)
	Sitzman et al. [56]	Midfacial and mandibular fractures	16.6 (13.0–20.9)	97.9 (94.8–99.2)	63.2 (59.0–67.3)	93.2 (83.8–97.3)	40.6 (36.2–45.1)	8.0 (2.9–21.7)	0.9 (0.8–0.9)
	Sitzman et al. [60]	Midfacial and mandibular fractures	11.2 (6.7–18.2)	96.8 (89.1–99.1)	64.8 (57.6–71.4)	86.7 (62.1–96.3)	37.2 (30.2–44.8)	3.5 (0.8–15.2)	0.9 (0.8–1.0)
Malocclusion	Harrington et al. [64]	Midfacial and mandibular fractures	8.1 (4.2–15.1)	100.0 (94.7–100.0)	59.3 (51.7–66.4)	100.0 (67.6–100.0)	42.8 (35.3–50.5)	∞	0.9 (0.9–1.0)
	Sitzman et al. [56]	Midfacial and mandibular fractures	26.2 (21.8–31.2)	92.7 (88.2–95.6)	63.2 (59.0–67.3)	86.1 (78.1–91.6)	42.2 (37.6–47.0)	3.6 (2.1–6.2)	0.8 (0.7–0.9)
	Sitzman et al. [60]	Midfacial and mandibular fractures	19.0 (12.9–27.0)	95.2 (86.9–98.4)	64.8 (57.6–71.4)	88.0 (70.0–95.8)	39.0 (31.6–46.8)	4.0 (1.2–12.8)	0.9 (0.8–0.9)
Functional and palpation assessment									
Facial pain	Sitzman et al. [56]	Midfacial and mandibular fractures	35.2 (30.3–40.5)	68.9 (62.1–75.0)	63.2 (59.0–67.3)	66.1 (58.9–72.7)	38.2 (33.3–43.4)	1.1 (0.9–1.5)	0.9 (0.8–1.1)
	Sitzman et al. [60]	Midfacial and mandibular fractures	44.8 (36.1–53.9)	69.8 (57.6–79.8)	64.8 (57.6–71.4)	73.2 (61.9–82.1)	40.7 (31.9–50.2)	1.5 (1.0–2.3)	0.8 (0.6–1.0)
Infra-orbital nerve paresthesia	Allison et al. [65]	Orbital fractures	25.7 (14.2–42.1)	91.7 (64.6–98.5)	74.5 (60.5–84.7)	90.0 (59.6–98.2)	29.7 (17.5–45.8)	3.1 (0.4–21.9)	0.8 (0.6–1.1)
	Büttner et al. [59]	Midfacial and mandibular fractures	22.2 (19.9–24.7)	96.4 (94.5–97.7)	68.3 (66.0–70.4)	93.0 (89.4–95.5)	36.6 (34.1–39.1)	6.2 (3.9–9.8)	0.8 (0.8–0.8)
	Scolozzi et al. [62]	Orbital fractures	31.1 (27.8–34.6)	91.0 (86.4–94.2)	76.9 (74.0–79.5)	92.0 (87.8–94.8)	28.4 (25.2–32.0)	3.5 (2.2–5.4)	0.8 (0.7–0.8)
	Sitzman et al. [56]	Midfacial and mandibular fractures	0.3 (0.1–1.7)	100.0 (98.0–100.0)	63.2 (59.0–67.3)	100.0 (20.7–100.0)	36.8 (32.8–41.0)	∞	1.0 (1.0–1.0)
	Sitzman et al. [60]	Midfacial and mandibular fractures	5.2 (2.4–10.8)	98.4 (91.5–99.7)	64.8 (57.6–71.4)	85.7 (48.7–97.4)	36.0 (29.2–43.5)	3.3 (0.4–26.5)	1.0 (0.9–1.0)
	Timashpolsky et al. [61]	Orbital floor fractures	36.0 (20.2–55.5)	90.6 (75.8–96.8)	43.9 (31.8–56.7)	75.0 (46.8–91.1)	64.4 (49.8–76.8)	3.8 (1.2–12.7)	0.7 (0.5–1.0)
	Timashpolsky et al. [61]	Zygoma fractures	38.9 (20.3–61.4)	87.2 (73.3–94.4)	31.6 (21.0–44.5)	58.3 (32.0–80.7)	75.6 (61.3–85.8)	3.0 (1.1–8.3)	0.7 (0.5–1.0)
	Yadav et al. [57]	Orbital fractures	5.6 (3.6–8.4)	97.5 (96.7–98.1)	15.9 (14.5–17.5)	29.9 (20.2–41.7)	84.5 (82.9–86.0)	2.2 (1.3–3.7)	1.0 (0.9–1.0)
Subcutaneous emphysema	Büttner et al. [59]	Midfacial and mandibular fractures	10.7 (9.0–12.6)	99.6 (98.6–99.9)	68.3 (66.0–70.4)	98.4 (94.3–99.6)	34.1 (31.8–36.5)	28.4 (7.0–114.3)	0.9 (0.9–0.9)
	Scolozzi et al. [62]	Orbital fractures	25.4 (22.3–28.7)	94.8 (90.9–97.1)	76.9 (74.0–79.5)	94.2 (89.9–96.7)	27.7 (24.5–31.0)	4.9 (2.7–8.8)	0.8 (0.7–0.8)
Tenderness on palpation	Timashpolsky et al. [61]	Nasal bone fracture	87.5 (52.9–97.8)	89.8 (78.2–95.6)	14.0 (7.3–25.3)	58.3 (32.0–80.7)	97.8 (88.4–99.6)	8.6 (3.6–20.5)	0.1 (0.0–0.9)
	Yadav et al. [57]	Orbital fractures	72.8 (68.0–77.1)	48.7 (46.5–51.0)	15.9 (14.5–17.5)	21.2 (19.0–23.5)	90.4 (88.5–92.1)	1.4 (1.3–1.5)	0.6 (0.5–0.7)
Palpable step-off	Büttner et al. [59]	Midfacial and mandibular fractures	18.5 (16.4–20.9)	99.8 (98.9–100.0)	68.3 (66.0–70.4)	99.5 (97.4–99.9)	36.3 (33.9–38.8)	98.6 (13.9–701.3)	0.8 (0.8–0.8)
	Harrington et al. [64]	Midfacial and mandibular fractures	5.1 (2.2–11.3)	100.0 (94.7–100.0)	59.3 (51.7–66.4)	100.0 (56.6–100.0)	42.0 (34.6–49.7)	∞	0.9 (0.9–1.0)
	Sitzman et al. [56]	Midfacial and mandibular fractures	41.9 (36.7–47.2)	89.6 (84.5–93.2)	63.2 (59.0–67.3)	87.4 (81.4–91.7)	47.3 (42.2–52.4)	4.0 (2.6–6.2)	0.6 (0.6–0.7)
	Sitzman et al. [60]	Midfacial and mandibular fractures	32.8 (24.9–41.7)	93.7 (84.8–97.5)	64.8 (57.6–71.4)	90.5 (77.9–96.2)	43.1 (35.1–51.4)	5.2 (1.9–13.8)	0.7 (0.6–0.8)
	Yadav et al. [57]	Orbital fractures	7.8 (5.4–11.0)	94.7 (93.6–95.7)	15.9 (14.5–17.5)	21.9 (15.6–29.8)	84.4 (82.8–85.9)	1.5 (1.0–2.2)	1.0 (0.9–1.0)
Trismus	Timashpolsky et al. [61]	Zygoma fractures	38.9 (20.3–61.4)	94.9 (83.1–98.6)	31.6 (21.0–44.5)	77.8 (45.3–93.7)	77.1 (63.5–86.7)	7.6 (1.7–32.9)	0.6 (0.4–0.9)
	Yadav et al. [57]	Orbital fractures	3.6 (2.1–6.1)	95.5 (94.5–96.4)	15.9 (14.5–17.5)	13.3 (7.9–21.4)	84.0 (82.4–85.5)	0.8 (0.5–1.4)	1.0 (1.0–1.0)
Mandible locked open	Yadav et al. [57]	Orbital fractures	0.8 (0.3–2.4)	98.6 (97.9–99.0)	15.9 (14.5–17.5)	10.0 (3.5–25.6)	84.0 (82.4–85.5)	0.6 (0.2–1.9)	1.0 (1.0–1.0)
Open fracture	Sitzman et al. [56]	Midfacial and mandibular fractures	6.3 (4.2–9.5)	98.4 (95.5–99.5)	63.2 (59.0–67.3)	87.5 (69.0–95.7)	37.9 (33.8–42.2)	4.1 (1.2–13.5)	1.0 (0.9–1.0)
	Sitzman et al. [60]	Midfacial and mandibular fractures	6.9 (3.5–13.0)	100.0 (94.3–100.0)	64.8 (57.6–71.4)	100.0 (67.6–100.0)	36.8 (30.0–44.3)	∞	0.9 (0.9–1.0)

*Sens.* sensitivity; *Spec.* specificity; *Pre-test prob.* pre-test probability; *PPV* positive predictive value; *NPV* negative predictive value; *LR* + positive likelihood ratio; *LR-* negative likelihood ratio; *CI* confidence interval; ∞ infinite

### Findings related to visual appearance

A total of 24 distinct physical examination findings were identified as being related to the visual appearance of the patient and reported 52 times in the included studies [54–58, 60–65]. The outcomes of the findings were any midfacial or mandibular fracture (*n* = 40), any midfacial fracture (*n* = 2), any orbital fracture (*n* = 7), orbital floor fracture (*n* = 1), and zygoma fracture (*n* = 2). The identified findings included swelling, hematoma, laceration, asymmetry, globe position change, and malar eminence flattening. Regarding swelling, hematoma, and laceration, the diagnostic accuracy was also reported for specific regions of the midfacial skin. For swelling, this included that diagnostic accuracy was also reported for specifically the periorbital region [56, 57, 60, 64]. The region specific findings for hematoma included the forehead [56, 60], peri-orbital region [54, 56–58, 60, 62, 65], eyelid [54, 55], nasal region [56, 60], malar region [56, 60], and the facial or scalp region [54]. For laceration, region specific findings included the forehead [56, 58, 60, 63], peri-orbital region [56, 57, 60], eyebrow [54], eyelid [54], conjunctiva [54], nasal region [54, 56, 60], malar region [56, 60], peri-oral region [56, 60], and the lip [54]. Among the physical examination findings related to swelling, hematoma and laceration, high pooled specificity was found for eyelid hematoma, eyebrow laceration, conjunctival laceration, nasal laceration, and malar laceration ranging from 0.19 to 0.98 (Table 3 & Fig. 3a). The diagnostic odds ratio for these physical examination findings ranged from 1.10 to 3.48. Regarding asymmetry, globe position change, and malar eminence flattening, the specificity, PPV, and LR + were found to be high.

### Findings related to nasal assessment

Epistaxis was the only reported physical examination finding related to the nasal assessment and was reported in 6 studies [56–60, 63]. The outcomes included any midfacial or mandibular fracture (*n* = 4), any midfacial fracture (*n* = 1), and any orbital fracture (*n* = 1). The pooled specificity was found to be high (0.94) and the pooled sensitivity remained low (0.25). The diagnostic odds ratio was 5.43 (Table 3 & Fig. 3b).

### Findings related to ocular assessment

A total of 6 distinct physical examination findings were identified in relation to the ocular assessment and reported 23 times in the included studies [54, 56, 57, 59–62, 65]. The outcomes were any midfacial or mandibular fracture (*n* = 11), any orbital fracture (*n* = 10), orbital floor fracture (*n* = 1), and zygoma fracture (*n* = 1). The identified findings included subconjunctival hemorrhage [54, 56, 57, 59–61, 65], hyphema [57], diplopia [56, 57, 59, 60, 62, 65], extra-ocular movement limitation [56, 57, 60, 65], extra-ocular movement pain [57], and visual acuity change [56, 60, 65]. The pooled specificity of all the physical examination findings was high, ranging from 0.89 to 0.94, and the pooled sensitivity was low, ranging from 0.09 to 0.36 (Table 3 & Fig. 3c). The diagnostic odds ratio ranged from 1.79 to 3.27. Although the outcomes varied, most of the studies reported a high PPV and LR + for the findings related to the ocular assessment, with two individual studies reporting a PPV of 100 and infinite LR + for diplopia and visual acuity change [60, 65].

### Findings related to the intra-oral assessment

A total of 3 distinct physical examination findings were identified to be related to the intra-oral assessment and reported in 10 times of the included studies [54, 56, 60, 63, 64]. All of these reported physical examination findings were studied using any midfacial or mandibular fracture as outcome (*n* = 10). Identified findings included malocclusion [56, 60, 64], intra-oral laceration [54, 56, 60], and tooth avulsion [56, 60, 63, 64]. The pooled specificity was high, ranging from 0.92 to 0.98, and the sensitivity was low for all findings, ranging from 0.10 to 0.21 (Table 3 & Fig. 3d). The diagnostic odds ratio ranged from 3.41 to 6.64. The PPV found higher than 80.0 in almost all of the studies, with one study reporting a PPV of 100 and an infinite LR + for malocclusion and tooth avulsion [64]. The NPV was low in all studies.

### Findings related to functional assessment and palpation of the midface

Regarding findings related to the functional assessment and palpation of the midface, a total of 8 distinct physical examination were identified that were reported 24 times in the included studies [56, 57, 59–62, 64, 65]. The outcomes were any midfacial or mandibular fracture (*n* = 12), any orbital fracture (*n* = 8), orbital floor fracture (*n* = 1), nasal bone fracture (*n* = 1), and zygoma fracture (*n* = 2). The identified findings included facial pain [56, 60], infra-orbital nerve paresthesia [56, 57, 59–62, 65], subcutaneous emphysema [59, 62], tenderness on palpation [57, 61], palpable step-off [56, 57, 59, 60, 64], trismus [57, 61], mandible locked open [57], and open fracture [56, 60]. The pooled specificity was high for infra-orbital nerve paresthesia, subcutaneous emphysema, palpable step-off, trismus, mandible locked open, and open fracture, ranging from 0.69 to 0.99. The pooled sensitivity remained low for the findings, ranging from 0.04 to 0.39 (Table 3 & Fig. 3e). The diagnostic odds ratio ranged from 1.39 to 11.38. A high PPV and LR + was found for infra-orbital nerve paresthesia, subcutaneous emphysema, palpable step-off and open fracture. Individual studies reported a PPV of 100 and a corresponding infinite LR + for infra-orbital nerve paraesthesia, palpable step-off and open fracture [56, 60, 64]. A high NPV was found for tenderness on palpation. The NPV of the other physical examination findings was low.

### Publication bias

The Deek’s funnel plot tests showed that publication bias was significant for subconjunctival hemorrhage with midfacial and mandibular fractures (Supplementary material S4). The statistical significance of the publication bias could not be assessed for 15 physical examination findings because only two studies provided data.

### Clinical decision aids

Clinical decision aids were reported in 8 studies (Table 4). Four studies assessed the Wisconsin criteria [56, 60, 61, 64]. The criteria were defined as any presence of a bony step-off or instability, malocclusion, tooth absence, peri-orbital swelling or contusion, and a Glasgow coma score of less than 14, using any midfacial or mandibular fracture as an outcome [56]. The sensitivity of these criteria ranged from 80.2 to 98.2%, and the specificity ranged from 22.3 to 41.2%. Clinical decision aids specifically for orbital fractures were presented in 2 studies [55, 65]. One study focused on the need for a facial CT for head injury patients [55], and constructed a clinical decision aid that produced a sensitivity of 55.1% and a specificity of 100.0% in the presence of either blepharohematoma in one or two orbits, palpable fracture line, infra-orbital nerve hypesthesia, ocular motility disturbance, skin emphysema, enophthalmos or exophthalmos, impaired pupil reaction, and decrease in vision. Another study focused on the identification of head injury patients who had benefitted from including the orbits in the head CT [65]. Another clinical decision aid was constructed based on unbounded subconjunctival hemorrhage, reduced sensation in the distribution of the infra-orbital nerve, change in the position of the globe, reduced visual acuity or any two of the following, peri-orbital bruising, diplopia, and limited eye movement. The presence of any of these findings produced a sensitivity of 80.0% and specificity of 75.0%. Two studies produced a clinical decision aid for orbital fractures using a risk score [57, 62]. In one study, the risk score consisted of assigning a point for orbital rim tenderness, peri-orbital emphysema, subconjunctival hemorrhage, impaired extra-ocular movement, painful extra-ocular movement and epistaxis [57]. The other study assigned one point for male sex, etiology other than assault, peri-orbital ecchymosis, peri-orbital emphysema, infra-orbital nerve hypoesthesia and diplopia. One study introduced clinical decision aids, which were referred to as the Stony Brook University Hospital (SBUH) criteria, for orbital floor fractures, zygoma fractures and nasal fractures [61]. The respective sensitivities and specificities were 92.0% and 75.0% for orbital floor fractures, 88.9% and 51.3% for zygoma fractures, and 87.5% and 87.8% for nasal fractures. Contingency tables of the physical examination findings and clinical decision aids are presented in Supplementary Material S5.

**Table 4:** Reported clinical decision aids

Author	Year	Clinical decision aid	Sens. (95% CI)	Spec. (95% CI)	PPV (95% CI)	NPV (95% CI)	LR + (95% CI)	LR- (95% CI)
Exadaktylos et al. [55]	2005	Orbital fracture decision tool for no symptoms	15.3 (9.9–22.8)	5.2 (3.5–7.5)	3.8 (2.4–5.9)	20.0 (13.9–27.9)	0.2 (0.1–0.2)	16.3 (11.1–24.1)
Exadaktylos et al. [55]	2005	Orbital fracture decision tool for blepharohematoma only	29.7 (22.2–38.4)	94.8 (92.5–96.5)	58.3 (45.7–69.9)	84.6 (81.3–87.4)	5.7 (3.6–9.2)	0.7 (0.7–0.8)
Exadaktylos et al. [55]	2005	Orbital fracture decision tool for any symptoms	55.1 (46.1–63.8)	100.0 (99.2–100.0)	100.0 (94.4–100.0)	90.1 (87.3–92.3)	∞	0.4 (0.4–0.5)
Sitzman et al. [56]	2011	Wisconsin criteria	98.2 (96.1–99.2)	22.3 (17.0–28.7)	68.5 (64.2–72.5)	87.8 (75.8–94.3)	1.3 (1.2–1.4)	0.1 (0.0–0.2)
Sitzman et al. [60]	2015	Wisconsin criteria	97.4 (92.7–99.1)	20.6 (12.5–32.2)	69.3 (61.9–75.9)	81.3 (57.0–93.4)	1.2 (1.1–1.4)	0.1 (0.0–0.4)
Timashpolksy et al. [61]	2016	Wisconsin criteria	89.8 (78.2–95.6)	40.0 (11.8–76.9)	93.6 (82.8–97.8)	28.6 (8.2–64.1)	1.5 (0.7–3.1)	0.3 (0.1–1.0)
Harrington et al. [64]	2018	Wisconsin criteria	80.8 (72.0–87.4)	41.2 (30.3–53.0)	66.7 (57.8–74.5)	59.6 (45.3–72.4)	1.4 (1.1–1.7)	0.5 (0.3–0.8)
Yadav et al. [57]	2012	Orbital fracture risk score 0	88.5 (85.0–92.0)	32.5 (30.3–34.7)	-	93.7 (91.8–95.7)	-	0.4 (0.3–0.5)
		Orbital fracture risk score 1	55.3 (49.7–61.0)	77.1 (75.0–79.1)	-	90.1 (88.6–91.6)	-	0.6 (0.5–0.7)
		Orbital fracture risk score 2	25.8 (21.0–30.7)	93.8 (92.6–94.9)	-	87.0 (85.5–88.5)	-	0.8 (0.7–0.8)
		Orbital fracture risk score 3	8.2 (5.3–11.2)	98.9 (98.4–99.4)	-	85.1 (83.6–86.5)	-	0.9 (0.9–1.0)
		Orbital fracture risk score 4	1.9 (0.2–3.5)	99.7 (99.5–100.0)	-	84.3 (82.8–85.8)	-	1.0 (1.0–1.0)
		Orbital fracture risk score 5–6	0.2 (0.0–0.7)	99.9 (99.7–100.0)	-	84.1 (82.6–85.6)	-	1.0 (1.0–1.0)
Scolozzi et al. [62]	2017	Orbital fracture score > 1	97.3 (95.8–98.3)	6.2 (3.6–10.3)	77.5 (74.6–80.1)	40.6 (25.5–57.7)	1.0 (1.0–1.1)	0.4 (0.2–0.9)
		Orbital fracture score > 2	76.7 (73.5–79.7)	60.2 (53.5–66.6)	86.5 (83.6–89.0)	43.8 (38.2–49.5)	1.9 (1.6–2.3)	0.4 (0.3–0.5)
		Orbital fracture score > 3	40.1 (36.5–43.8)	90.5 (85.8–93.8)	93.4 (90.0–95.7)	31.3 (27.7–35.0)	4.2 (2.8–6.5)	0.7 (0.6–0.7)
		Orbital fracture score > 4	14.7 (12.3–17.5)	97.2 (93.9–98.7)	94.5 (88.5–97.5)	25.5 (22.6–28.7)	5.2 (2.3–11.6)	0.9 (0.8–0.9)
Allison et al. [65]	2019	Orbital fracture decision tool	80.0 (64.1–90.0)	75.0 (46.8–91.1)	90.3 (75.1–96.7)	56.3 (33.2–76.9)	3.2 (1.2–8.6)	0.3 (0.1–0.6)
Timashpolksy et al. [61]	2016	SBUH nose	87.5 (52.9–97.8)	87.8 (75.8–94.3)	53.8 (29.1–76.8)	97.7 (88.2–99.6)	7.1 (3.2–15.8)	0.1 (0.0–0.9)
		SBUH orbital floor	92.0 (75.0–97.8)	75.0 (57.9–86.7)	74.2 (56.8–86.3)	92.3 (75.9–97.9)	3.7 (2.0–6.8)	0.1 (0.0–0.4)
		SBUH zygoma	88.9 (67.2–96.9)	51.3 (36.2–66.1)	45.7 (30.5–61.8)	90.9 (72.2–97.5)	1.8 (1.3–2.6)	0.2 (0.1–0.8)

*Sens.* sensitivity; *Spec.* specificity; *PPV* positive predictive value; *NPV* negative predictive value; *LR* + positive likelihood ratio; *LR-* negative likelihood ratio; *CI* confidence interval; *SBUH* Stony Brook University Hospital

*- Orbital fracture decision tool-* divided into: ‘no symptoms’, ‘blepharohaematoma only’ or ‘any symptoms’ thereof; blepharohaematoma of one or two orbits, palpable fracture line, infra-orbital nerve hypesthesia, ocular motility disturbance, skin emphysema, enophthalmos or exophthalmos, impaired pupil reaction and decrease in vision

*- Wisconsin criteria*- the presence of any of the following findings: bony step-off or instability, malocclusion, tooth absence, peri-orbital swelling or contusion, and a Glasgow coma score of less than 14

*- Orbital fracture risk score (0–6)-* a risk score assigning a point for: orbital rim tenderness, peri-orbital emphysema, subconjunctival hemorrhage, impaired extra-ocular movement, painful extra-ocular movement and epistaxis

*- Orbital fracture score (0–4* +*)-* a risk score assigning a point for: male sex, etiology other than assault, peri-orbital ecchymosis, peri-orbital emphysema, infra-orbital nerve hypoesthesia and diplopia

*- Orbital fracture decision tool-* the presence of any of the following findings: unbounded subconjunctival hemorrhage, reduced sensation in the distribution of the infra-orbital nerve, change in position of the globe, reduced visual acuity or any two from peri-orbital bruising, diplopia and limited eye movement

*- SBUH nose*- presence of the any of the following findings: bony or septal deviation, septal hematoma, tenderness, depression/angulation, ecchymosis or swelling

*- SBUH orbital floor*- the presence of the any of the following findings: subjective diplopia, upgaze limitation, enophthalmos /depression, infra-orbital nerve paresthesia or anaesthesia, subconjunctival hemorrhage, ecchymosis or swelling

*- SBUH zygoma-* presence of the any of the following findings: cheek flatness, subconjuctival haemorrhage, trismus, antimongoloid slant, infra-orbital nerve paraesthesia/anaesthesia, ecchymosis or swelling and palpable step

## Discussion

The assessment of midfacial and mandibular injury is characterized by particular physical examination findings. Understanding the predictive value of each finding may help emergency physicians to deliver a more optimal diagnostic management. In this systematic review and meta-analysis, we synthesized the best available evidence regarding the diagnostic accuracy of the physical examination findings and the accompanying clinical decision aids. The meta-analysis provided evidence of high specificity and low sensitivity for most of the individual physical examination findings related to the visual appearance of the patient; nasal, ocular, and intra-oral assessments; and findings related to the functional assessment and palpation of the midface. This indicates that the absence of any physical examination findings can be used to successfully identify patients who do not have a midfacial fracture, whereas the presence of individual findings does not necessarily mean that patients have a midfacial fracture. Among these physical examination findings, we observed a high diagnostic odds ratio for epistaxis, tooth avulsion, malocclusion, infra-orbital nerve paraesthesia and palpable step-off, indicating that the likelihood of diagnosing a midfacial fracture is high when these findings are present during the physical examination. Also, particular findings had a high PPV and corresponding LR + . From a clinical perspective, emergency department physicians are blinded for the potential presence of a fracture during the physical examination and so these individual findings are especially useful for identifying patients at risk of the presence of a midfacial fracture and radiological imaging should be strongly considered for these patients. The NPV and LR- remained low for almost all the physical examination findings. Hence, the individual findings were unable to identify patients with a low risk of midfacial fractures and who did not require radiological imaging. However, this should be interpreted with caution due the low number of included studies and the high degree of risk of bias and concerns regarding the applicability of most of the studies.

### Clinical decision aids

It is of particular interest how a combination of physical examination findings performs as a clinical decision aid. Accordingly, the studies included in this systematic review proposed a variety of clinical decision aids using any midfacial or mandibular fracture, orbital fracture, orbital floor fracture, nasal fracture, and zygoma fractures as an outcome. The University of Wisconsin produced a clinical decision aid with sufficient diagnostic accuracy for patients suspected of midfacial or mandibular fractures [56]. However, validation of these criteria was unsuccessful in three other studies due to lower diagnostic accuracy outcomes [60, 61, 64]. The other studies focused on clinical decision aids for the identification of specific midfacial fractures, five of which were for orbital fractures [55, 57, 61, 62, 65]. The relevance of specifically studying the latter is emphasized for two reasons. First, orbital fractures are commonly found in patients presenting with a head injury and, therefore, it is often discussed whether the orbits should be included when performing a head CT [7, 55, 63]. Second, orbital fractures are associated with complications, such as entrapment of the extraocular muscles or retrobulbar hemorrhage, that require immediate surgical intervention and should therefore not be missed [15, 66–69]. Three of the five studies successfully produced a clinical decision aid with this focus, whereas the two other produced a score to stratify patients into risk categories for the presence of orbital fractures [57, 62]. One study based the risk score on physical examination findings only [57] whereas the other study also included sex and the mechanism of injury [62]. Although these scores identified the high risk fracture patients, the authors emphasized that further research is needed to determine a weighted cut-off. Nevertheless, patients with a high score were strongly suspected of having orbital fractures. None of these clinical decision aids were validated.

Most importantly, this systematic review did not identify a clinical decision aid that used any midfacial anatomy as an outcome. Yet, both the midface and mandible are known for their characteristic and complex anatomy, consequently each producing region-specific physical examination findings. Hence, we believe that both the midfacial and mandibular region should have a dedicated clinical decision aid, and we suspect that false positive findings might be more likely in studies where any midfacial or mandibular fracture is used as an outcome. For instance, the Wisconsin criteria score was positive for patients suffering peri-orbital hematoma while being diagnosed with a mandibular fracture. Conversely, malocclusion is considered to be a more common finding in mandibular trauma patients due to changes to the temporomandibular joints and the more prominent position of the alveolar process. Dedicating a clinical decision aid to midfacial fractures would allow it to be focused on physical examination findings related to the midfacial region, making it more easily reproducible. This is especially appreciated because a majority of midfacial trauma patients are initially assessed by emergency physicians and trauma surgeons who are not specifically trained to assess these patients.

### Radiological imaging

Our systematic review did not find any studies that used CBCT as a reference test. CBCT scanners are dedicated to the oral and maxillofacial region and datasets are acquired while the system rotates around the patient [22, 33, 70]. A probable explanation is that the system can only be used on patients with isolated midfacial trauma, or patients for whom the initial management did not provide evidence of additional injuries [71]. For that reason, the availability of CBCT scanners in the emergency department is usually limited, and the systems are mostly used in outpatient clinics. A CT, on the other hand, is able to scan multiple body parts resulting in single data acquisition by transporting the patient through the gantry in synchrony with continuous data acquisition [72]. This is especially appreciated for midfacial trauma patients with concomitant cervical spine and head injuries which force the patients into a supine position [7, 73–76]. Nevertheless, both CT and CBCT have the major advantage that they overcome superimposition of structures that inevitably occurs with conventional radiography [22, 30, 32].

### Quality of evidence and bias

In most of the included studies, there was an unclear risk of bias for the domains of the index test, reference standard, and flow and timing. Information regarding either the blinded interpretation of physical examination findings, or the blinded interpretation of CT data, was not reported in these studies. Not blinding the interpretation introduces important biases such as, for example, recording physical examination findings as present more likely if the emergency department workers are aware a priori of fractures being diagnosed on a CT. This type of bias cannot be controlled and therefore was judged as unclear in the studies. High unclear risk of bias was found for the flow and timing domain because no information was provided regarding the interval between the assessment of the physical examination findings and the CT. The accuracy of the interpretation decreases as the interval increases and should therefore be as short as possible. However, it is likely that in an emergency department setting the majority of patients are assessed within hours after the trauma, and a CT is conducted within the same time frame. High applicability concerns were found for the patient selection and reference standard domains. Regarding the selection of patients, a variety of studies focused on head injury patients only who, one would expect, were injured more severely, therefore introducing selection bias and affecting the interpretation of the physical examination findings. Concerns regarding the applicability of the reference standard were due to the use of an outcome other than ‘any midfacial fracture’. Concerns regarding the applicability of the index test were unclear in many studies (i.e., the standardization, handling or interpretation of the physical examination findings). It was especially unclear how the scoring of the chart review was handled by the retrospective studies, and if the data were reported systematically. Not reporting data as an absent physical examination finding could result in bias due to false negative outcomes. Also, the included studies did not report how “not assessable”| physical examination findings were handled, for instance the inability to score ocular related findings in patients with severe peri-orbital swelling.

### Strengths and limitations

The strength of this review is the detailed literature search, eligibility assessment of studies by two independent reviewers, good inter-observer agreement, structured risk of bias assessment using the QUADAS-2 tool, and conducting and reporting analyses according to the Cochrane handbook and PRISMA statement. A major limitation is the interpretation of the pooled outcomes due to the low or unclear quality of the studies, as well as the high concerns regarding applicability. The likely source of this bias was due to the patient selections and the fracture outcomes. Also, most of the studies were single-center trials thereby potentially introducing geographic and demographic biases. Another limitation is that we were unable to perform a meta-regression analysis of the midfacial fracture subgroups due the limited number of studies and data.

### Implications and future research

Future research should focus on the diagnostic accuracy of the physical examination findings using ‘any midfacial fractures’ as an outcome. Particular interest should be paid to the QUADAS-2 domains where high and unclear risk of bias was observed. Studies should include a consecutive population of midfacial trauma patients and inappropriate exclusion, such as multi-trauma patients, should be avoided. A standardized set of physical examination findings should be reproducible and should be assessed before knowing the CT outcome. The interpretation of the CT datasets should be interpreted by either a board certified radiologist or oral and maxillofacial surgeon. Ideally, the study should be conducted as a prospective multi-center trial to avoid geographical bias. Data from a large population of midfacial fracture patients should allow for a regression analysis to study how physical examination findings can predict fracture subtypes, such as orbital or zygomaticomaxillary complex fractures. Above all, the aim of identifying relevant individual findings would be to produce a clinical decision aid to reduce exposure of patients to unnecessary radiological imaging.

### Conclusions

Based on all the currently available evidence, the present systematic review and meta-analysis identified the diagnostic accuracy of individual physical examination findings related to visual appearance, nasal and ocular assessment, intra-oral assessment and functional and palpation assessment of midfacial fractures compared to CT. The high specificity reveals that the absence of physical examination findings can aid in identifying patients who do not have a midfacial fracture, whereas the low sensitivity is evidence that the presence of individual findings cannot be used to accurately identify patients with midfacial fractures. Although, various clinical decision aids and risk scores were presented in the reviewed studies, none focused on the identification of any midfacial fracture. The results herein should be interpreted with caution due the limited number of studies as well as the high risk of bias and concerns regarding the applicability.

## Supplementary Information

Below is the link to the electronic supplementary material.

Supplementary file1 (DOCX 4119 KB)

Supplementary file2 (XLSX 379 KB)
