# Supplementary material for: Diagnostic accuracy of physical examination findings for midfacial fractures: a systematic review and meta-analysis
Source: Clin Oral Investig. 2022 Mar 17;26(4):3405–27. doi: 10.1007/s00784-022-04423-y (PMC8979892; doi:10.1007/s00784-022-04423-y)
Supplement: Supplementary file 1 — Supplementary file1 (DOCX 4119 KB) [file 784_2022_4423_MOESM1_ESM.docx]

**Captions to supplementary material**

**Supplementary material S1**: Search strategy for each electronic database.

**Supplementary material S2**: Description of the QUADAS-2 critical appraisal checklist.

**Supplementary material S3**: Excluded articles with reasons after full-text screening.

**Supplementary material S4**: Deeks funnel plots for the individual physical examination findings. Publication bias was presented plotting the diagnostic odds ratio against the inverse of the square root (sqrt) of effective sample size (ESS).

**Supplementary material S5**: Contingency tables for physical examination findings and clinical decision aids.

| **MEDLINE** |
| --- |
| (("Facial Injuries"[Mesh] OR "Facial Bones"[Mesh] OR maxillofacial*[tiab] OR facial[tiab] OR craniofacial[tiab] OR orofacial[tiab] OR midfacial[tiab] OR frontal sinus[tiab] OR maxillary sinus[tiab] OR paranasal sinus[tiab] OR orbital*[tiab] OR blow-out[tiab] OR blowout[tiab] OR zygoma*[tiab] OR nasal[tiab] OR nose[tiab] OR nasoorbitalethmoid[tiab] OR naso-orbitoethmoid[tiab] OR maxillary[tiab] OR dentoalveolar[tiab] OR dento-alveolar[tiab] OR le fort*[tiab] OR lefort*[tiab]) AND ("Skull Fractures"[Mesh] OR fracture*[tiab]))  **AND**  (“Physical Examination”[Mesh] OR “Clinical Decision-Making”[Mesh] OR “Symptom Assessment”[Mesh] OR "Decision Support Systems, Clinical"[Mesh] OR “Clinical Audit”[Mesh] OR “Signs and Symptoms”[Mesh] OR "Skull Fractures/diagnosis"[Mesh] OR physical examination*[tiab] OR clinical decision*[tiab] OR clinical pattern*[tiab] OR clinical aid[tiab] OR clinical presentation*[tiab] OR clinical criteria[tiab] OR diagnostic criteria[tiab] OR diagnostic performance[tiab] OR decision instrument*[tiab] OR decision aid*[tiab] OR decision rule*[tiab] OR risk score*[tiab] OR clinical judgement*[tiab] OR clinical findings[tiab] OR clinical examination*[tiab] OR physical examination*[tiab] OR clinical diagnos*[tiab] OR clinical assessment*[tiab] OR clinical detect*[tiab] OR clinically assess*[tiab] OR clinically detect*[tiab] OR symptom[tiab] OR screening tool*[tiab] OR symptoms[tiab] OR signs[tiab] OR (type*[tiab] AND injur*[ti]) OR associated injur*[tiab] OR (concomitant[tiab] AND injur*[tiab]) OR (occur*[tiab] AND injur*[ti]))  **AND**  ("Sensitivity and Specificity"[MeSH] OR “ROC Curve”[MeSH] OR "Validation Study" [Publication Type] OR specificit*[tiab] OR sensitiv* [tiab] OR screening[tiab] OR reference value*[tiab] OR false positive*[tiab] OR false negative*[tiab] OR predictive value*[tiab] OR roc[tiab] OR likelyhood*[tiab] OR likelihood*[tiab] OR accura*[tiab] OR predict*[tiab] OR correlat*[tiab] OR diagnostic value*[tiab])  **NOT**  ("Child"[Mesh] NOT ("Adolescent"[Mesh] OR "Adult"[Mesh])) |

**Supplementary material S1**: Search strategy for each electronic database

| **EMBASE** |
| --- |
| (('maxillofacial injury'/exp OR 'facial bone'/exp OR 'orbit'/exp OR 'zygoma'/exp OR 'skull'/de OR 'maxilla'/exp OR 'nasal bone'/exp OR (maxillofacial* OR facial OR craniofacial OR orofacial OR midfacial OR ‘frontal sinus’ OR ‘maxillary sinus’ OR ‘paranasal sinus’ OR orbital* OR ‘blow-out’ OR blowout OR zygoma* OR nasal OR nose OR nasoorbit* OR ‘naso-orbitoeth*’ OR maxillary OR dentoalveolar OR ‘dento-alveolar’ OR ‘le fort*’ OR lefort*):ab,ti) AND ('skull fracture'/exp OR fracture*:ab,ti))  **AND**  (‘physical examination’/exp OR 'clinical examination'/exp OR ‘clinical decision making’/exp OR 'clinical decision support system'/exp OR ‘symptom assessment’/exp OR ‘clinical audit’/exp OR 'injury scale'/exp OR 'preoperative evaluation'/exp OR 'scoring system'/exp OR 'injury severity'/exp OR 'disease association'/exp OR (‘physical examination*’ OR (clinical* NEXT/3 (decision* OR pattern* OR aid* OR presentation* OR criteria OR tool* OR instrument* OR judgement* OR findings OR examination* OR assess* OR diagnos* OR detect*)) OR (diagnostic NEXT/3 (aid* OR performance)) OR (decision NEXT/3 (instrument* OR aid* OR rule* OR tool*)) OR ‘risk score*’ OR ‘physical examination*’ OR symptom* OR signs OR ‘screening tool*’ OR ‘associated injur*’ OR ((type OR concomitant OR occur* OR sever*) NEAR/5 injur*)):ab,ti)  **AND**  (‘sensitivity and specificity’/exp OR 'diagnostic accuracy'/exp OR ‘receiver operating characteristic’/exp OR 'validation study'/exp OR 'correlation analysis'/exp OR 'diagnostic test accuracy study'/exp OR 'predictive value'/exp OR 'prediction'/exp OR 'predictor variable'/exp OR 'reproducibility'/exp OR (specificit* OR sensitiv* OR screening OR “reference value*” OR ‘false positive’ OR ‘false negative’ OR ‘predictive value*’ OR roc OR likelyhood* OR likelihood* OR accura* OR predict* OR correlat* OR ‘diagnostic value*’):ab,ti)  **NOT**  ('child'/exp NOT ('adolescent'/exp OR 'adult'/exp)) |

| **CINAHL** |
| --- |
| ((MH “Facial Injuries+”) OR ((maxillofacial* OR facial OR craniofacial OR orofacial OR midfacial OR “frontal sinus” OR “maxillary sinus” OR “paranasal sinus” OR orbita* OR “blow-out” OR blowout OR zygoma* OR nasal OR nose OR nasoorbitalethmoid OR “naso-orbit*” OR maxillary OR dentoalveolar OR “dento-alveolar” OR “le fort*” OR lefort*) AND ((MH "Fractures+") OR TI (injur* OR trauma*) OR fracture*)))  **AND**  ((MH “Physical Examination”) OR (MH “Decision Making, Clinical”) OR physical examination* OR  OR (clinical* N3 (decision* OR pattern* OR aid* OR presentation* OR criteria OR tool* OR instrument* OR judgement* OR findings OR examination* OR assess* OR diagnos* OR detect* OR score OR scoring)) OR “diagnostic performance” OR ((decisi* OR diagnostic) N3 (instrument* OR aid* OR rule* OR tool* OR score OR scoring)) OR “risk score” OR “screening tool*” OR “physican examination” OR symptom* OR sign OR signs OR ((type OR concomitant OR occur* OR sever* OR associat*) N5 injur*))  **AND**  ((MH “Sensitivity and Specificity”) OR (MH “ROC Curve”) OR specificit* OR sensitiv* OR accura* OR “reference value*” OR “false positive” OR “false negative” OR “predictive value*” OR “diagnostic value” OR roc OR likelyhood* OR likelihood* OR accura* OR predict* OR correlat*) |

| **Cochrane Controlled Trial Register** |
| --- |
| (“maxillofacial” OR “facial” OR “craniofacial” OR “orofacial” OR “midfacial” OR “frontal sinus” OR “maxillary sinus” OR “paranasal sinus” OR orbita* OR “blow-out” OR “blowout” OR “zygoma” OR “nasal” OR “nose” OR “nasoorbitalethmoid” OR “naso-orbitoethmoid” OR “maxillary” OR “dentoalveolar” OR “dento-alveolar” OR “le fort” OR “lefort”) AND (injur* OR trauma* OR fracture*)  **AND**  ("specificity” OR “sensitivity” OR “screening” OR “accuracy” OR “reference value” OR “false positive” OR “false negative” OR predict* OR “roc” OR “receiver operating characteristic” OR “likelyhood” OR likelihood” OR correlat* OR diagnos* OR reproducibilit*) |

**Supplementary material S2**: Description of the QUADAS-2 critical appraisal checklist

**Domain 1: Patient selection**

***Risk of bias: Was the patient selection process biased?***

- Signalling question 1: Was a consecutive or random sample of patients enrolled?

A *“****Yes****”* was scored if a consecutive or random sample of eligible patients was enrolled; *“****No****”* if patients were selected by convenience; and *“****Unclear****”* if the study did not report how the sample of patients was enrolled.

- Signalling question 2: Was a case-control design avoided?

A “***Yes***” was scored if a case-control design was avoided; “***No***” if the study was conducted using a case-control design; and “***Unclear***” if insufficient information was reported regarding the study design.

- Signalling question 3: Did the study avoid inappropriate exclusions?

A “***Yes***” was scored if the study avoided inappropriate exclusions; “***No***” if patients were excluded inappropriately (e.g., exclusion of polytrauma patients, patients mildly suspected of a midfacial fracture, patients with altered states of consciousness); “***Unclear***” was scored if insufficient information was provided to state whether inappropriate exclusions were avoided.

We considered the bias to be “***Low Risk***” if we scored “***Yes***” for all of the three signalling questions; “***High Risk***” if we scored “***No****”* for any of the signalling questions; and “***Unclear***” if we scored “***Unclear***” for any of the signalling questions and the other signalling questions were answered as “***Yes***”.

***Concerns regarding applicability: Is there concern that the included patients do not match the review question?***

We assessed whether the studied patient population matched the review question, and were treated for midfacial trauma as a primary condition. We judged the included patients as “***Low Applicability Concern***” if they matched the review question; “***High Applicability Concern***” if the included patients did not match the review question (e.g., a midfacial trauma was not the primary condition for selecting the patient population, but another condition such as traumatic brain injury); “***Unclear Applicability Concern***” if insufficient information was provided to assess if the included patients matched the review question.

**Domain 2: Index test**

***Risk of bias: Could the conduct or interpretation of the test have introduced bias?***

- Signalling question 1: Were the physical examination findings tested without knowledge of the outcome of a CT or CBCT?

A “***Yes***” was scored if the physical examination findings were tested without knowledge of the CT or CBCT outcome; “***No***” if the physical examination findings were tested with knowledge of the CT or CBCT outcome; and “***Unclear***” if no information was given to assess whether the physical examination findings were tested without any knowledge of the CT or CBCT outcome.

- Signalling question 2: Were all physical examination findings reproducible or was a clear definition provided?

A “***Yes***” was scored if the physical examination findings were reproducible and clearly defined; “***No***” if the physical examination findings were not reproducible or no clear definition was provided; “***Unclear***” if insufficient information was provided to assess whether the physical examination findings were reproducible or clearly defined.

We considered the bias to be “***Low Risk***” if we scored “***Yes***” for both signalling questions; “***High Risk***” if we scored “***No***” for any of the questions; and “***Unclear***” if we scored “***Unclear***” for either of the signalling questions and the other signalling question resulted in a “***Yes***”.

***Concerns regarding applicability: Is there concern that the physical examination findings, their standardization, conduct, or interpretation differ from the review question?***

We judged “***Low Applicability Concern***” if there were no concerns regarding the standardization, conduct or interpretation of the physical examination findings; “***High Applicability Concern***” if there were concerns regarding the standardization, conduct or interpretation of the physical examination findings (e.g., the inability to control the standardization in the retrospective study designs); “***Unclear Applicability Concern***” if insufficient information was provided to assess the standardization, handling or interpretation of the physical examination findings.

**Domain 3. Reference test**

***Risk of bias: Could the conduct or interpretation of the test have introduced bias?***

- Signalling question 1: Were the CT or CBCT datasets assessed by a consultant or board certified radiologists, oral and maxillofacial surgeons or medical specialist?

A “***Yes***” was scored if the CT or CBCT datasets were interpreted by a consultant or board certified radiologists, oral and maxillofacial surgeons or medical specialist; “***No***” if the CT or CBCT datasets were not interpreted by a consultant or board certified radiologists, oral and maxillofacial surgeons or medical specialist; “***Unclear***” if insufficient information was provided to assess whether a consultant or board certified radiologists, oral and maxillofacial surgeons or medical specialist had interpreted the datasets.

- Signalling question 2: Were the CT or CBCT datasets assessed without any knowledge of the findings of the physical examination of the midface.

A “***Yes***” was scored if the CT or CBCT was interpreted without any knowledge of the physical examination findings; “***No***” if the CT or CBCT was interpreted with knowledge about the physical examination findings; “***Unclear***” if insufficient information was provided regarding the blinded interpretation of the CT or CBCT.

We considered the bias to be “**Low Risk**” if we scored “**Yes**” for both signalling questions; “***High Risk***” if we scored “***No***” for either of the questions; and “***Unclear***” if we scored “***Unclear***” for either of the signalling questions and the other signalling question was answered as “***Yes***”.

***Concerns regarding applicability: are there concerns that the target condition, as defined by the reference standard, does not match the question?***

The applicability to this study was related to which fractures were defined by the CT and CBCT as outcomes. We defined any midfacial fracture as an outcome, classified as frontal sinus, maxillary sinus, nasal, nasoorbitoethmoid, zygomaticomaxillary, orbital, maxillary or Le Fort type fracture. We judged the study as “***Low Applicability Concern***” if any midfacial fracture was defined as the primary outcome of the study; “***High Applicability Concern***” if a different outcome than any midfacial fracture was chosen (e.g., a specific midfacial fracture only or midfacial and mandibular fractures); “***Unclear Applicability Concern***” if insufficient information was provided to assess which fracture was defined as an outcome.

**Domain 4. Flow and timing**

***Risk of bias: Could the patient flow have introduced bias?***

- Signalling question 1: Was there an appropriate interval between the index and reference test?

A “***Yes***” was scored when the index and reference tests were performed within the same emergency department or outpatient clinic visit or whether the reference test was performed within 7 days of admission; “***No***” when the index and reference tests were not performed within the same emergency department or outpatient clinic visit or when the reference test was not performed within 7 days of admission.; “***Unclear***” if no information was provided on the interval between the index and reference tests.

- Signalling question 2: Did all the patients receive a reference standard?

A “***Yes***” was scored if all the patients had received a reference standard: “***No***” if not all the patients had received a reference standard; “***Unclear***” if insufficient information was provided to assess if all the patients had received a reference standard.

- Signalling question 3: Did all the patients receive the same reference standard?

A “***Yes***” was scored if all the patients had received the same reference standard: “***No***” if not all the patients had received the same reference standard; “***Unclear***” if insufficient information was provided to assess if all the patients had received the same reference standard.

- Signalling question 4: Were all the patients included in the analysis?

A “***Yes***” was scored if all the patients were included in the analysis: “***No***” if not all the patients were included in the analysis; “***Unclear***” if insufficient information was provided to assess whether all the patients were included in the analysis.

We considered the bias to be “***Low Risk***” if we scored “***Yes***” for all the signalling questions; “***High Risk***” if we scored “***No***” for any of the questions; and “***Unclear***” if we scored “***Unclear***” for any of the signalling questions and the other signalling questions were answered as “***Yes***”.

**Supplementary material S3**: Excluded articles with reasons after full-text screening.

| *#* | *Author* | *Year* | *Reason for exclusion* |
| --- | --- | --- | --- |
| 1 | Allison et al. [1] | 2017 | Conference abstract. |
| 2 | Aslan et al. [2] | 2019 | Not enough data to construct 2x2 contingency tables and no data given regarding sensitivity, specificity, negative predictive value, positive predictive value, positive likelihood ratio or negative likelihood ratio. |
| 3 | Coloma et al. [3] | 2013 | Conference abstract. |
| 4 | Bofano et al. [4] | 2014 | Not enough data to construct 2x2 contingency tables and no data given regarding sensitivity, specificity, negative predictive value, positive predictive value, positive likelihood ratio or negative likelihood ratio. |
| 5 | Chow et al. [5] | 2018 | Not enough data to construct 2x2 contingency tables and no data given regarding sensitivity, specificity, negative predictive value, positive predictive value, positive likelihood ratio or negative likelihood ratio. |
| 6 | Daniel et al. [6] | 2005 | Study did not assess the diagnostic accuracy of the physical examination findings compared to the reference standard for midfacial trauma patients.  Study does not report a reference test. |
| 7 | Etufugh et al. [7] | 2000 | Study did not assess the diagnostic accuracy of the physical examination findings compared to the reference standard for midfacial trauma patients.  Not enough data to construct 2x2 contingency tables and no data given regarding sensitivity, specificity, negative predictive value, positive predictive value, positive likelihood ratio or negative likelihood ratio. |
| 8 | Finnerty et al. [8] | 2016 | Conference abstract. |
| 9 | Gerlock et al. [9] | 1977 | Narrative review.  Study did not assess the diagnostic accuracy of the physical examination findings compared to the reference standard for midfacial trauma patients. |
| 10 | Gunasekaran et al. [10] | 2014 | Conference abstract. |
| 11 | Gunasekaran et al. [11] | 2014 | Conference abstract. |
| 12 | Haworth et al. [12] | 2017 | Plain facial radiography was used as a reference test, instead of Computed Tomography or Cone Beam Computed Tomography. |
| 13 | Holmgren et al. [13] | 2004 | Not enough data to construct 2x2 contingency tables and no data given regarding sensitivity, specificity, negative predictive value, positive predictive value, positive likelihood ratio or negative likelihood ratio. |
| 14 | Pérez-Guisado et al. [14] | 2012 | The reference standard was a combination of all te clinical data combined with x-ray findings (nasal bones and waters projections), intraoperative findings, and CT scans. The authors did not define which patients receive CT as reference standard. |
| 15 | Siritongtaworn et al. [15] | 2020 | The study did not include midfacial trauma patients with suspected midfacial fractures. Only patients with a confirmed fracture were included.  The reference standard was unclear.  Not enough data to construct 2x2 contingency tables and no data given regarding sensitivity, specificity, negative predictive value, positive predictive value, positive likelihood ratio or negative likelihood ratio. |
| 16 | Thai et al. [16] | 1997 | Plain roentgenograms or facial CT scans were used as a reference standard.  Not enough data to construct 2x2 contingency tables and no data given regarding sensitivity, specificity, negative predictive value, positive predictive value, positive likelihood ratio or negative likelihood ratio. |
| 17 | Timashpolsky et al. [17] | 2015 | Conference abstract. |
| 18 | Welman et al. [18] | 2016 | Study did not compare the diagnostic properties of the clinical examination to Computed Tomography or Cone Beam Computed Tomography datasets from patients with midfacial trauma. The study focused on whether a thorough initial visual assessment is associated with improved ophthalmic outcomes.  Not enough data to construct 2x2 contingency tables and no data given regarding sensitivity, specificity, negative predictive value, positive predictive value, positive likelihood ratio or negative likelihood ratio. |
| 19 | Whitesell et al. [19] | 2015 | Not enough data to construct 2x2 contingency tables and no data given regarding sensitivity, specificity, negative predictive value, positive predictive value, positive likelihood ratio or negative likelihood ratio. |
| 20 | Yadav et al. [20] | 2014 | Conference abstract. |

**Supplementary material S4**: Deeks funnel plots for the individual physical examination findings. Publication bias was presented plotting the diagnostic odds ratio against the inverse of the square root (sqrt) of effective sample size (ESS).

| Asymmetry due to midfacial and mandibular fractures 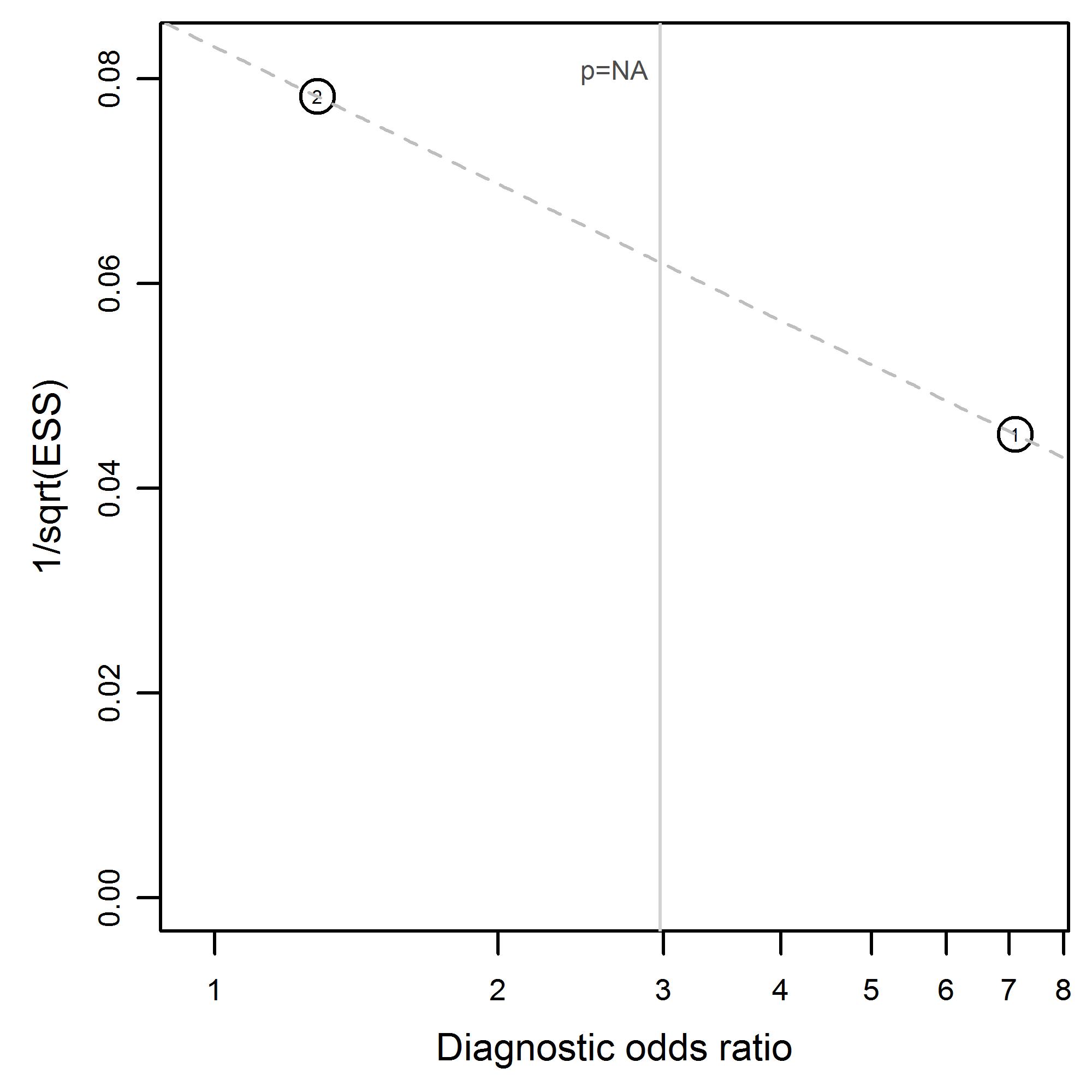 | Diplopia due to midfacial and mandibular fractures 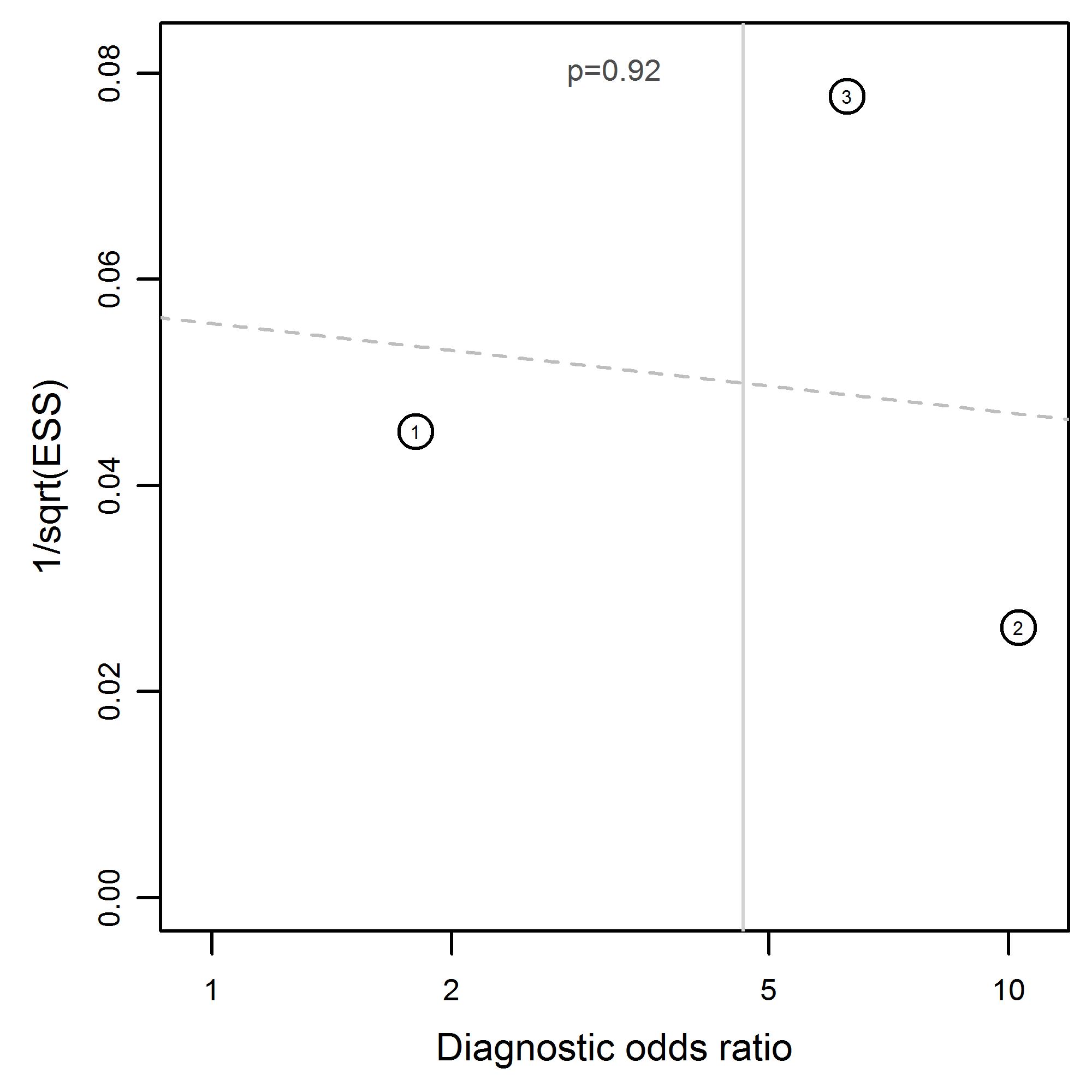 |
| --- | --- |
| Diplopia due to orbital fractures 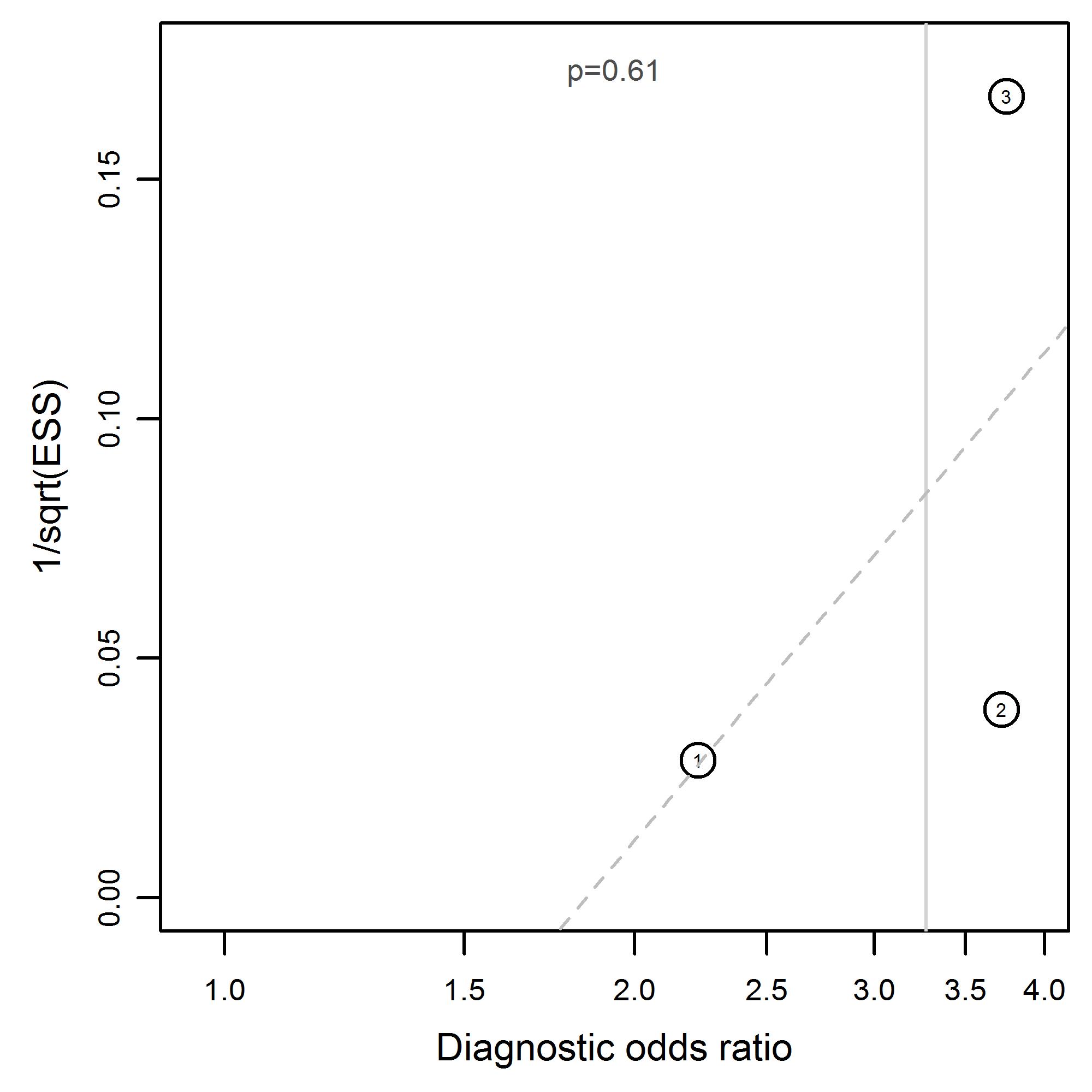 | Epistaxis due to midfacial and mandibular fractures 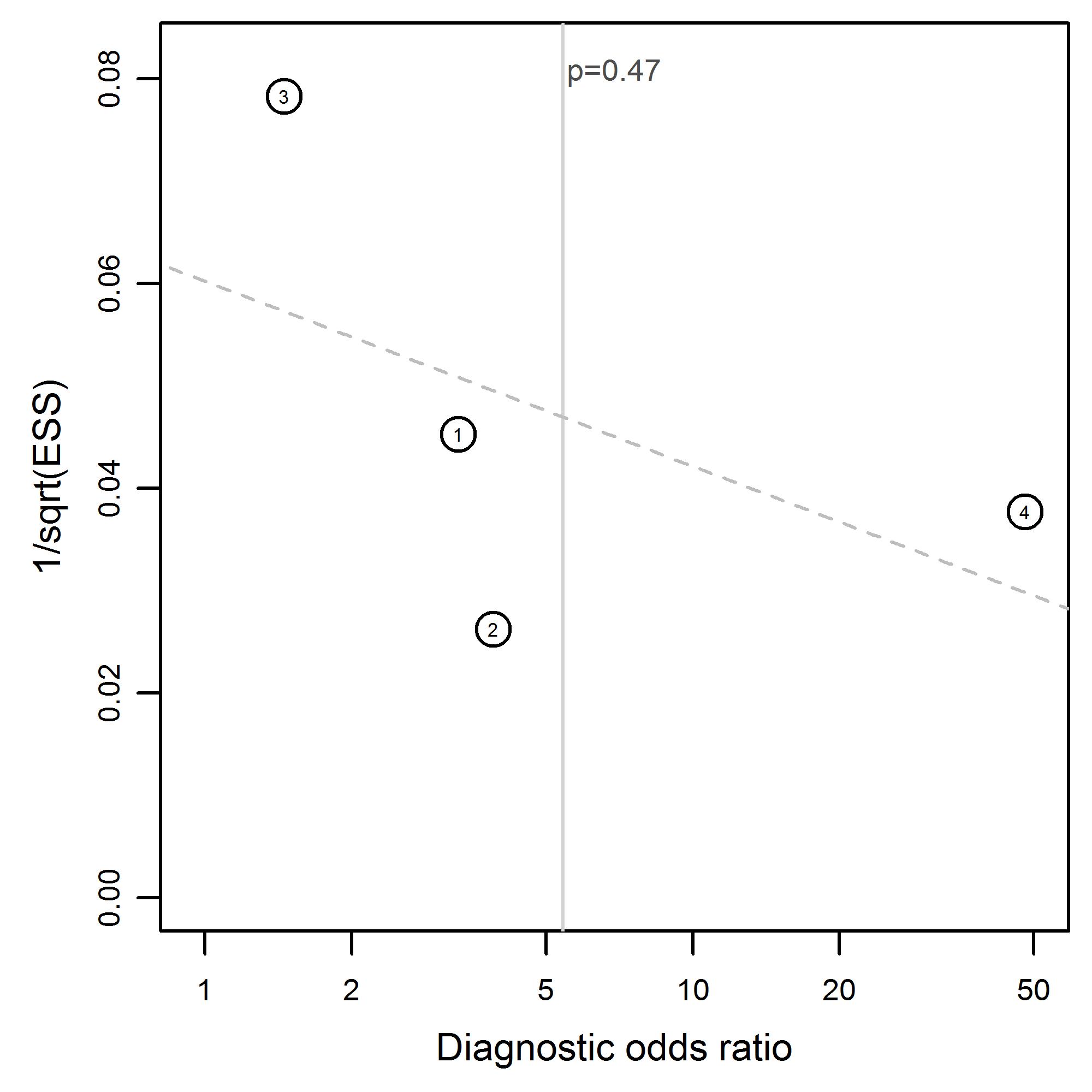 |
| Extra-ocular movement limitation due to midfacial and mandibular fractures 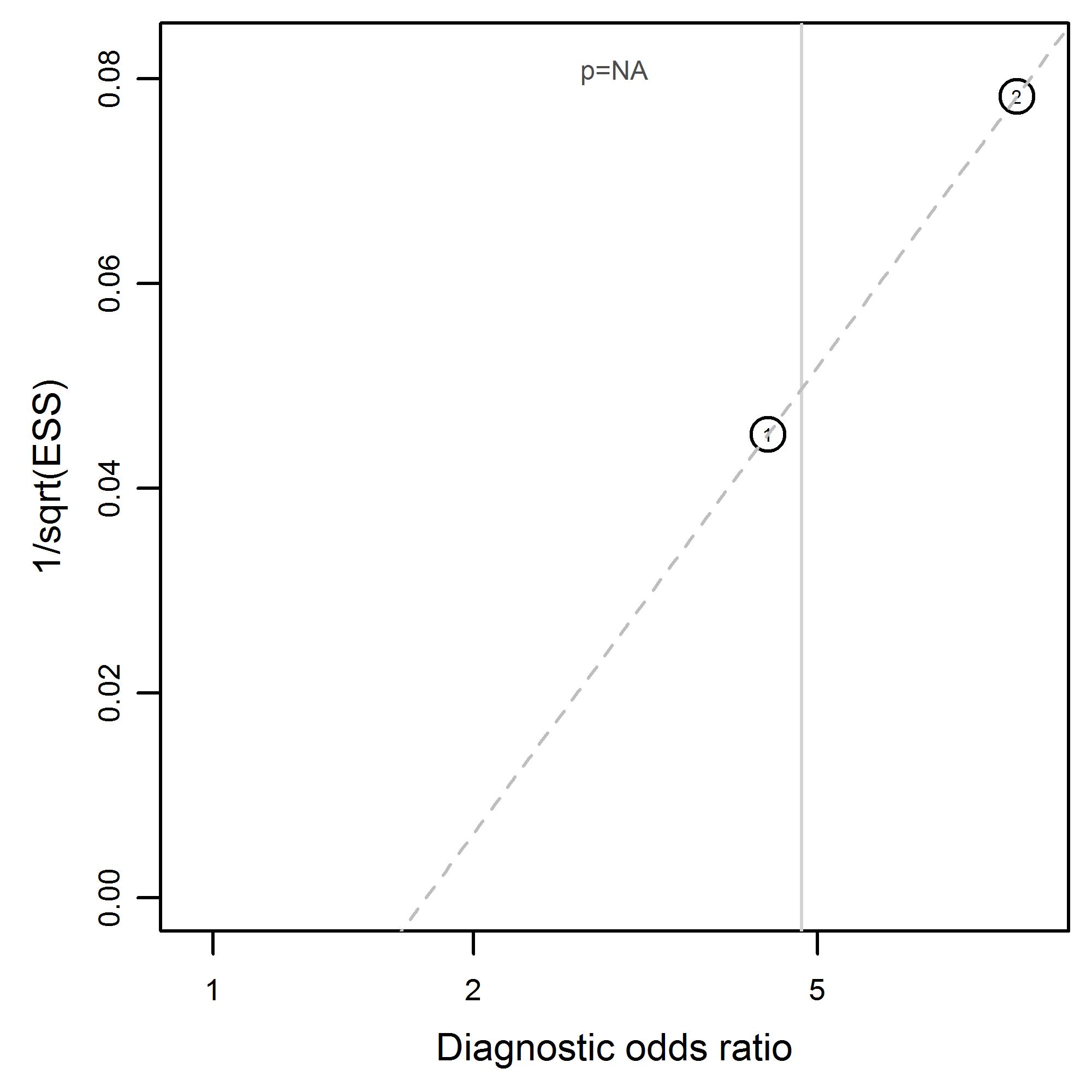 | Extra-ocular movement limitation due to orbital fractures 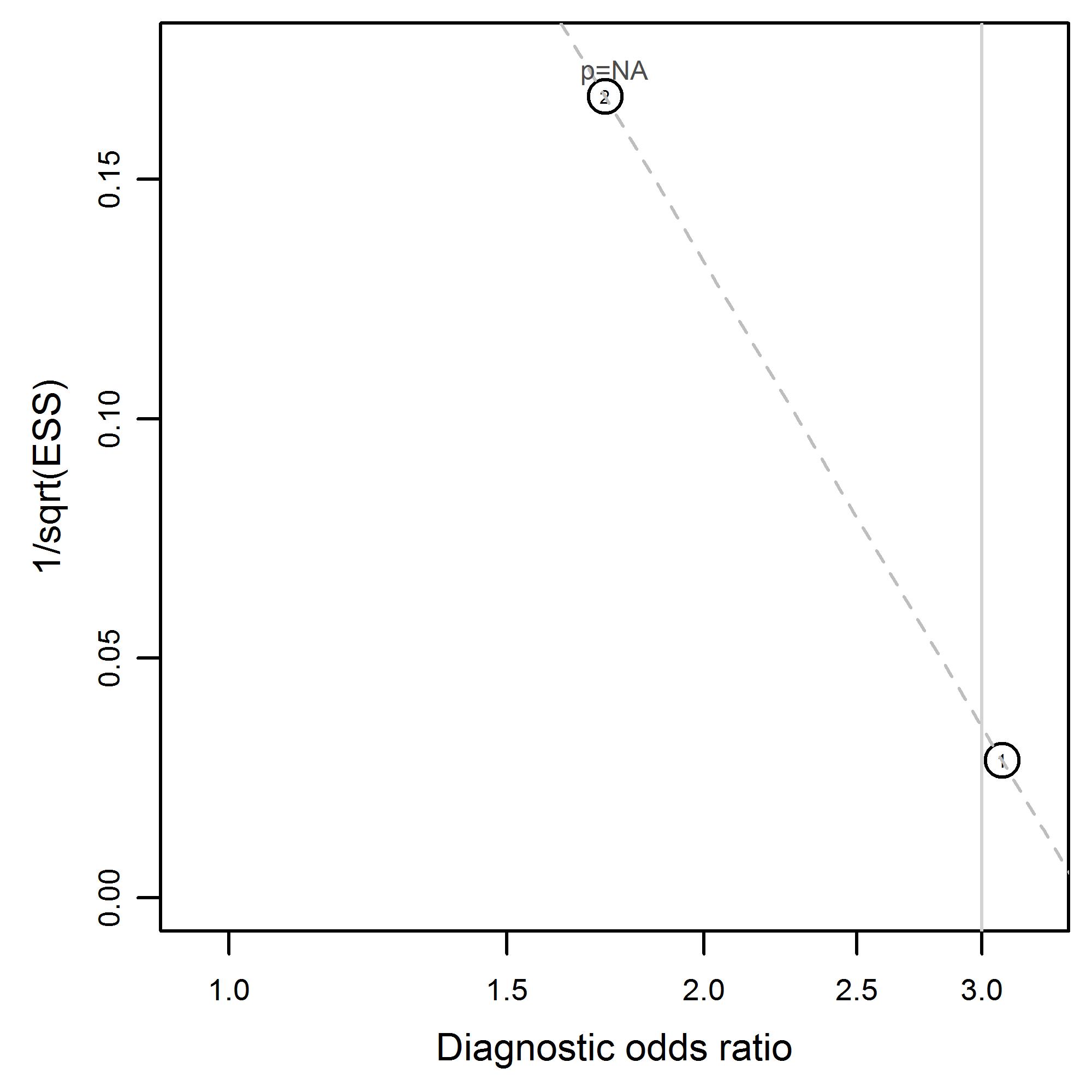 |
| Facial pain due to midfacial and mandibular fractures 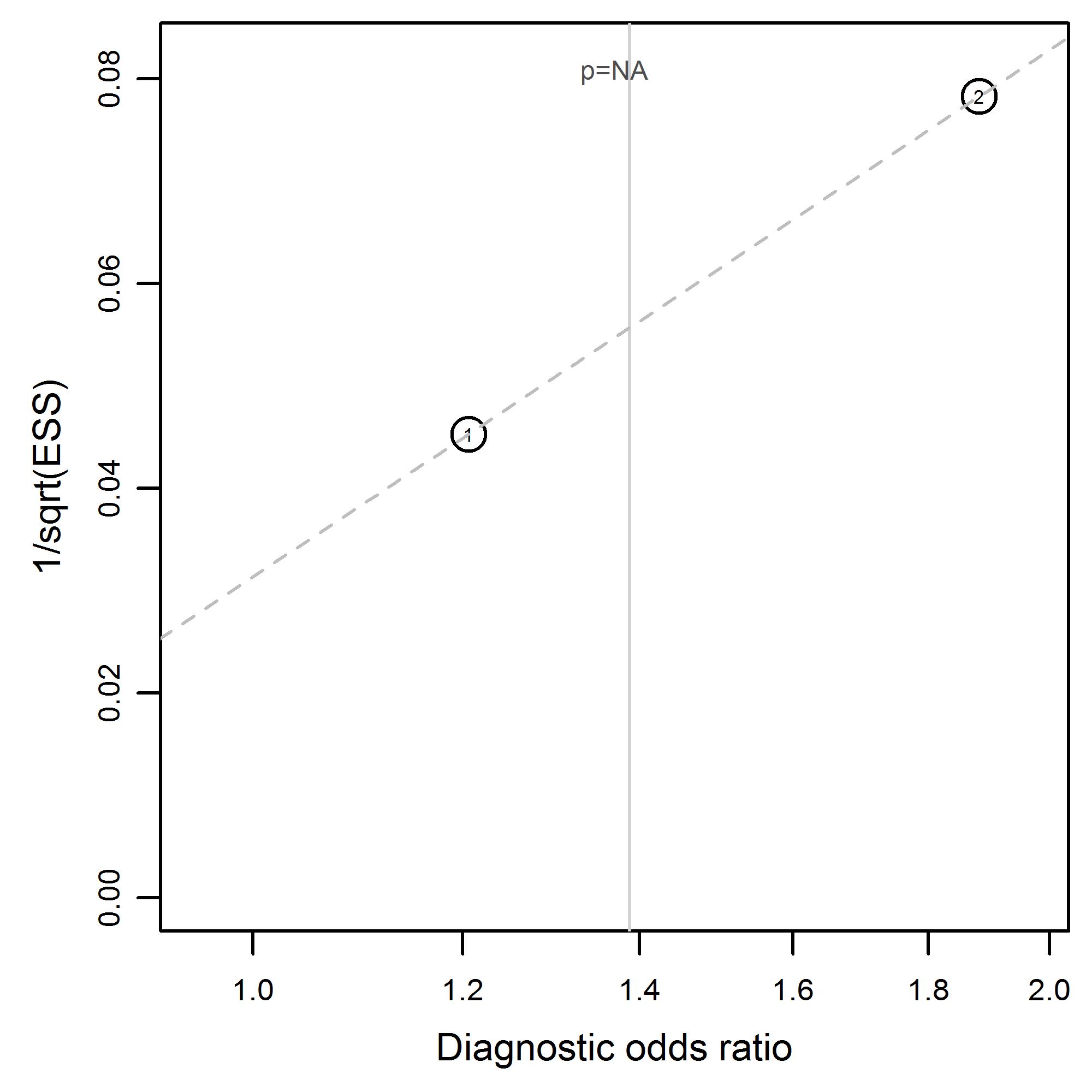 | Hematoma due to midfacial and mandibular fractures 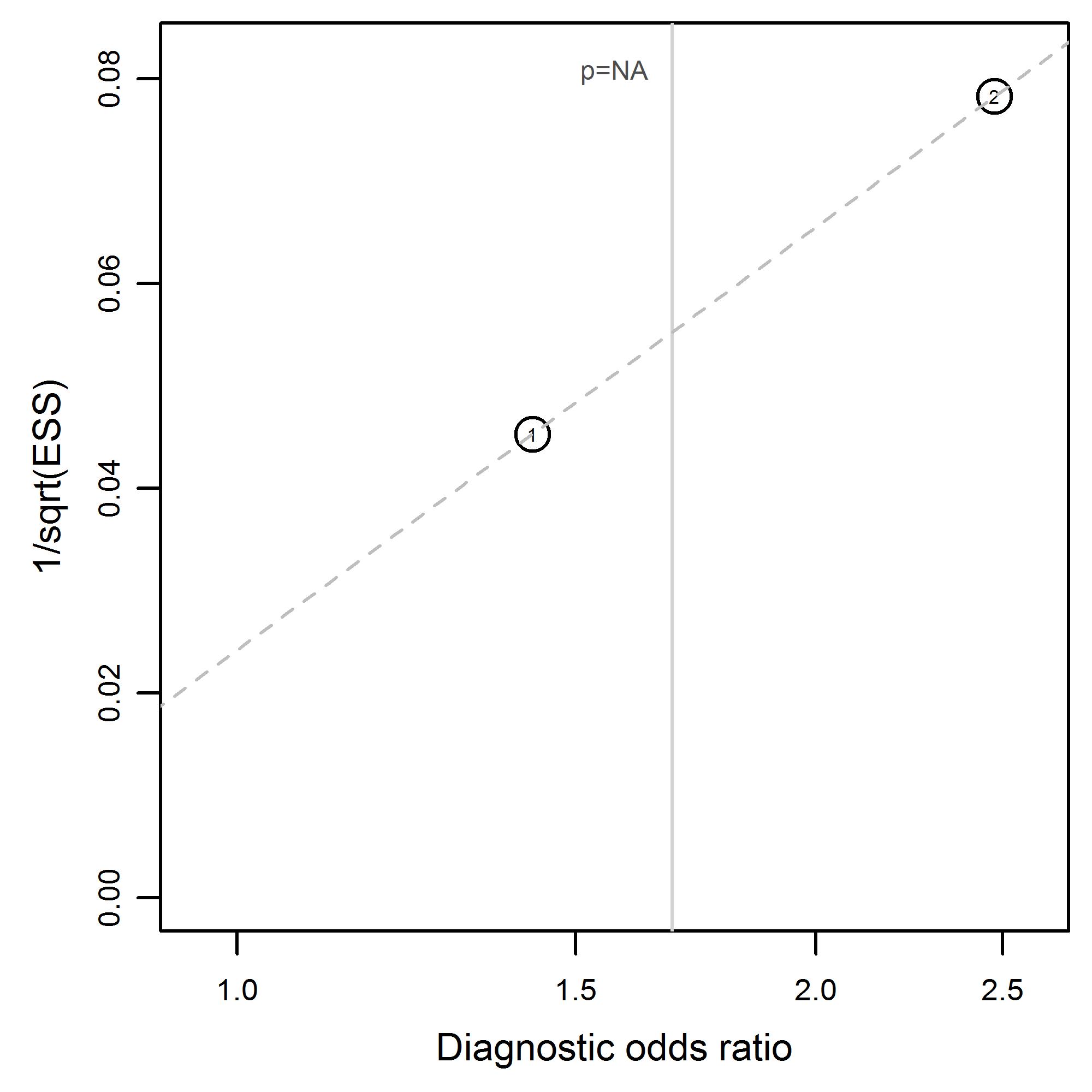 |
| Hematoma (forehead) due to midfacial and mandibular fractures 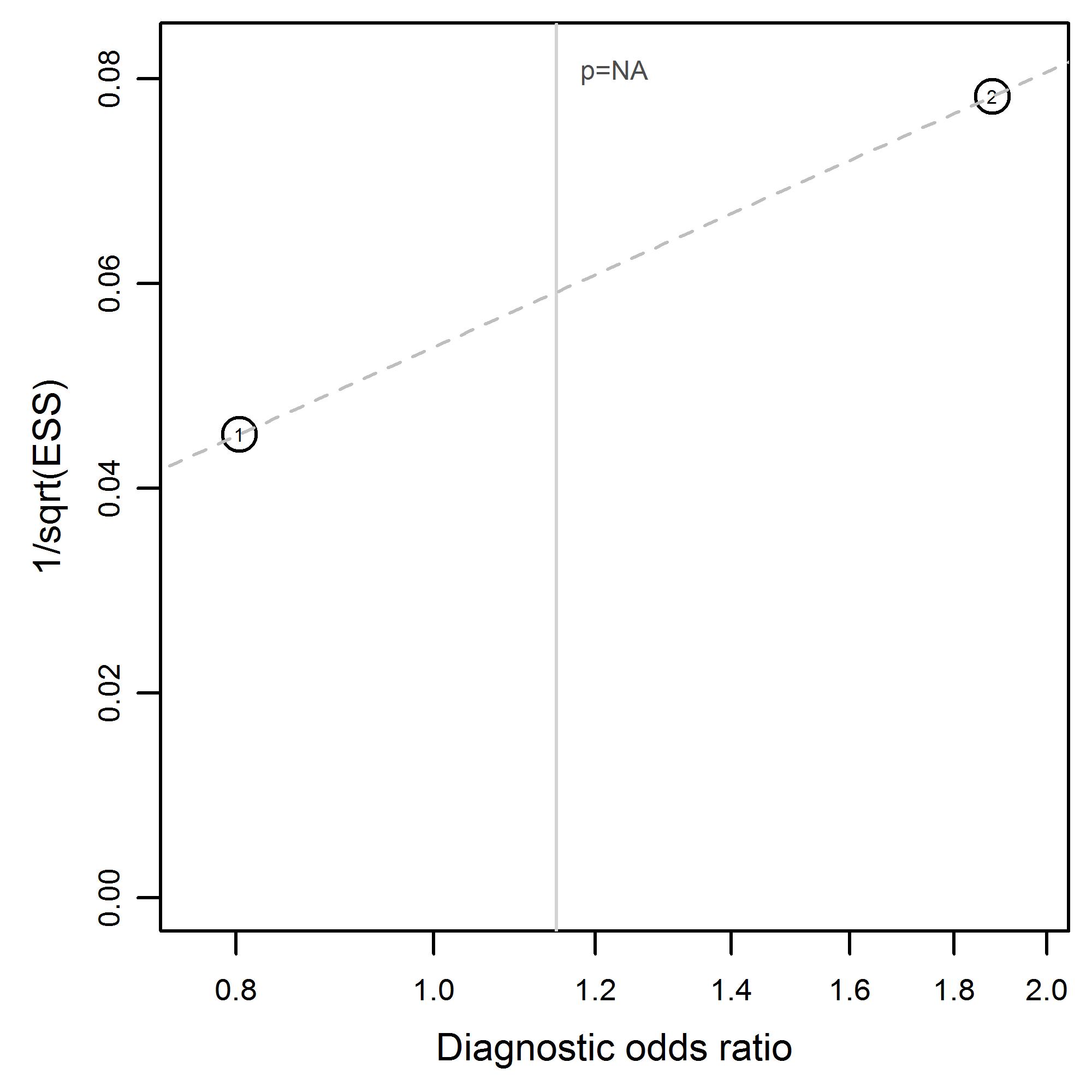 | Hematoma (malar region) due to midfacial and mandibular fractures 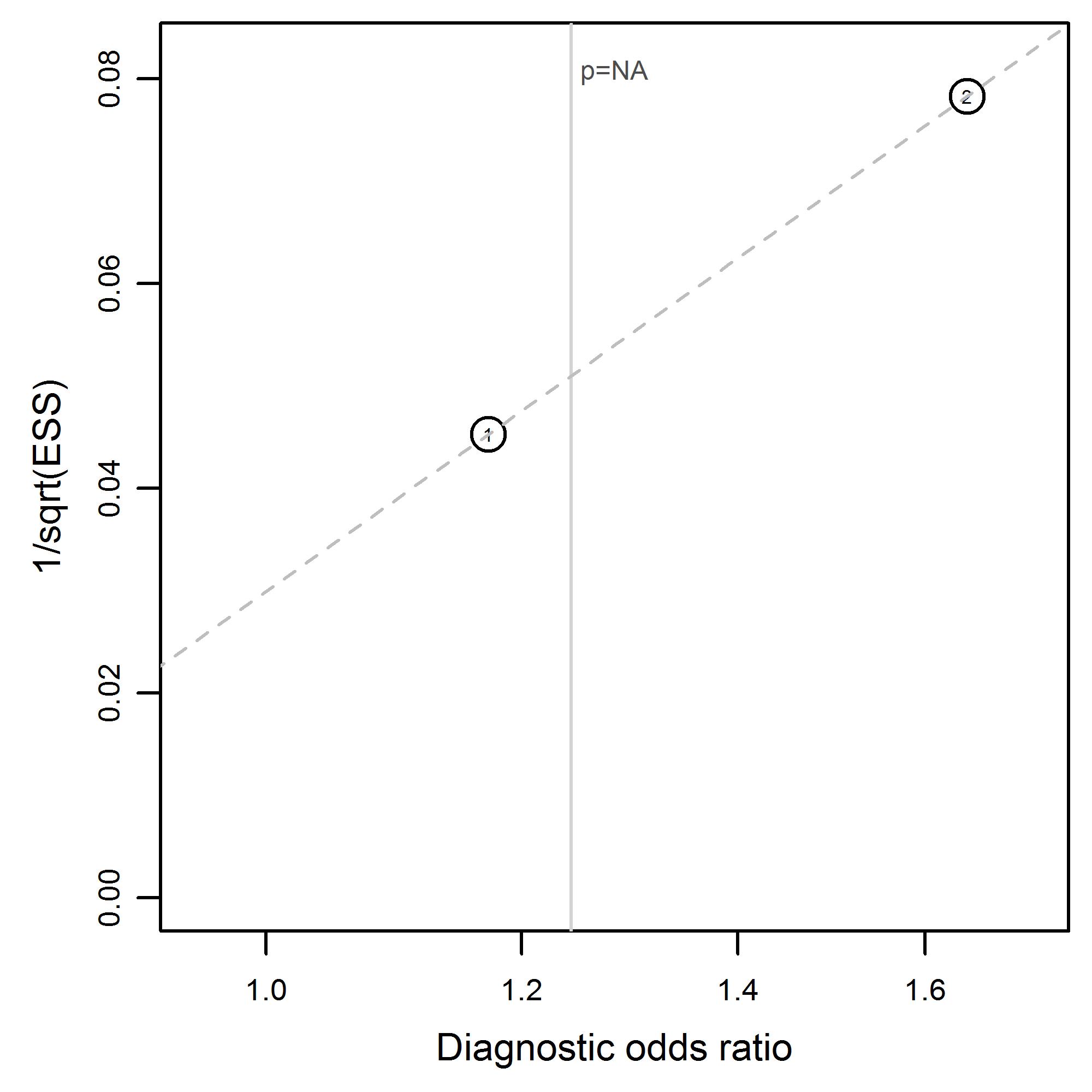 |
| Hematoma (nasal region) in the midfacial and mandibular region 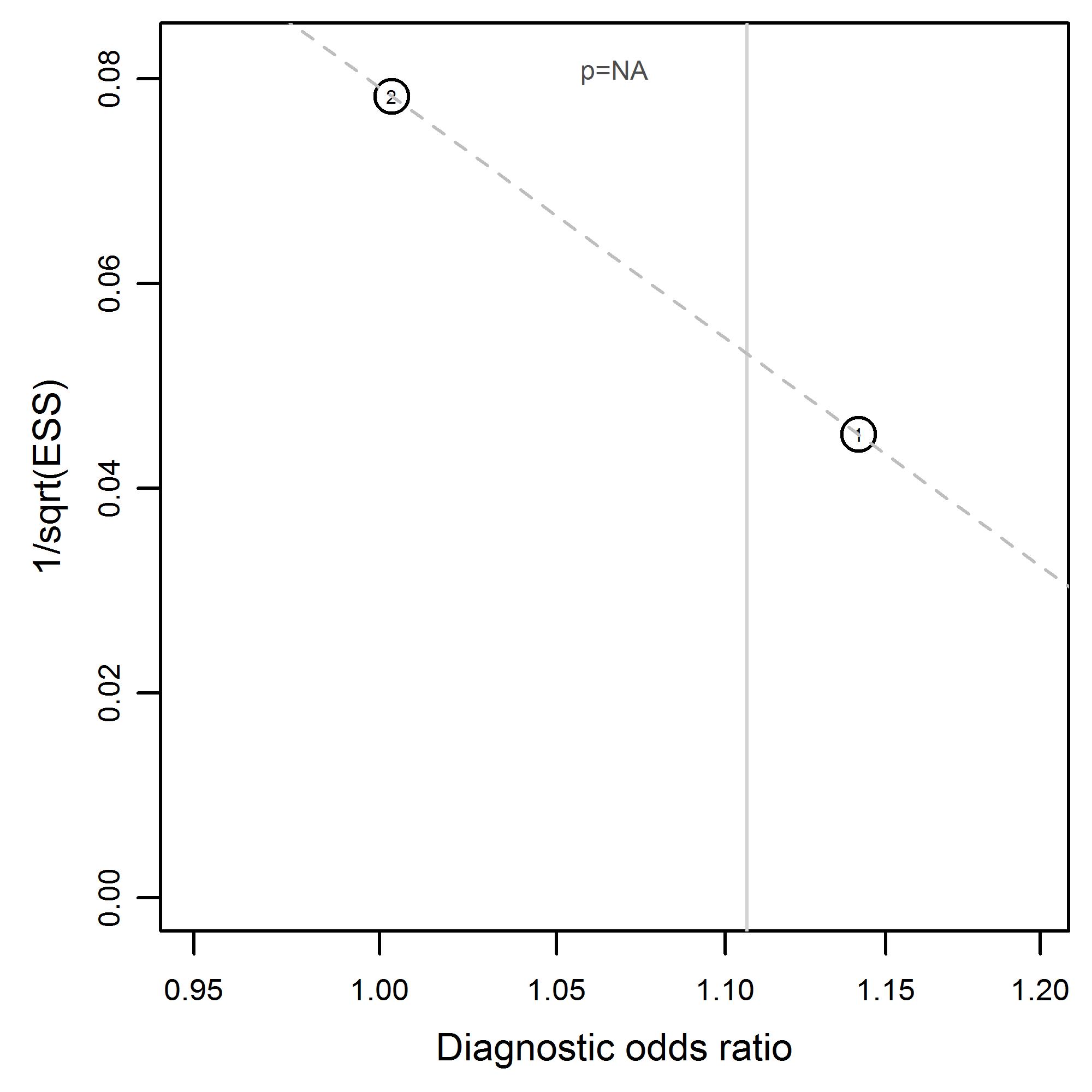 | Hematoma (peri-orbital) in to midfacial and mandibular region 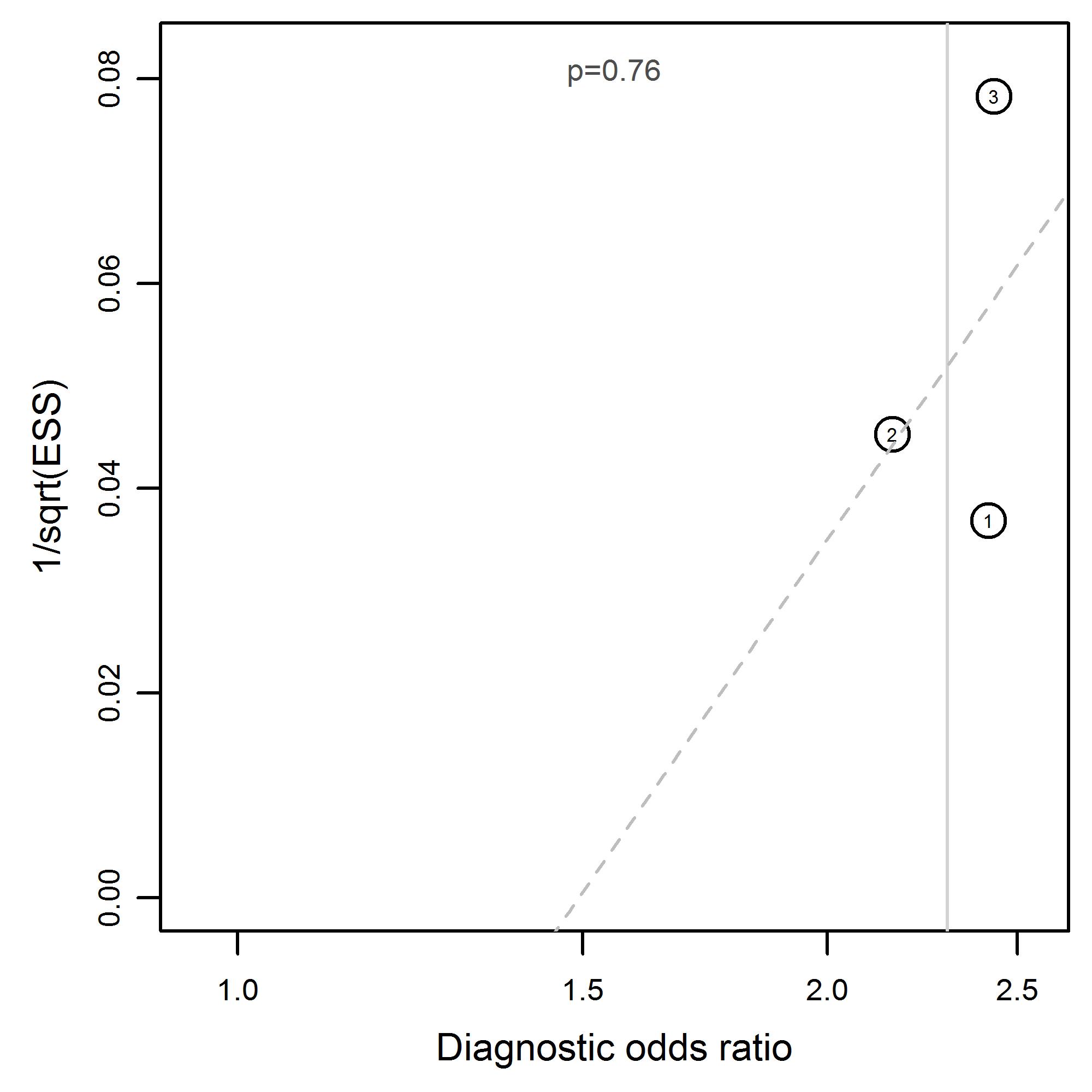 |
| Hematoma (peri-orbital) due to orbital fractures 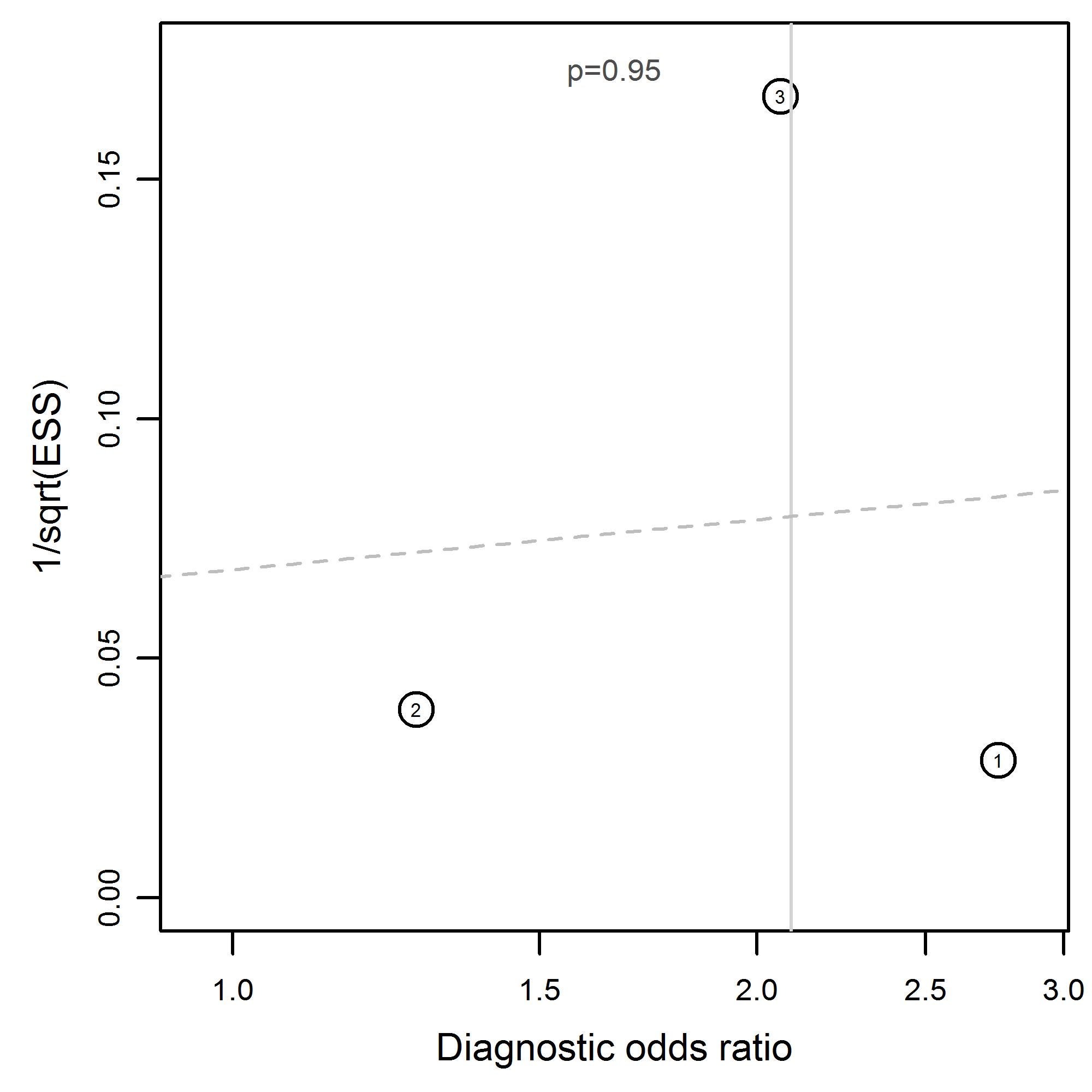 | Infra-orbital nerve paresthesia due to midfacial and mandibular fractures 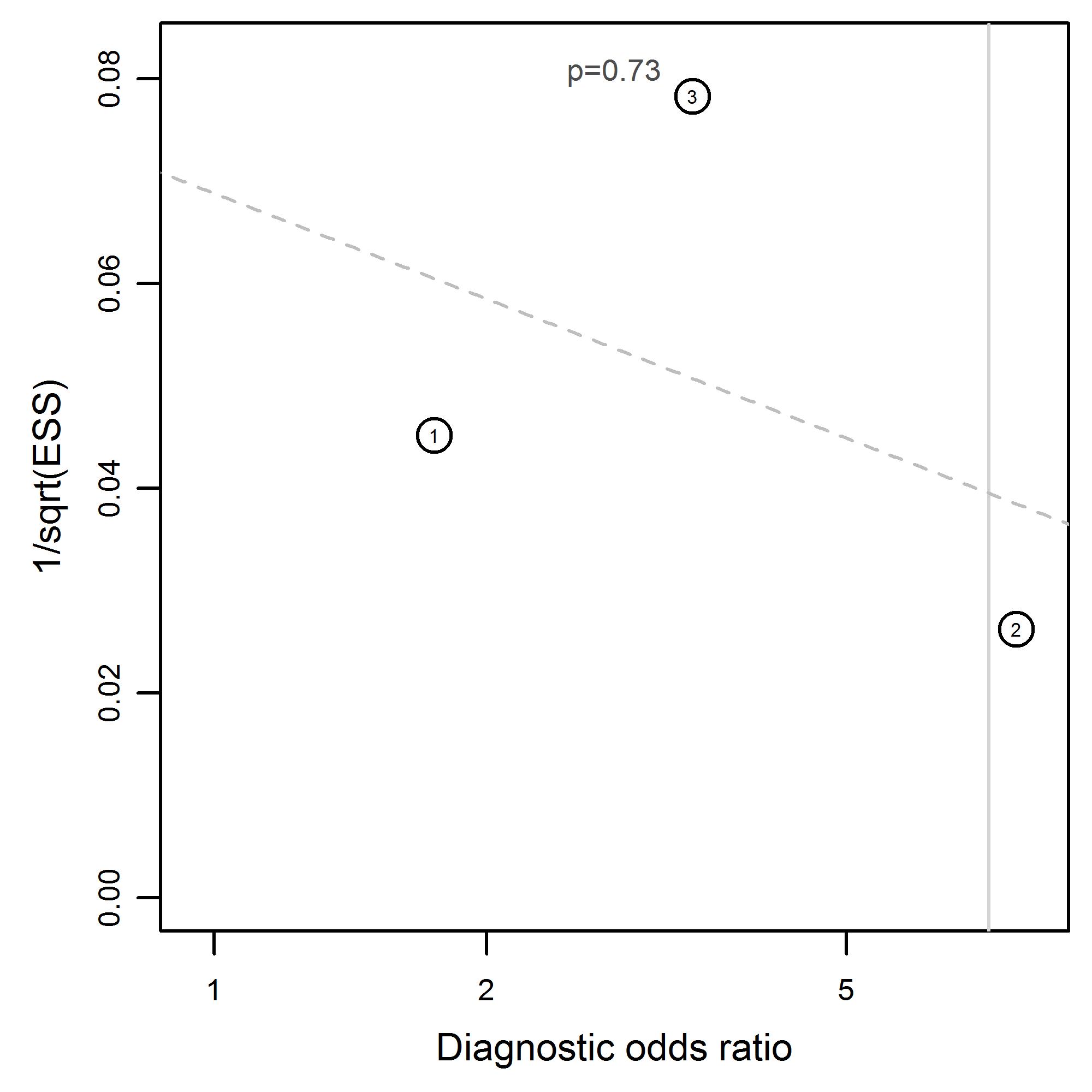 |
| Infra-orbital nerve paresthesia due to orbital fractures 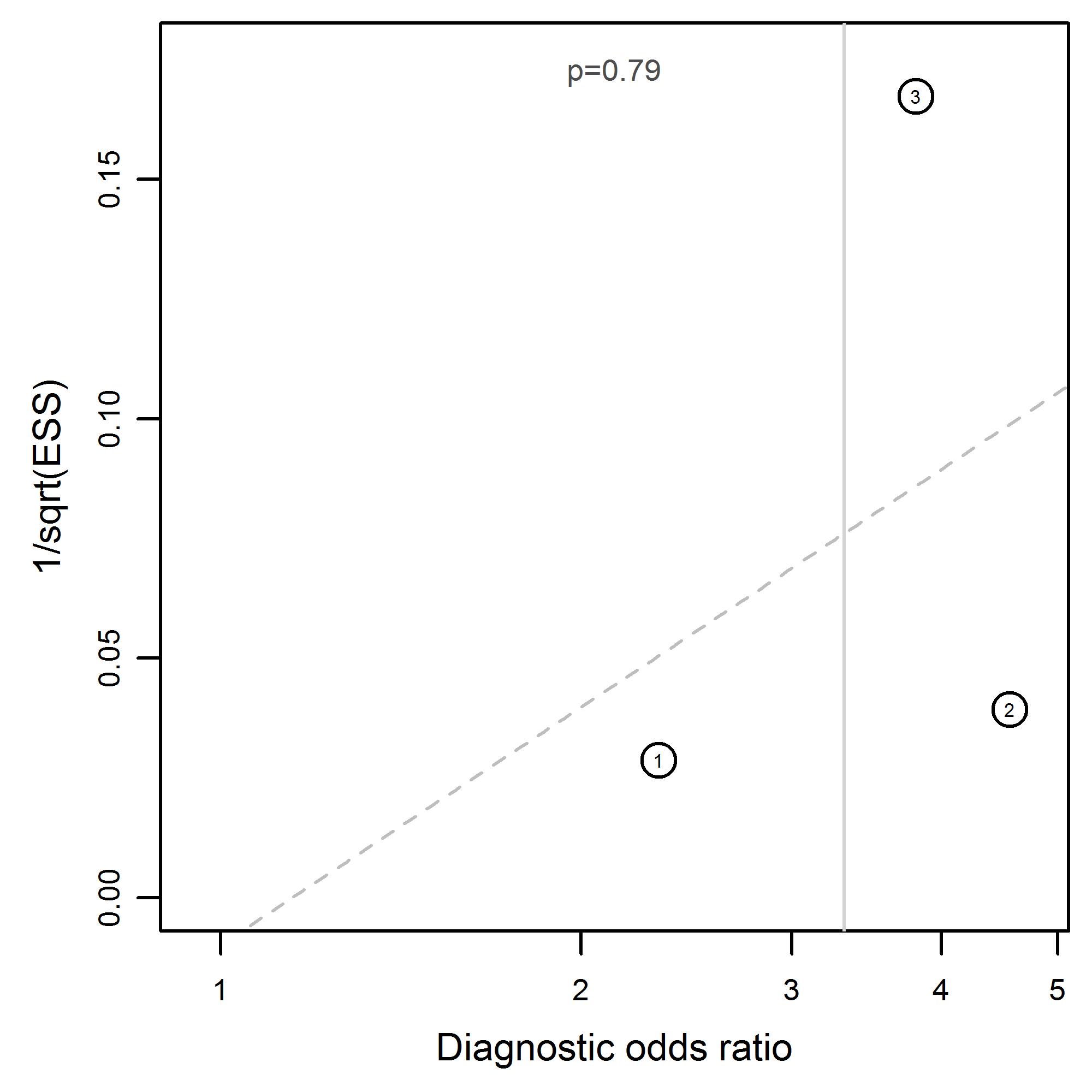 | Laceration with midfacial and mandibular fractures 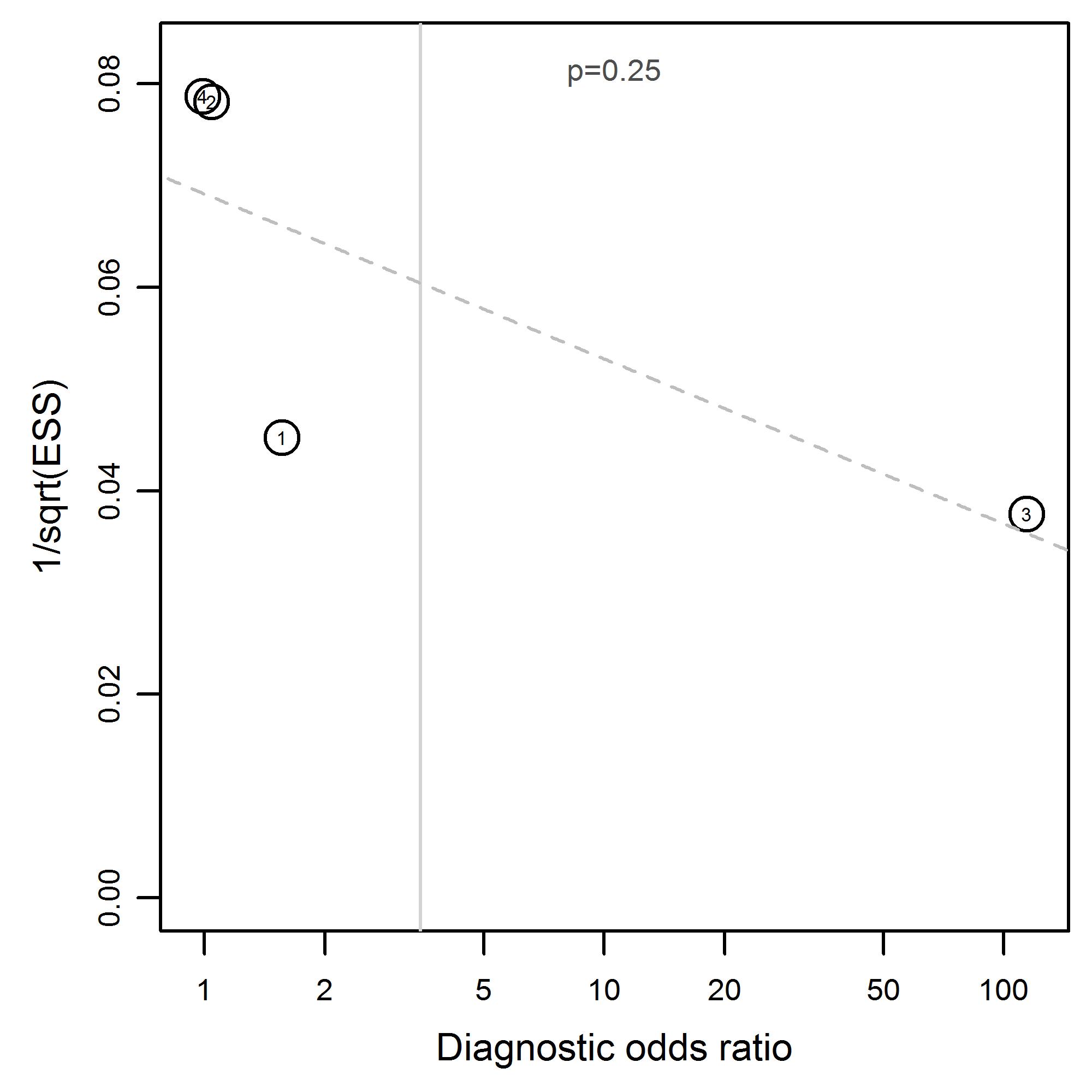 |
| Laceration (forehead) with midfacial and mandibular fractures 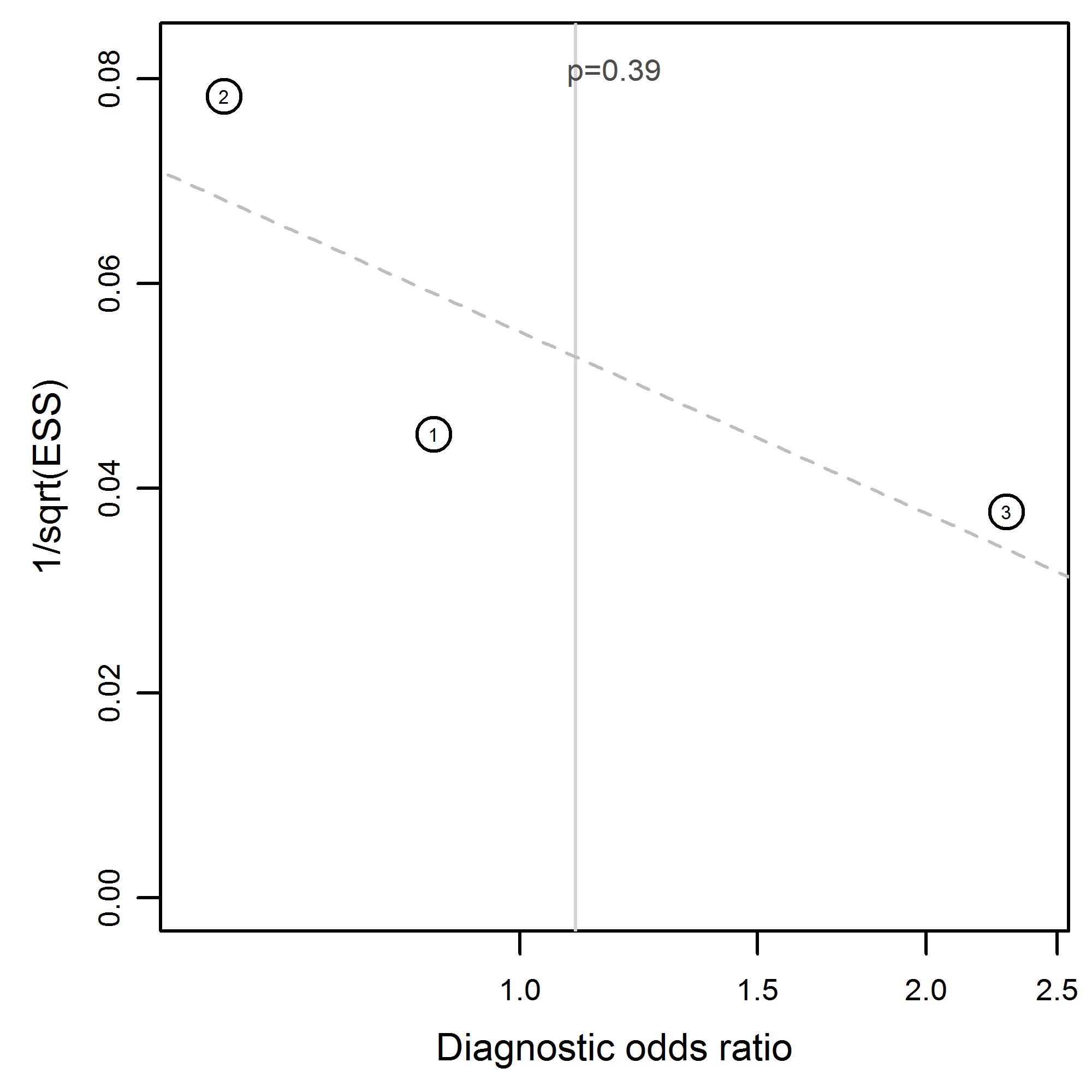 | Laceration (intra-oral) with midfacial and mandibular fractures 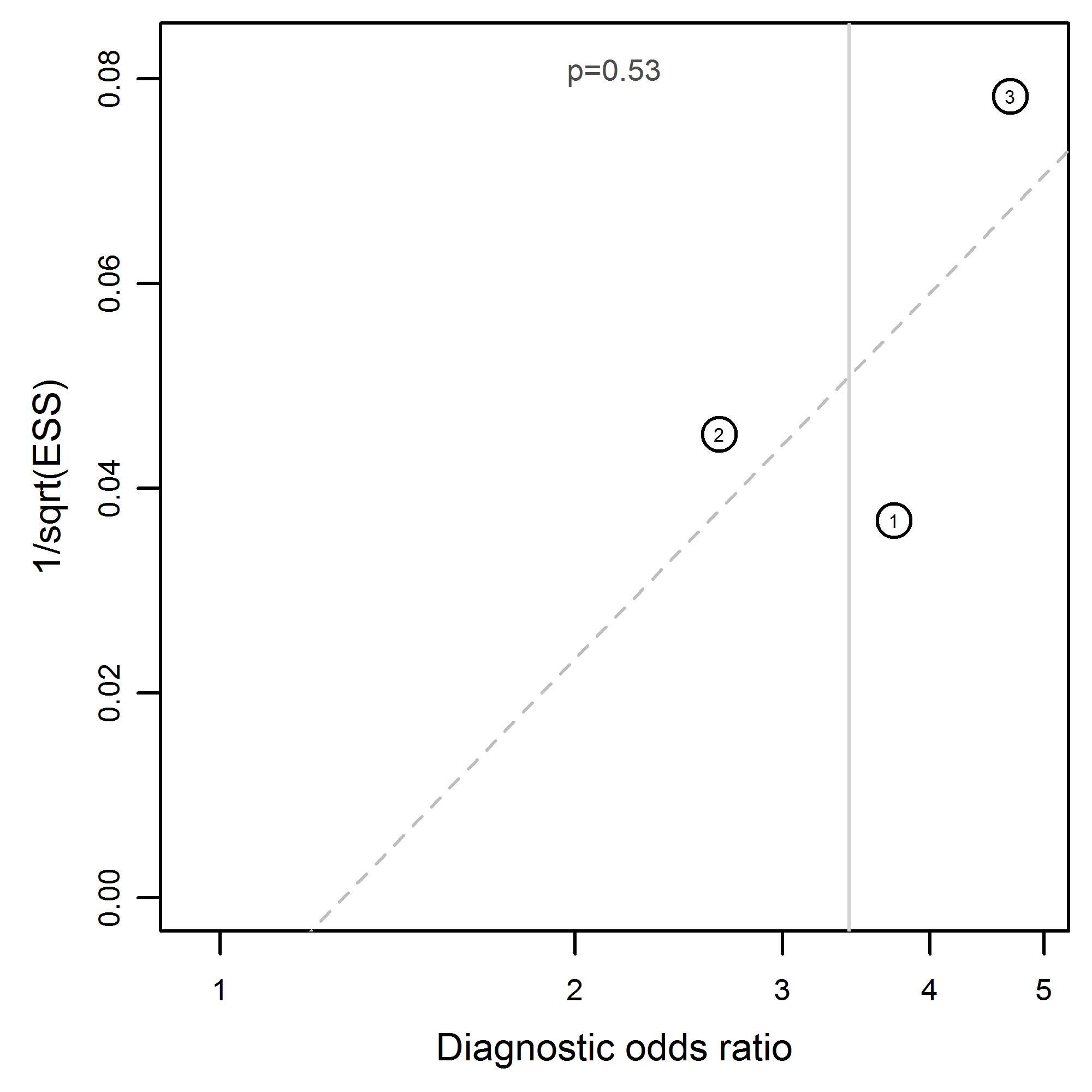 |
| Laceration (malar) with midfacial and mandibular fractures 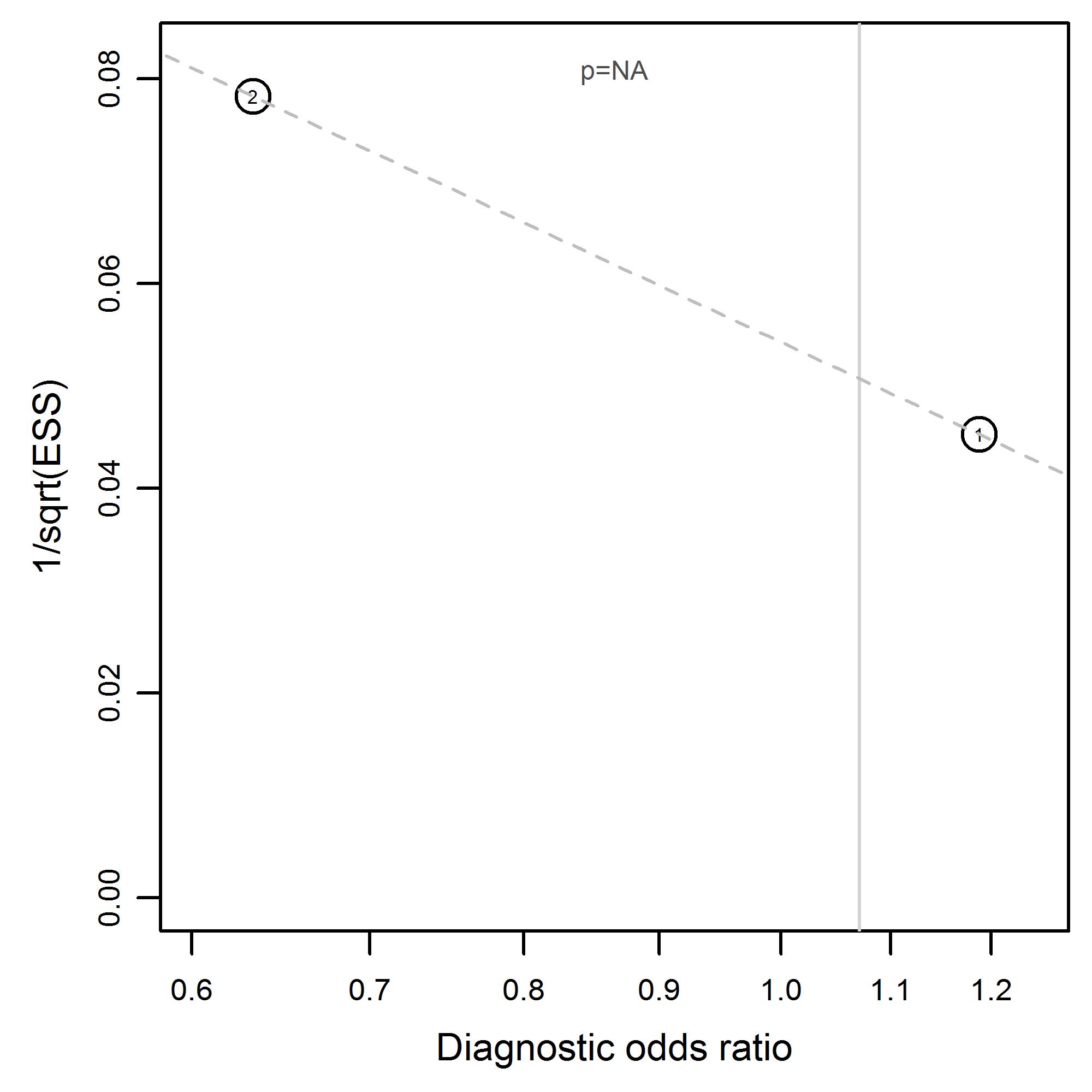 | Laceration (nasal) with midfacial and mandibular fractures 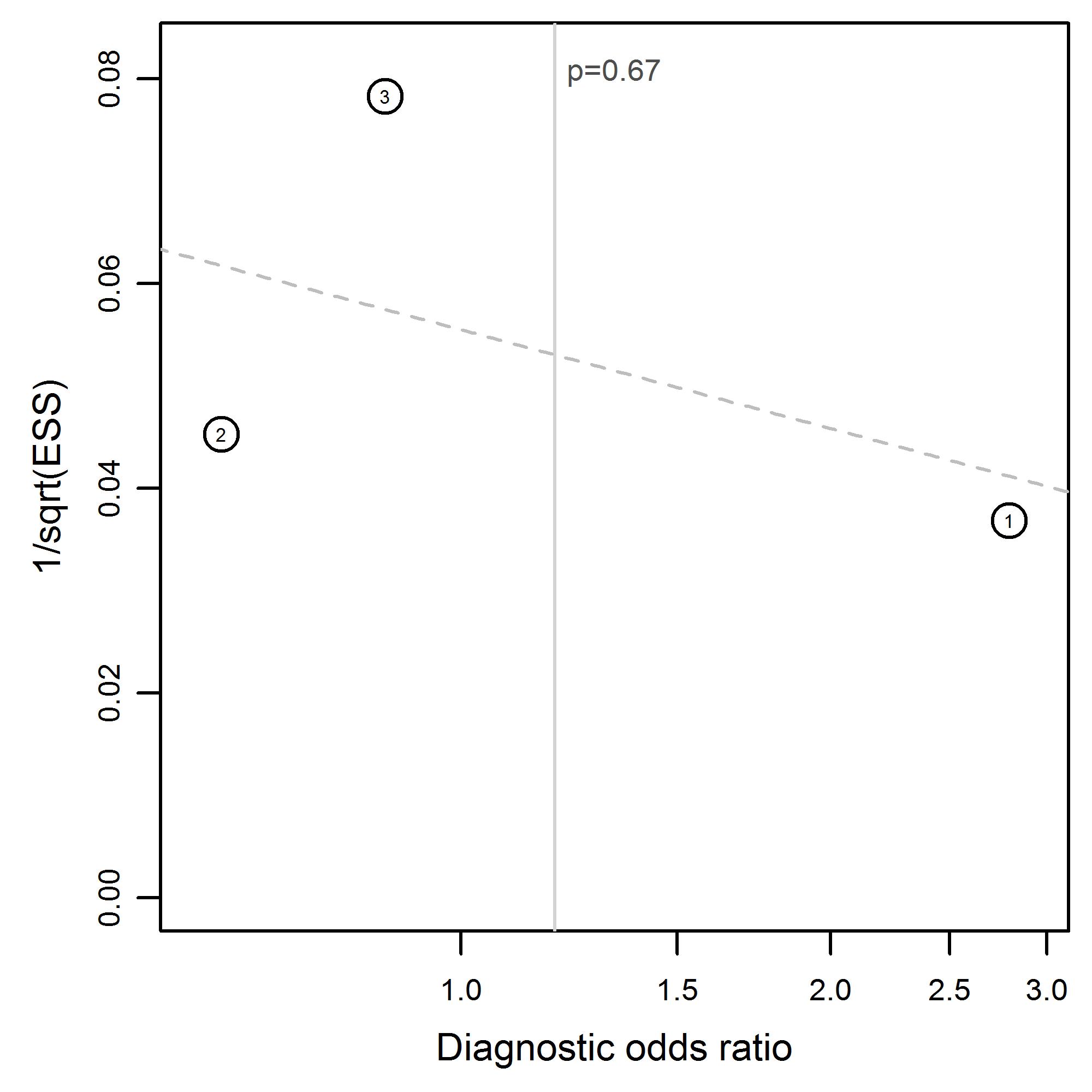 |
| Laceration (peri-oral) due to midfacial and mandibular fractures 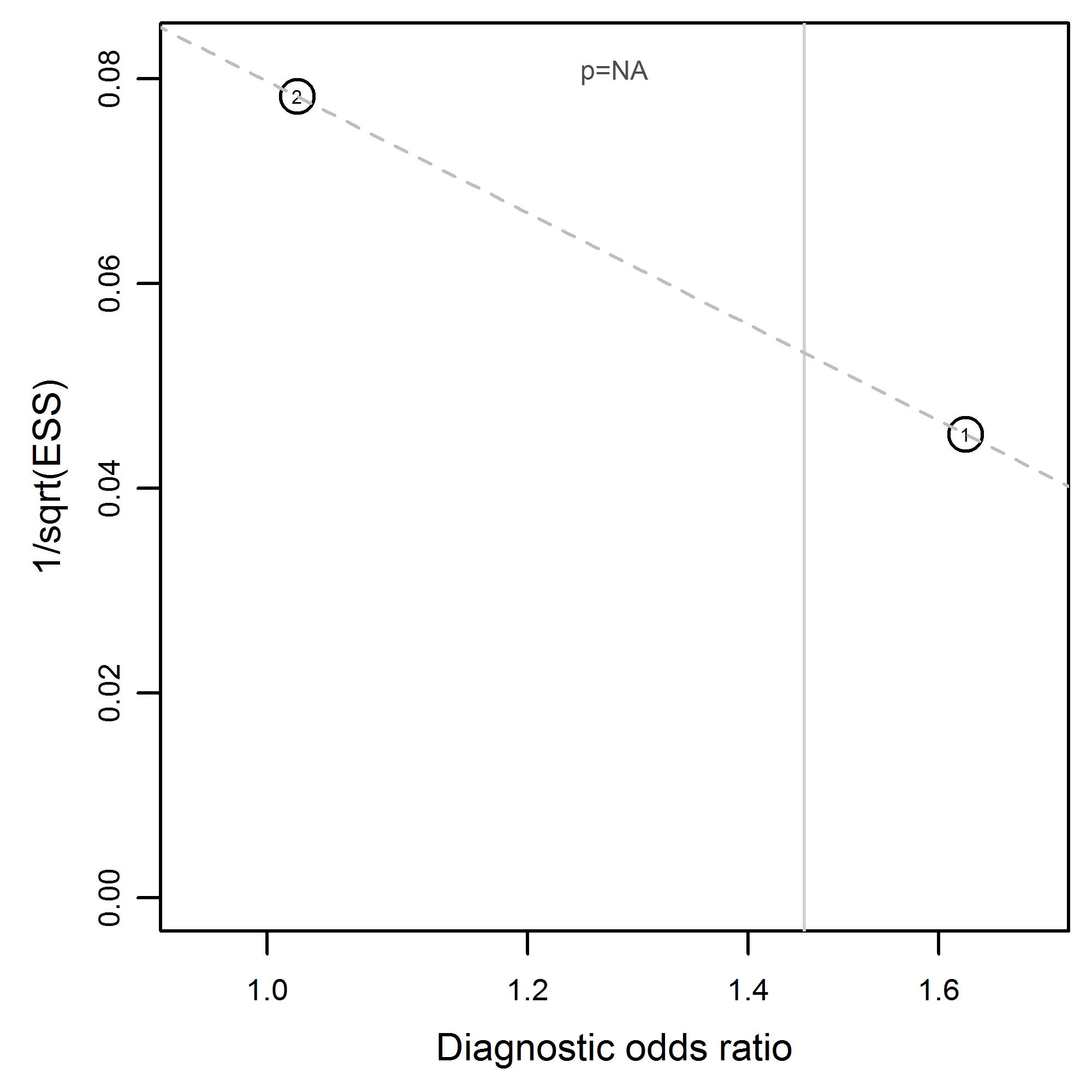 | Laceration (peri-orbital) due to midfacial and mandibular fractures 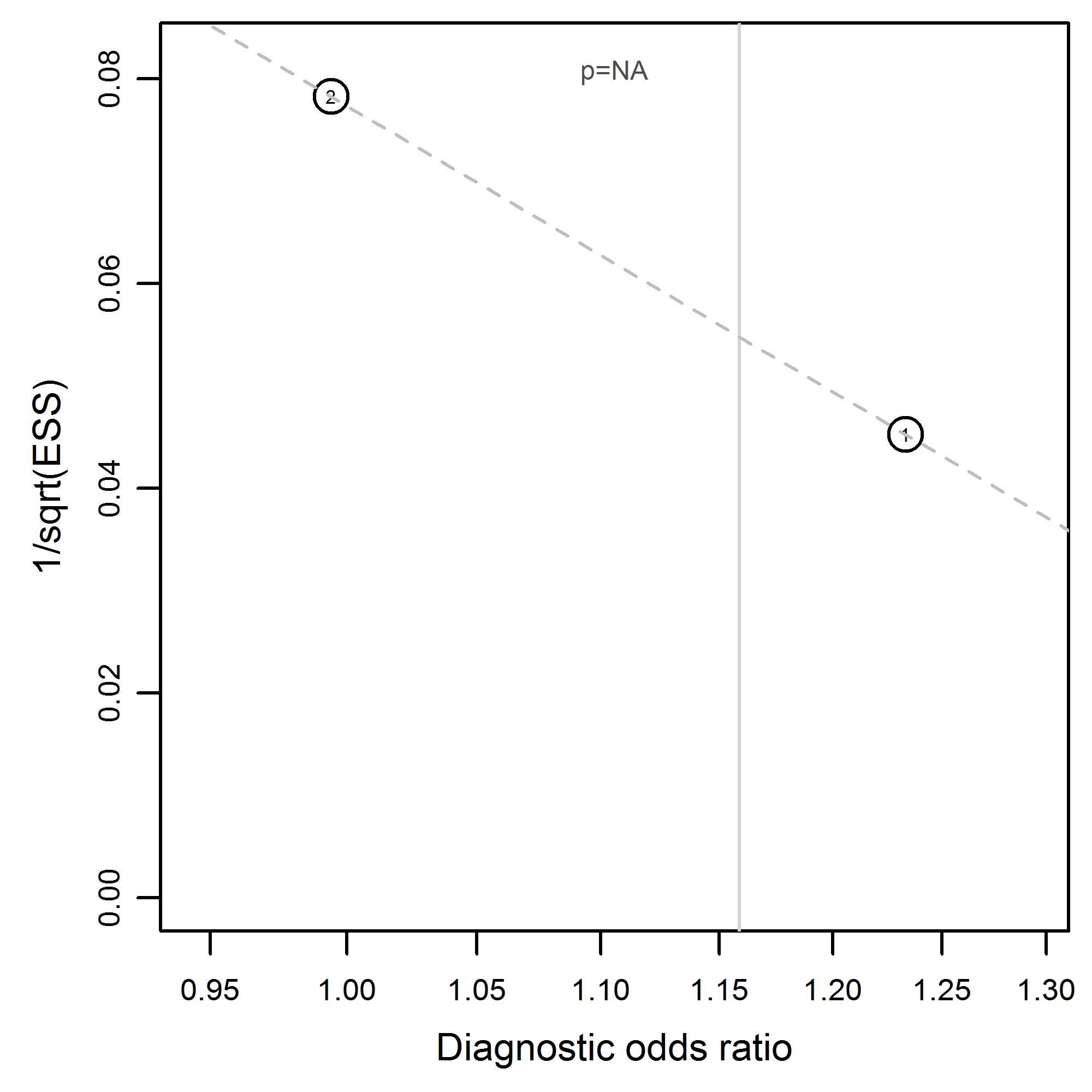 |
| Malocclusion due to midfacial and mandibular fractures 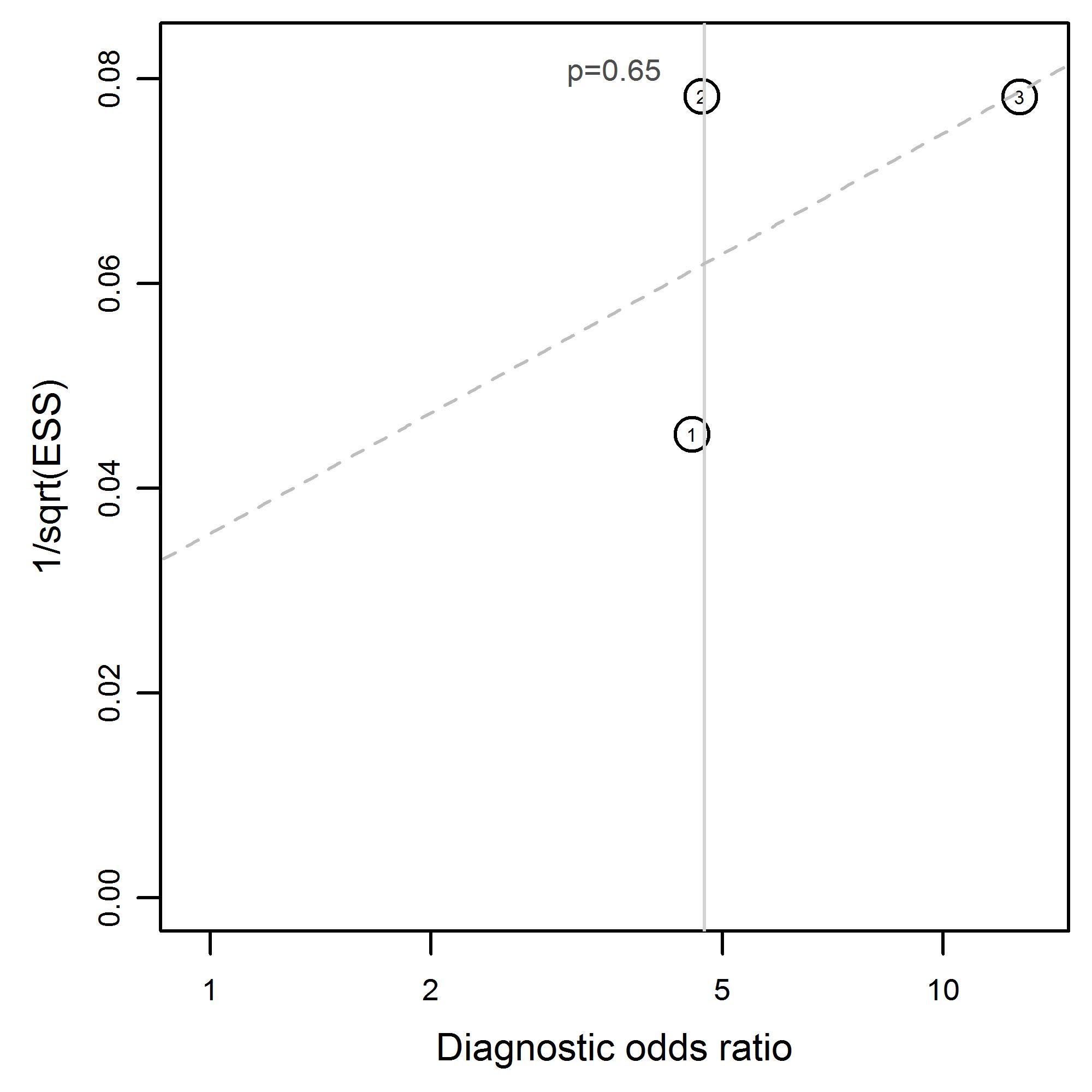 | Open fractures in midfacial and mandibular fractures 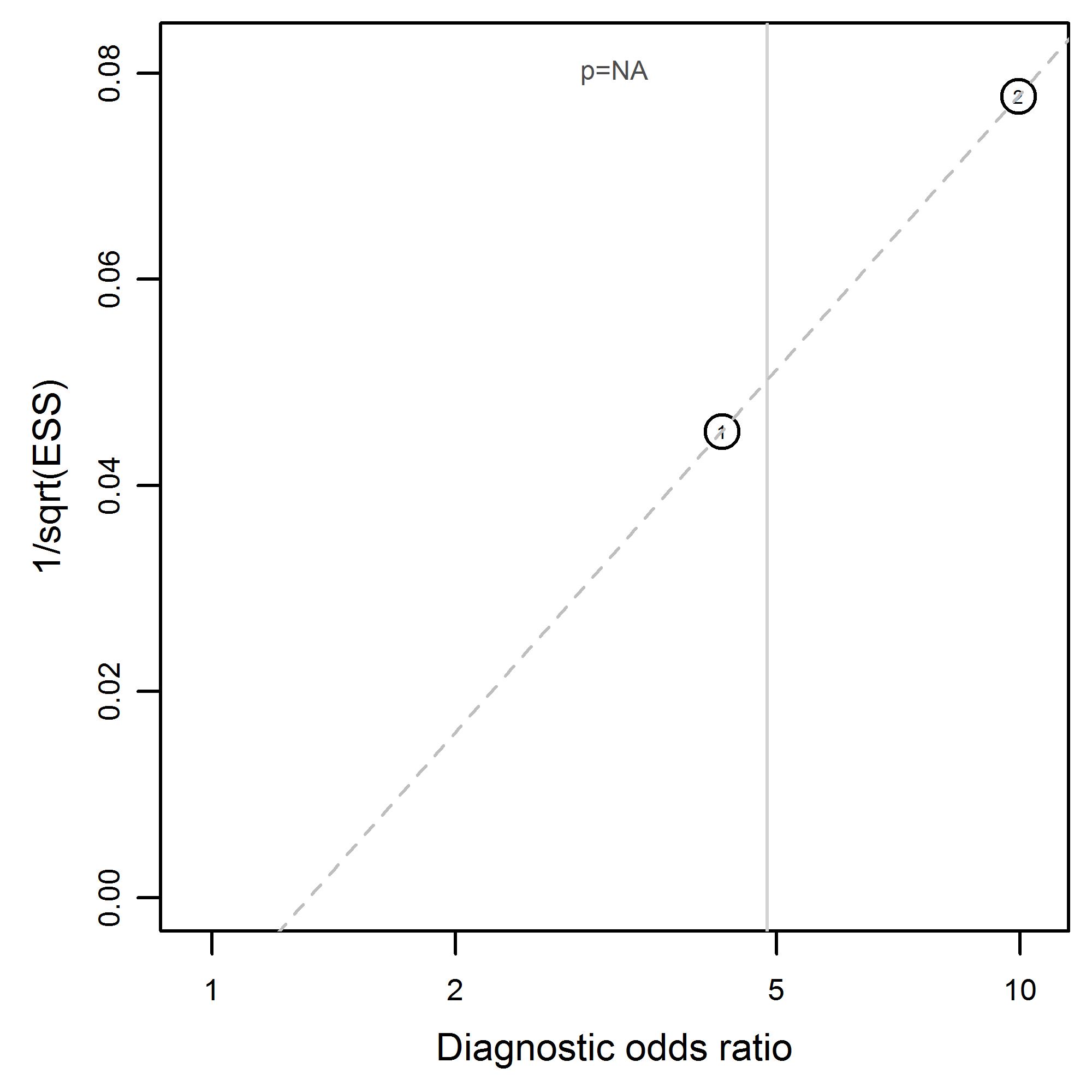 |
| Palpable step-off due to midfacial and mandibular fractures 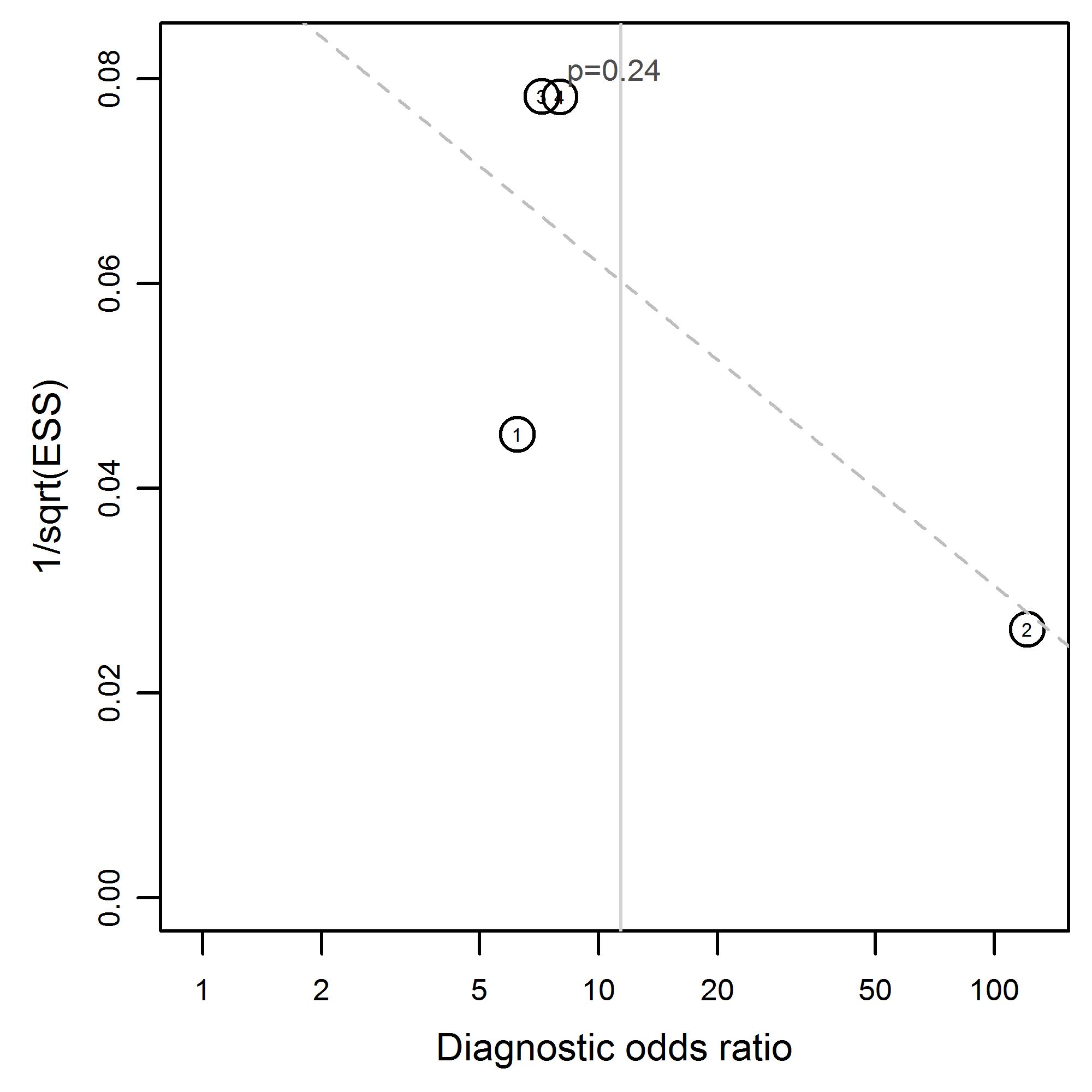 | Subconjunctival haemorrhage due to midfacial and mandibular fractures 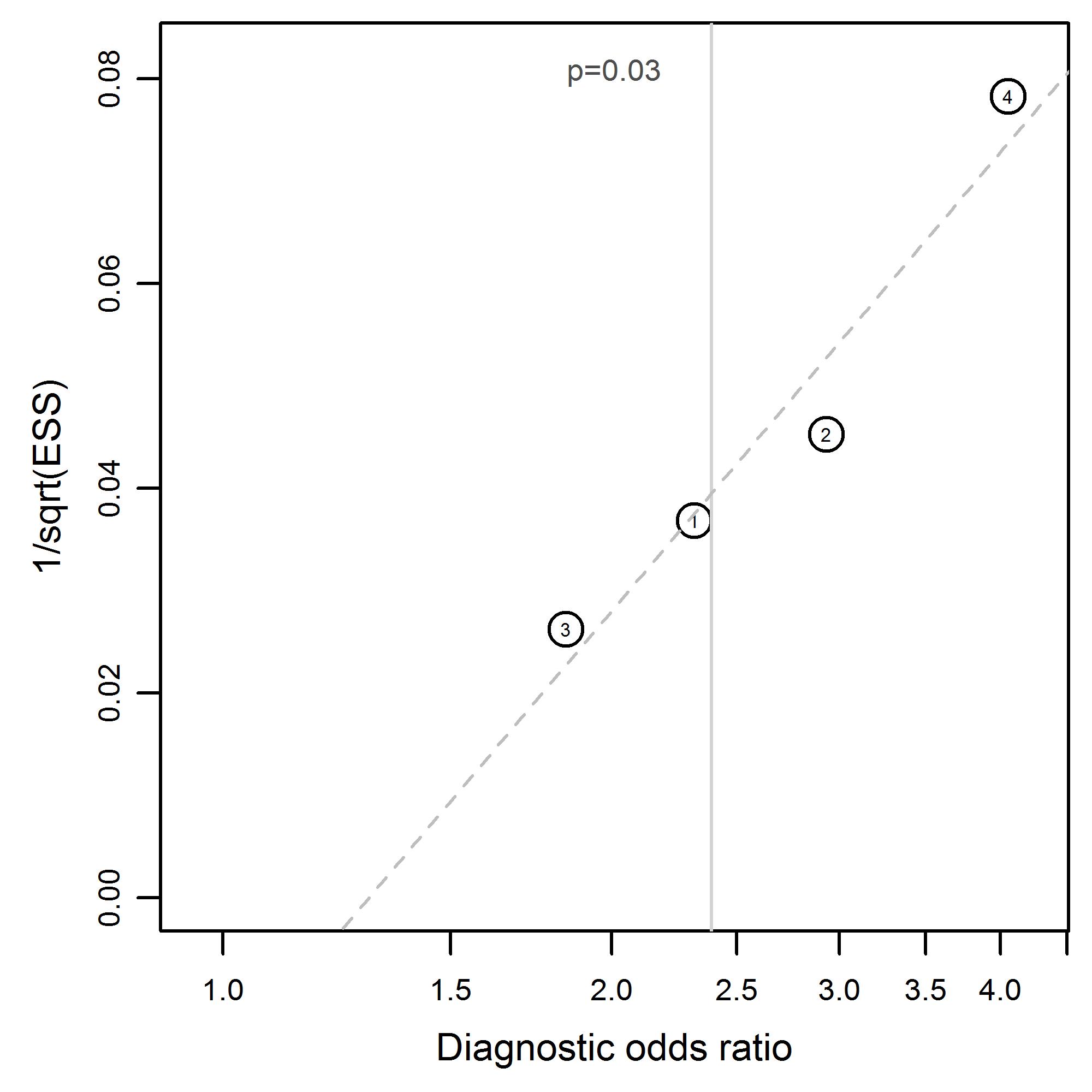 |
| Subconjunctival haemorrhage due to orbital fractures 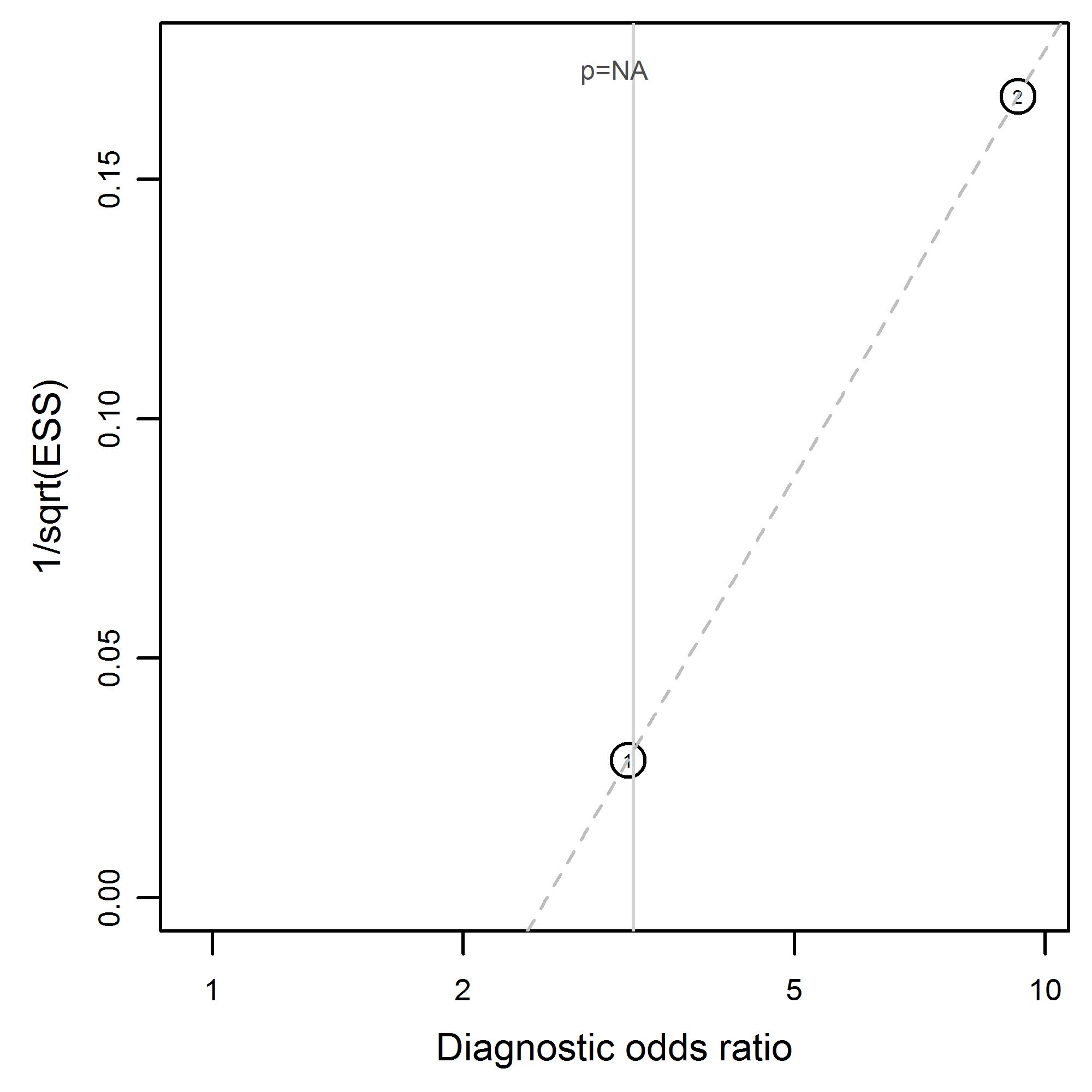 | Swelling due to midfacial and mandibular fractures 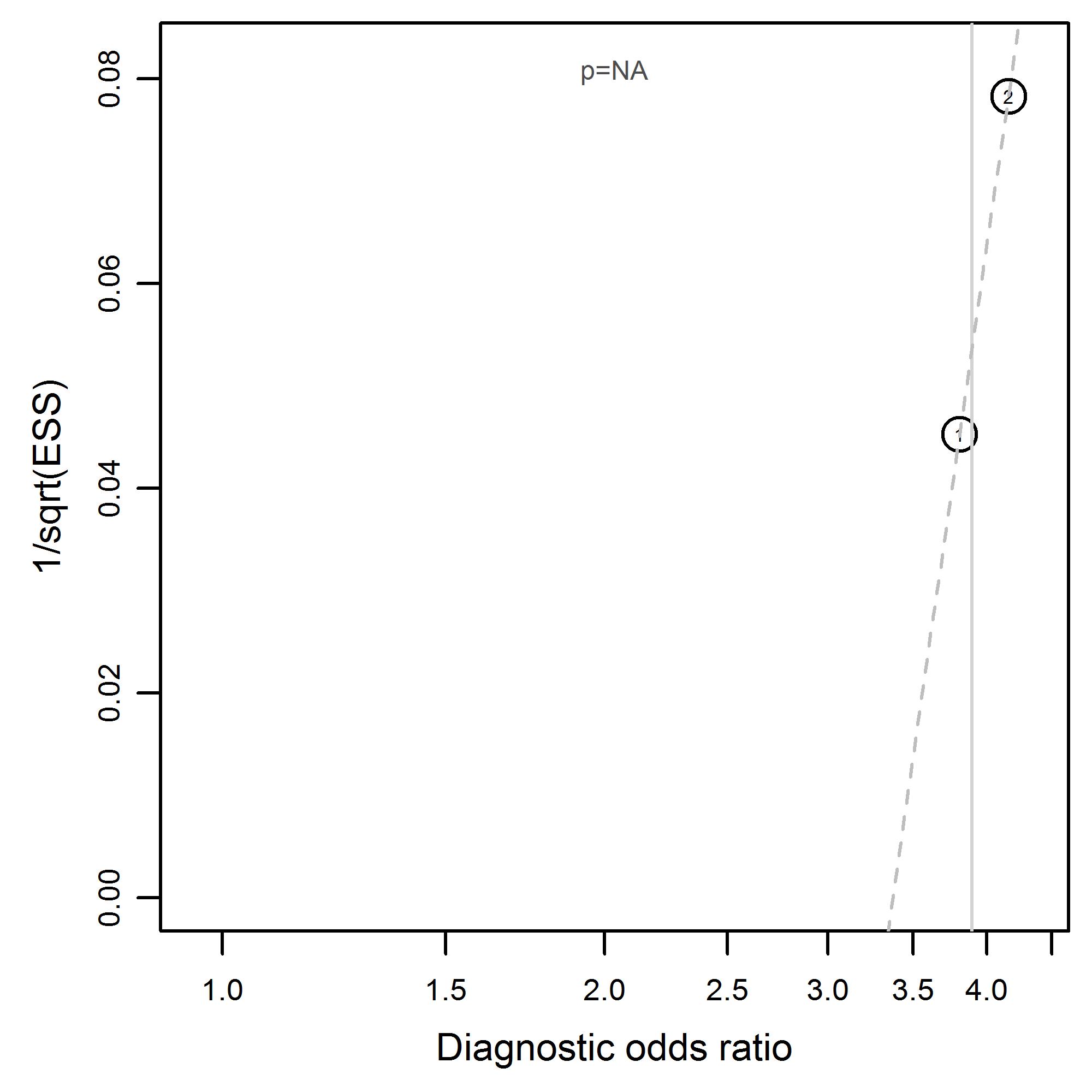 |
| Swelling or hematoma (peri-orbital) due to midfacial and mandibular fractures 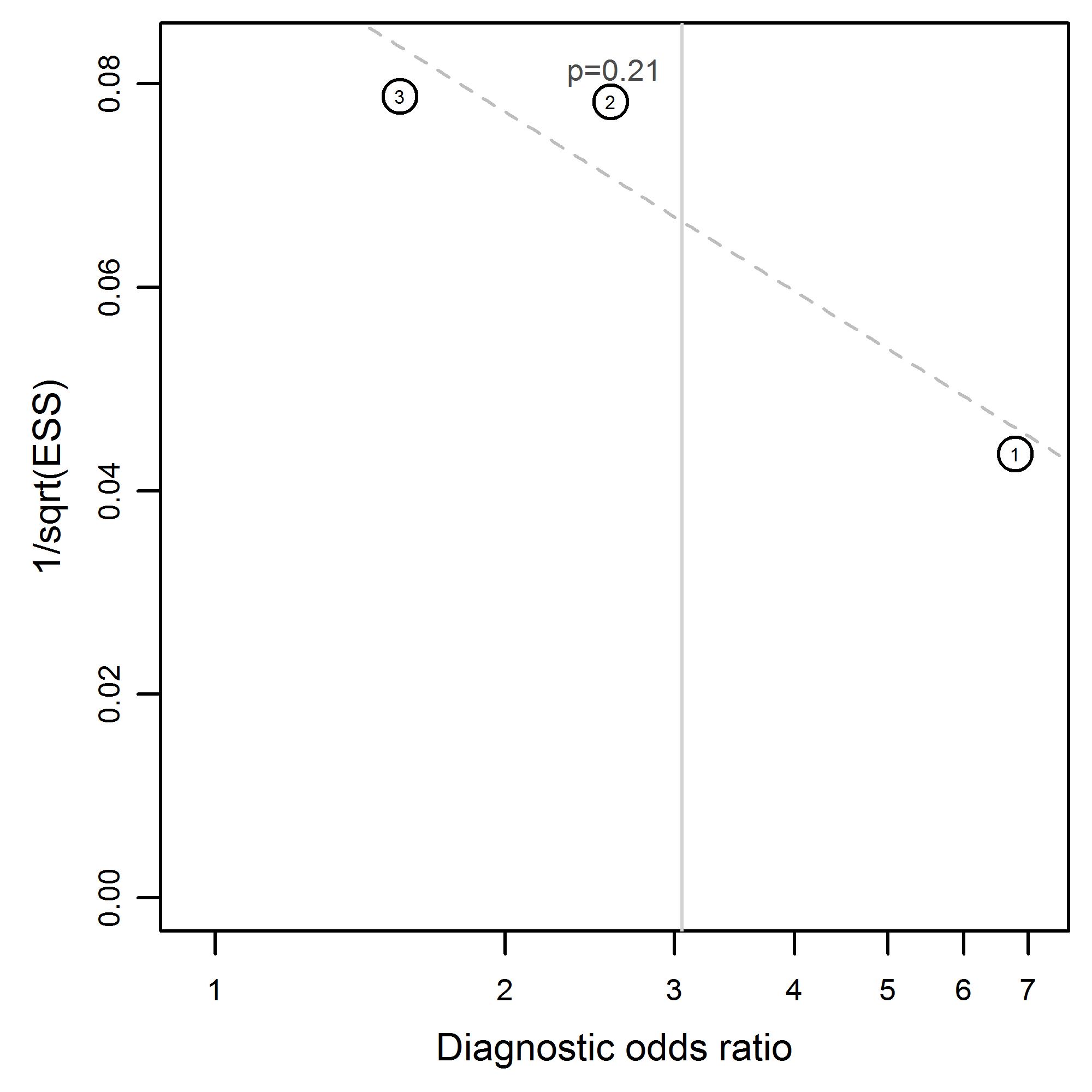 | Tooth avulsion due to midfacial and mandibular fractures 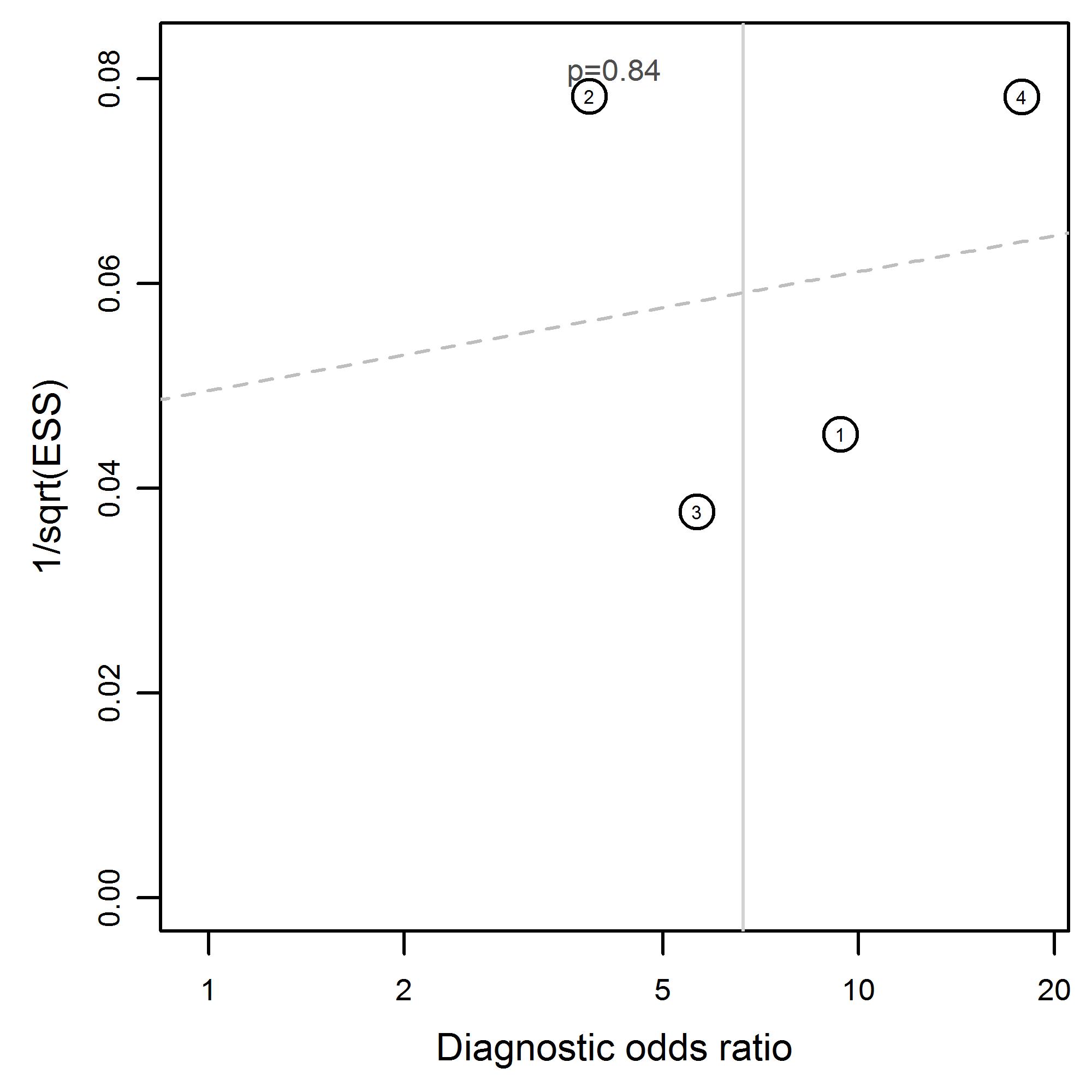 |
| Visual acuity change with midfacial and mandibular fractures 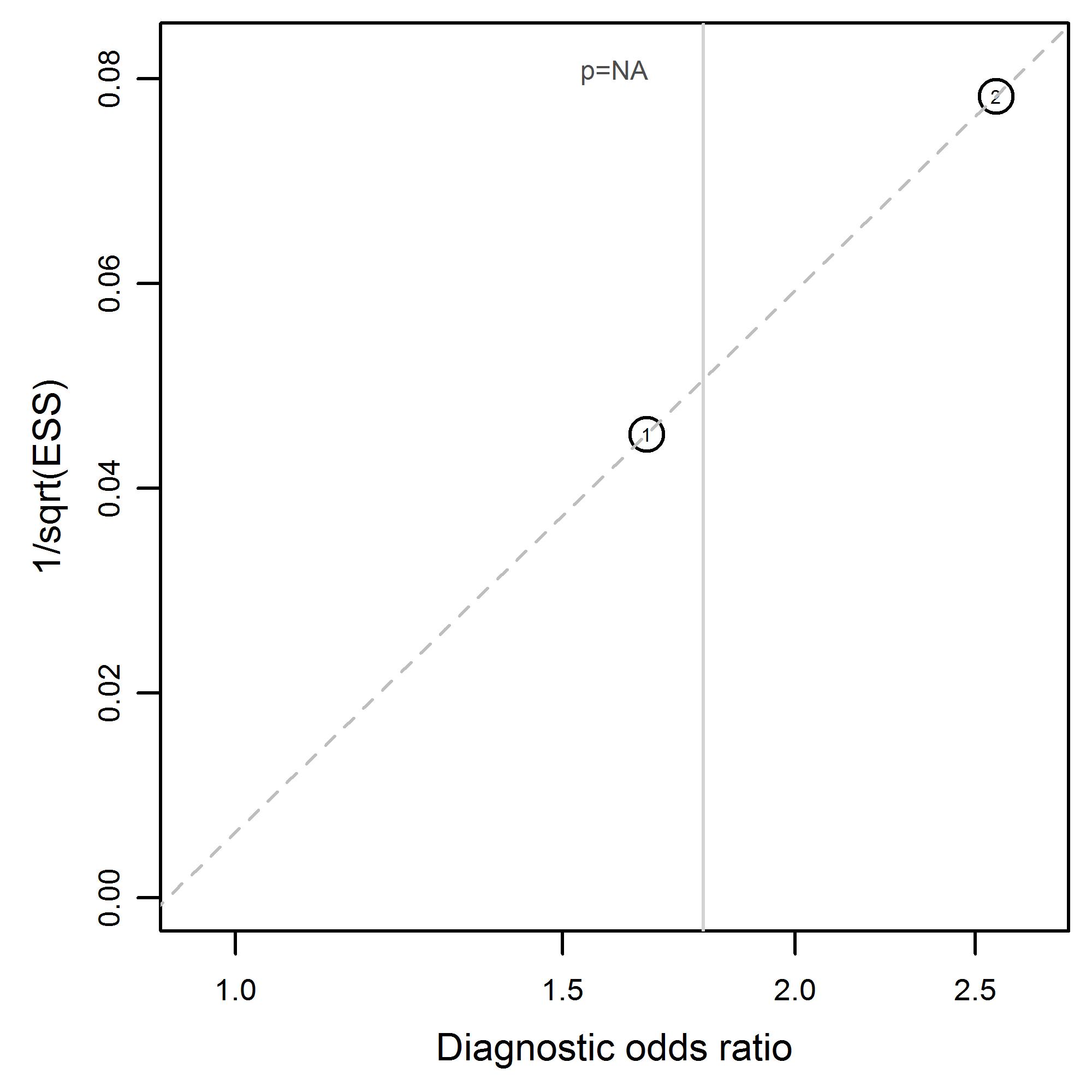 |  |

**Supplementary material S5**: Contingency tables for each individual reported physical examination finding and clinical decision aid.

*See separate excel file.*

**References**

1. Allison JR, Kearns A (2017) To scan or not to scan - a service improvement project for CT in facial trauma. Br J Oral Maxillofac Surg 55:e129–e130. https://doi.org/10.1016/j.bjoms.2017.08.128

2. Aslan F, Ozen O (2019) Correlation of Clinical Findings With Computed Tomography in Orbital Traumas. J Craniofac Surg 30:e586–e590. https://doi.org/10.1097/SCS.0000000000005583

3. Coloma OB, Moreno RO, Molina CV, et al (2013) Evaluation of clinical and imaging findings in patients with orbital fracture and its correlation with biomechanical factors of trauma. Int J Oral Maxillofac Surg 42:1219–1220. https://doi.org/10.1016/j.ijom.2013.07.169

4. Boffano P, Roccia F, Gallesio C, et al (2014) Diplopia and Orbital Wall Fractures. J Craniofac Surg 25:e183–e185. https://doi.org/10.1097/SCS.0000000000000437

5. Chow J, Parthasarathi K, Mehanna P, Whist E (2018) Primary Assessment of the Patient With Orbital Fractures Should Include Pupillary Response and Visual Acuity Changes to Detect Occult Major Ocular Injuries. J Oral Maxillofac Surg 76:2370–2375. https://doi.org/10.1016/j.joms.2018.04.024

6. Daniel M, Raghavan U (2005) Relation between epistaxis, external nasal deformity, and septal deviation following nasal trauma. Emerg Med J 22:778–779. https://doi.org/10.1136/emj.2004.018994

7. Etufugh N, Glickman R (2000) Correlation of Computerized Tomography and Flat Film Radiographic Findings With Clinical Examination in Patients Sustaining Periorbital Trauma. J Craniomaxillofac Trauma 6:17–19

8. Finnerty NM, Caterino JM (2016) Computed Tomographic Imaging in the Assessment of Blunt Facial Injury in the Emergency Department. Ann Emerg Med 68:S45–S46. https://doi.org/10.1016/j.annemergmed.2016.08.126

9. Gerlock A, Sinn D (1977) Anatomic, clinical, surgical, and radiographic correlation of the zygomatic complex fracture. Am J Roentgenol 128:235–238. https://doi.org/10.2214/ajr.128.2.235

10. Gunasekeran D V, Tan P, Goh E-S (2014) Evaluation and management outcomes of orbital wall fractures presenting to a tertiary hospital in Singapore. Invest Ophthalmol Vis Sci 55:2785

11. D.V. G, P. T, E.S. G (2014) Evaluation of cases of blunt ocular trauma presenting to a tertiary hospital in singapore. Ann. Acad. Med. Singapore

12. Haworth S, Bates A, Beech A, Knepil G (2017) A clinical decision rule to predict zygomatico-maxillary fractures. J Cranio-Maxillofacial Surg 45:1333–1337. https://doi.org/10.1016/j.jcms.2017.05.016

13. Holmgren EP, Dierks EJ, Homer LD, Potter BE (2004) Facial computed tomography use in trauma patients who require a head computed tomogram. J Oral Maxillofac Surg 62:913–918. https://doi.org/10.1016/j.joms.2003.12.026

14. Pérez-Guisado J, Maclennan P (2012) Clinical evaluation of the nose: a cheap and effective tool for the nasal fracture diagnosis. Eplasty

15. Siritongtaworn C, Pathanasri T (2020) A simple scoring system to predict zygomatic fracture. J Med Assoc Thail

16. Thai KN, Hummel RP, Kitzmiller WJ, Luchette FA (1997) The role of computed tomographic scanning in the management of facial trauma. J Trauma 43:214–7; discussion 217-8. https://doi.org/10.1097/00005373-199708000-00002

17. Timashpolsky A, Sayeed SM, Romeiser JL, et al (2015) A prospective analysis of physical exam findings in the diagnosis of facial fractures. J Am Coll Surg 221:e116. https://doi.org/10.1016/j.jamcollsurg.2015.08.208

18. Welman T, Shanmugarajah K, Sabah S, et al (2016) Assessment of Emergency Department Eye Examinations in Patients Presenting with Mid-Face Injury. J Emerg Med 50:422–426. https://doi.org/10.1016/j.jemermed.2015.07.041

19. Whitesell RT, Steenburg SD, Shen C, Lin H (2015) Facial fracture in the setting of whole-body CT for trauma: Incidence and clinical predictors. Am J Roentgenol 205:W4–W10. https://doi.org/10.2214/AJR.14.13589

20. Yadav K, Cowan E, Haukoos JS, et al (2011) Derivation of a Clinical Decision Rule for Computed Tomography After Orbital Trauma. Acad Emerg Med 18:S47. https://doi.org/10.1111/j.1553-2712.2011.01073.x
